# Subchronic toxic effects of bisphenol A on the gut-liver-hormone axis in rats via intestinal flora and metabolism

**DOI:** 10.3389/fendo.2024.1415216

**Published:** 2024-08-29

**Authors:** Jiaqi Wang, Ce Su, Mingqin Qian, Xin Wang, Changlan Chen, Yangcheng Liu, Wei Liu, Zheng Xiang, Baoli Xu

**Affiliations:** ^1^ School of Pharmaceutical Science, Liaoning University, Shenyang, China; ^2^ Shenyang Key Laboratory for Causes and Drug Discovery of Chronic Diseases, Shenyang, China; ^3^ Pharmacy Department, Shenyang Tenth People’s Hospital, Shenyang, China; ^4^ Department of Ultrasound, People’s Hospital of Liaoning Province, Shenyang, China; ^5^ Department of Pharmacy, Affiliated Zhongshan Hospital of Dalian University, Dalian, China

**Keywords:** bisphenol A, gut-liver axis, toxicity mechanism, intestinal flora, metabolism

## Abstract

**Background:**

Bisphenol A (BPA), a characteristic endocrine disruptor, is a substance that seriously interferes with the human endocrine system and causes reproductive disorders and developmental abnormalities. However, its toxic effects on the gut-liver-hormone axis are still unclear.

**Method:**

Male and female rats were exposed to BPA (300 mg/kg) by oral gavage for 60 consecutive days. H&E staining was used for histopathological evaluation, and the serum biochemical indexes were determined using an automatic analyzer. The 16S rRNA gene sequencing was used to detect the intestinal microbial diversity, and the GC-MS was used to analyze the contents of short-chain fatty acids (SCFAs) in colon contents. UPLC-QTOF MS was used to analyze the related metabolites. The ELISA method was used to assess the levels of serum inflammatory factors.

**Results:**

Histopathological analysis indicated that the liver, heart, and testis were affected by BPA. There was a significant effect on alanine aminotransferase (ALT), triglyceride (TG), total cholesterol (TC), and low-density lipoprotein (LDL) in the male-BPA group (P < 0.05), and globulin (GLB), indirect bilirubin (IBIL), alkaline phosphatase (ALP), ALT, TG, TC, high-density lipoprotein (HDL), and creatinine (Cr) in the female-BPA group (P < 0.05). Metagenomics (16S rRNA gene sequencing) analysis indicated that BPA reduced the diversity and changed the composition of gut microbiota in rats significantly. Compared with the control and blank groups, the contents of caproic acid, isobutyric acid, isovaleric acid, and propanoic acid in the colon contents decreased in the male-BPA group (P < 0.05), and caproic acid, isobutyric acid, isovaleric acid, and valeric acid in the colon contents decreased in the female-BPA group (P < 0.05). Metabolomic analysis of the serum indicated that BPA could regulate bile acid levels, especially ursodeoxycholic acid (UDCA) and its conjugated forms. The contents of amino acids, hormones, and lipids were also significantly affected after exposure to BPA. The increase in interleukin-6 (IL-6), interleukin-23 (IL-23), and transforming growth factor-β (TGF-β) in the serum of the male-BPA group suggests that BPA exposure affects the immune system.

**Conclusion:**

BPA exposure will cause toxicity to rats via disrupting the gut-liver-hormone axis.

## 1 Introduction

BPA is a plasticizer commonly used in the manufacture of plastic bottles, food, toys and other supplies (1). It is widely found in the environment and contaminates water, dust, sewage, and air (2). BPA exposure in humans occurs through the respiratory pathway, gastrointestinal tract, skin contact, and food and water contaminated with BPA (3, 4). It can be widely distributed throughout the body after human exposure, which can been detected in serum, urine, umbilical cord blood, amniotic fluid, and placental tissue (5). Global production of BPA was 6.2 million tons (MT) in 2020, which exhibited a gradually increasing trend and is expected to reach 7.1 MT by 2027 (6). There are many ways for BPA to enter the aquatic environment, including direct and disorderly discharges during production and manufacturing, contaminated soil being introduced into the aquatic environment through stormwater wash-off or surface runoff, and discarded plastics that can leach BPA into water. Finally, BPA can be detected in water environments including rivers (1800-107700 ng L^−1^), lakes (1260-106900 ng L^−1^), and drinking water (5.3-128 ng L^−1^) (7–11). BPA can replace endogenous hormones and bind to receptors, altering hormone levels in the bloodstream (12). It can also disrupt the function and development of the reproductive system. A recent study has linked BPA exposure to increased levels of serum luteinizing hormone (LH), estradiol (E2), progesterone, and testosterone, as well as decreased cortisol levels (13). BPA intake has also been linked to a higher risk of various cancers, such as breast, ovarian, uterine, prostate, and testicular cancer (14). Because of these adverse effects on human health, several countries have banned the addition of BPA to infant and children’s products (15).

The ‘gut-liver axis’ was formally proposed in 1998 as a complex, interlinked network that involves the interaction between various cytokines and inflammatory mediators between the intestine and liver. It is an important pathway between the intestine and liver that can be regulated in both directions (16). Its changes affect the nutrients, microbial antigens, metabolites, and bile acids in the host (17); regulate the metabolism and immune response in the gut and liver (18); and affect the structure and function of their respective microbial communities (19).

In recent years, research on the interaction between the intestinal flora and the reproductive system has shown that the intestinal flora and hormones regulate each other (20). Intestinal bacteria play an important role in estrogen metabolism. It can metabolize estrogens into deconjugated forms by secreting β-glucuronidase (21). It is possible that gut microbes participate in the regulation of sex hormones and, conversely, that sex hormones modify microbial diversity (22). Based on the close relationship between sex hormones and intestinal flora, the ‘gut-liver-hormone axis’ was proposed on the basis of the gut-liver axis to elaborate on the influence of external factors on organisms from the aspects of liver, intestine, and hormone levels. Studies have shown that BPA causes hepatotoxicity by affecting genes involved in oxidative phosphorylation and fatty acid metabolism in the liver (23). Intestinal flora is a significant component of the gut-liver axis. Changes in the abundance and types of intestinal flora can also reflect the effects of toxic substances on the body. There is a significant relationship between disruption of the gut microbiota and imbalances in related intestinal small molecule metabolites (24).

Bile acid metabolism is intricately linked to the intestinal flora and liver (25). Primary bile acids are produced by cholesterol in the liver, then catalyzed by sterol 12α-hydroxylase and sterol 27α-hydroxylase to synthesize cholic acid (CA) and chenodeoxycholic acid (CDCA) (26). Intestinal flora can regulate the composition and physicochemical properties of bile acids by metabolizing bile acids, and further regulate the synthesis and transport of bile acids through the effect of specific bile acids on bile acid receptors (27). Bile acids control intestinal microbial overgrowth and affect the composition of intestinal flora through antibacterial and cytotoxic effects (65). This mutual regulation causes the total amount and composition of bile acids and the abundance and diversity of intestinal flora to maintain a balanced state.

In addition to bile acids, there are numerous small molecule metabolites that are closely associated with intestinal flora and the gut-liver-hormone axis. Short-chain fatty acids (SCFAs) are the primary metabolites produced by bacteria through the fermentation of dietary fiber in the gastrointestinal tract (28). These SCFAs have the ability to regulate various physiological and biochemical functions of the host. They contribute to maintaining the natural intestinal barrier function, intestinal motility, intestinal hormone secretion, chromatin regulation, intestinal-brain axis, and immune function (29).

In this study, an integrated strategy that combines toxicology, intestinal flora analysis, and metabolomics was used to systematically assess the subchronic toxicity of BPA. Through comprehensive data analysis, we investigated the effects of BPA exposure on the gut-liver-hormone axis, which provided a novel perspective on the toxic mechanisms of pollutants in water environments.

## 2 Material and methods

### 2.1 Reagents

BPA was purchased from Jizhi Biochemical Technology Co., Ltd (Shanghai, China). Pentobarbital sodium was purchased from Sigma-Aldrich Co., Ltd (Shanghai, China). Physiological saline (NaCl 0.9%) was obtained from Harbin Sanlian Pharmaceutical Co., Ltd (Harbin, China). Carbamazepine and mycophenolic acid were purchased from Anpel Laboratory Technologies (Shanghai, China) Inc (Shanghai, China). Chemical references including bile acids, amino acids, lipids, and hormones were purchased from Yuanye Biotechnology Co., Ltd (Shanghai, China), Anpel Laboratory Technologies (Shanghai, China), and Jizhi Biochemical Technology Co., Ltd (Shanghai, China), respectively.

### 2.2 Animal exposure

Male and female Sprague Dawley (SD) rats aged 6 weeks (200 ± 20 g) were purchased from Liaoning Changsheng Biotechnology Co., Ltd. (SCXK (LN) 2020-0001). The study and included experimental procedures were approved by the institutional animal care and use committee of Liaoning University (Approval No. 20200723512). Rats were housed under the following conditions: 12-hour light/12-hour dark cycle, temperature 23 ± 2°C, and unrestricted access to food and drinking water.

In a previous study, the LD_50_ value of BPA was established as 3250 mg/kg (30). Thus, in this study, 300 mg/kg, which is the 1/10 LD_50_ of BPA, was used as the dose according to the “Technical Guidelines for Long-term Toxicity Testing of Chemical Drugs ([H]-GPT2-1)” issued by the State Drug Administration of China (2005).

**Figure 1:**
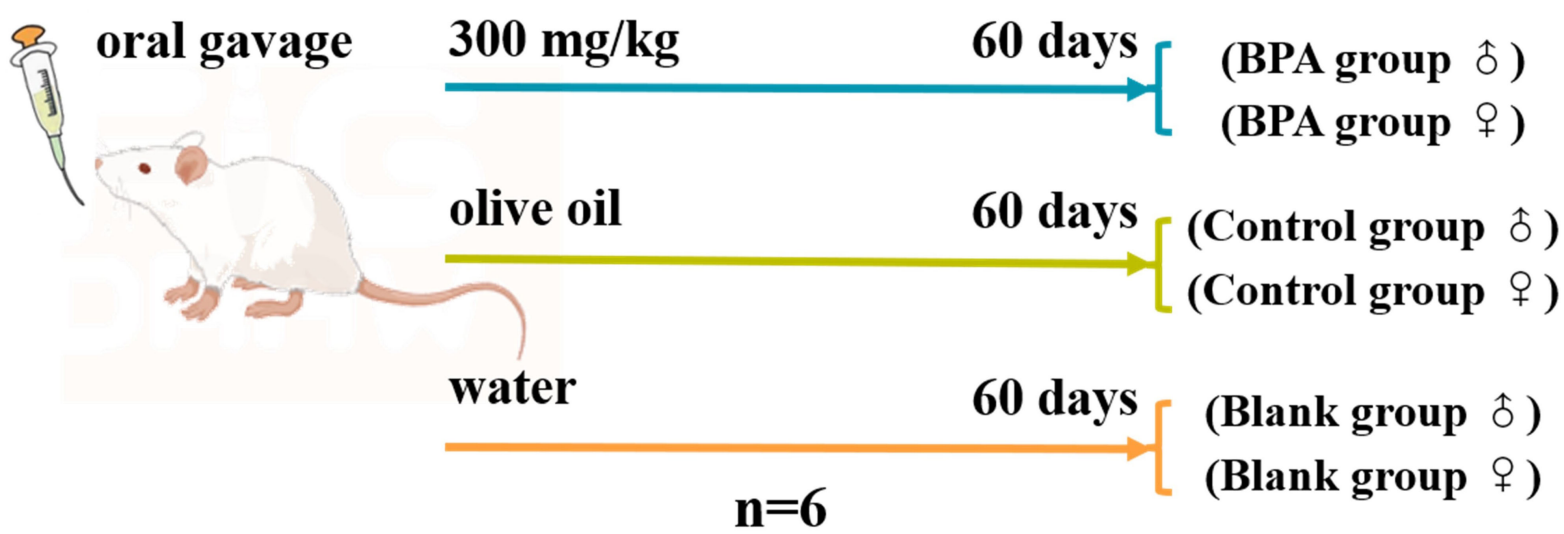
Design of BPA exposure experiment.

In total, 36 rats including 18 male rats and 18 female rats were randomly divided into 6 groups (n=6), the specific groups are shown in **Figure 1**. BPA was dissolved in olive oil as an administration group. The same volume of normal saline and olive oil were given in the blank and control groups, respectively. After 1-week adaptive feeding, oral gavage was performed daily for 60 consecutive days after 6 h of fasting. Body weights were recorded weekly and exhaustive observations were recorded weekly during the experiment. On the 61^st^ day, the rats were anesthetized with pentobarbital sodium by intraperitoneal injection (50 mg/kg). Blood samples were collected from the abdominal aorta, and then placed for 30 min. The serum samples were obtained by centrifugation at 3000 rpm for 15 min at 4°C. Heart, liver, spleen, kidney, testis, and uterus were collected and fixed with 4% paraformaldehyde for histopathological analysis. Rat feces were carefully collected from the rectum, and the serum and fecal samples were stored at -80°C until further analysis.

For each tissue, the average of three slides per rat was used as the independent data for statistical analysis. Marking criterion were as follows: 0, no pathological changes; 1, mild; 2, moderate; 3, severe (31). Data were analyzed using two-way ANOVA and Student’s t test.

### 2.3 Serum biochemical analysis

The contents of total protein (TP), albumin (ALB), globulin (GLB), total bilirubin (TBIL), direct bilirubin (DBIL), indirect bilirubin (IBIL), alkaline phosphatase (ALP), alanine aminotransferase (ALT), aspartate aminotransferase (AST), triglyceride (TG), total cholesterol (TC), high-density lipoprotein (HDL), low-density lipoprotein (LDL), urea, creatinine (Cr), glucose (GLU), and glomerular filtration rate (GFR) were determined using a Cobas8000 automatic analyzer (Roche, USA).

### 2.4 16S rRNA sequencing of colonic contents

Total genomic DNA samples were extracted using the OMEGA Soil DNA Kit (M5635-02) (Omega Bio-Tek, Norcross, GA, USA) following the manufacturer’s instructions, and stored at -20°C prior to further analysis. The quantity and quality of extracted DNA were measured using a NanoDrop NC2000 spectrophotometer (Thermo Fisher Scientific, Waltham, MA, USA) and agarose gel electrophoresis, respectively. PCR amplification of the bacterial 16S rRNA genes V3–V4 region was performed using the forward primer 338F (5’-ACTCCTACGGGAGGCAGCA-3’) and the reverse primer 806R (5’-GGACTACHVGGGTWTCTAAT-3’). PCR amplicons were purified with Vazyme VAHTSTM DNA Clean Beads (Vazyme, Nanjing, China) and quantified using the Quant-iT PicoGreen dsDNA Assay Kit (Invitrogen, Carlsbad, CA, USA). After the individual quantification step, amplicons were pooled in equal amounts, and pair-end 2×250 bp sequencing was performed using the Illlumina NovaSeq platform with a NovaSeq 6000 SP Reagent Kit (500 cycles) at Shanghai Personal Biotechnology Co., Ltd (Shanghai, China). The α-diversity and β-diversity were analyzed on the online platform of Personalbio Genescloud (https://www.genescloud.cn/).

### 2.5 SCFA analysis

SCFAs were detected by gas chromatography-mass spectrometry (GC-MS) (Agilent 7890B-5977B GC/MSD). Briefly, 100 mg of fecal contents were mixed with 50 μL of phosphoric acid (0.2%, v/v) aqueous solution containing 4-methylvaleric acid as internal standard (0.668 mg/mL), to be tested after sealing. The above pretreatment steps of rat feces samples were carried out at 4°C to prevent the volatilization of SCFAs.

The initial temperature was set to 60°C, and the temperature was first raised to 120°C at 30°C/min, then to 140°C at 5°C/min for 1 min, after that, raised to 150°C at 10°C/min for 1 min, then to 160°C at 5°C/min for 1 min, and finally, to 230°C at 35°C/min, where it was maintained for 5 min. Helium was used as the carrier gas at a flow rate of 1.0 mL/min. A 70 eV EI source was adopted, and the full scan and SIM scan modes were used.

### 2.6 Metabolomics analysis

UPLC-MS/MS analysis was carried out on an X500R QTOF LC-MS/MS system (AB Sciex, USA) equipped with ACQUITY UPLC ^®^ BEH C18 (1.7 μm, 2.1 × 100 mm, Waters, USA). The mobile phase was composed of water (solvent A) and methanol (solvent B) at 0.3 mL/min. The gradient elution program was set as follows: 0-5 min 70% (B), 5-13 min 70-98% (B), 13-15 min 98% (B), 15-15.1 min 98-50% (B), and 15.1-18 min 50% (B). The column temperature was 30°C, and the injection volume was 10 μL.

Furthermore, 150 μL of the serum was added into a 1.5-mL centrifuge tube with 150 μL of methanol containing carbamazepine and mycophenolic acid as internal standards at a concentration of 50 ng/ml, respectively. Subsequently, 600 μL of methanol was added into the centrifuge tube, vortexed for 30 s, and centrifuged at 12,000 rpm for 15 min (4°C). Thereafter, 700 μL of the supernatant was pipetted into a centrifuge tube and blow-dried with N_2_. The residues were reconstituted in 70 μL of 50% MeOH-H_2_O solution, vortexed for 30 s, and centrifuged at 12,000 rpm for 10 min. The supernatant was then filtered using a 0.22 μm membrane before analysis.

All target metabolite standards were dissolved in double distilled water or methanol to prepare 2 mg/mL stock solutions, which were diluted before use.

### 2.7 Enzyme-linked immunosorbent assay

The contents of rat interleukin-6 (IL-6), interleukin-10 (IL-10), interleukin-17 (IL-17), interleukin-23 (IL-23), and transforming growth factor-β (TGF-β) were determined using commercially available enzyme-linked immunosorbent assay (ELISA) Kits (Nanjing Jianchen Bioengineering Institute, Nanjing, China).

### 2.8 Statistical analysis

All data were subjected to a normal distribution test using SPSS 20.0 (SPSS, USA). Normal distribution data were processed using GraphPad Prism 6 (GraphPad Software, USA) and presented as mean ± standard deviation (SD). Difference analysis was performed using one-way analysis of variance (ANOVA) followed by Dunnett’s multiple comparison test. Statistical analysis for paired comparison groups was conducted using a two-tailed Student’s t-test. Non-normal distribution data were presented as median and range, and statistical significance was based on the Mann-Whitney U test. P < 0.05 was considered statistically significant.

## 3 Results

### 3.1 Effect of BPA on rats

The body weights of the rats were observed and recorded during the experiment. The initial body weight of all rats was within the range of 200 g ± 20 g. After 8 weeks of daily BPA exposure, there was no significant difference between the groups (P > 0.05, **Supplementary Figure S1**).

**Figure 2:**
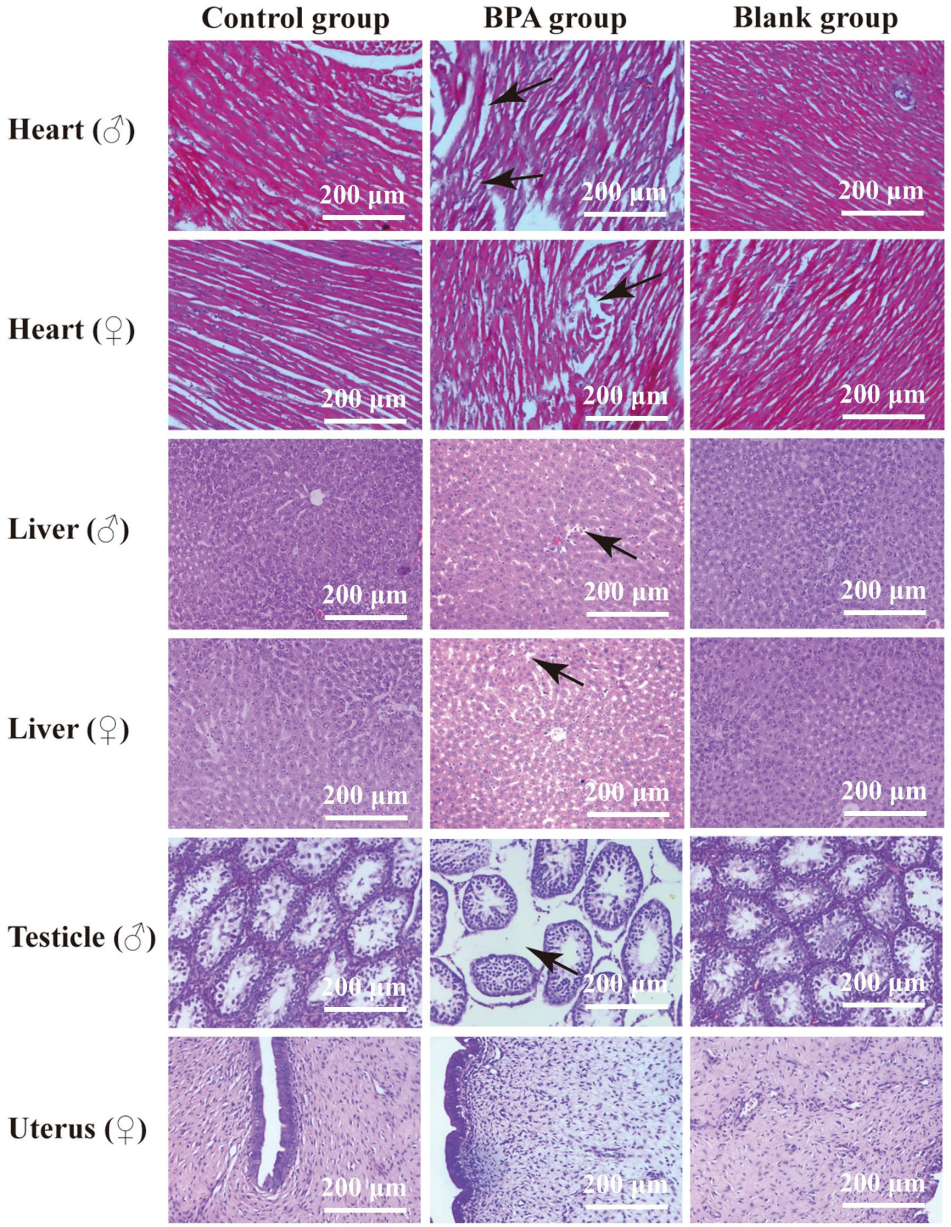
HE stains of the heart, liver, testicle and uterus in control group, BPA group (300 mg/kg) and blank group (200x). The main changes are marked in the figure (black arrow).

**Figure 3:**
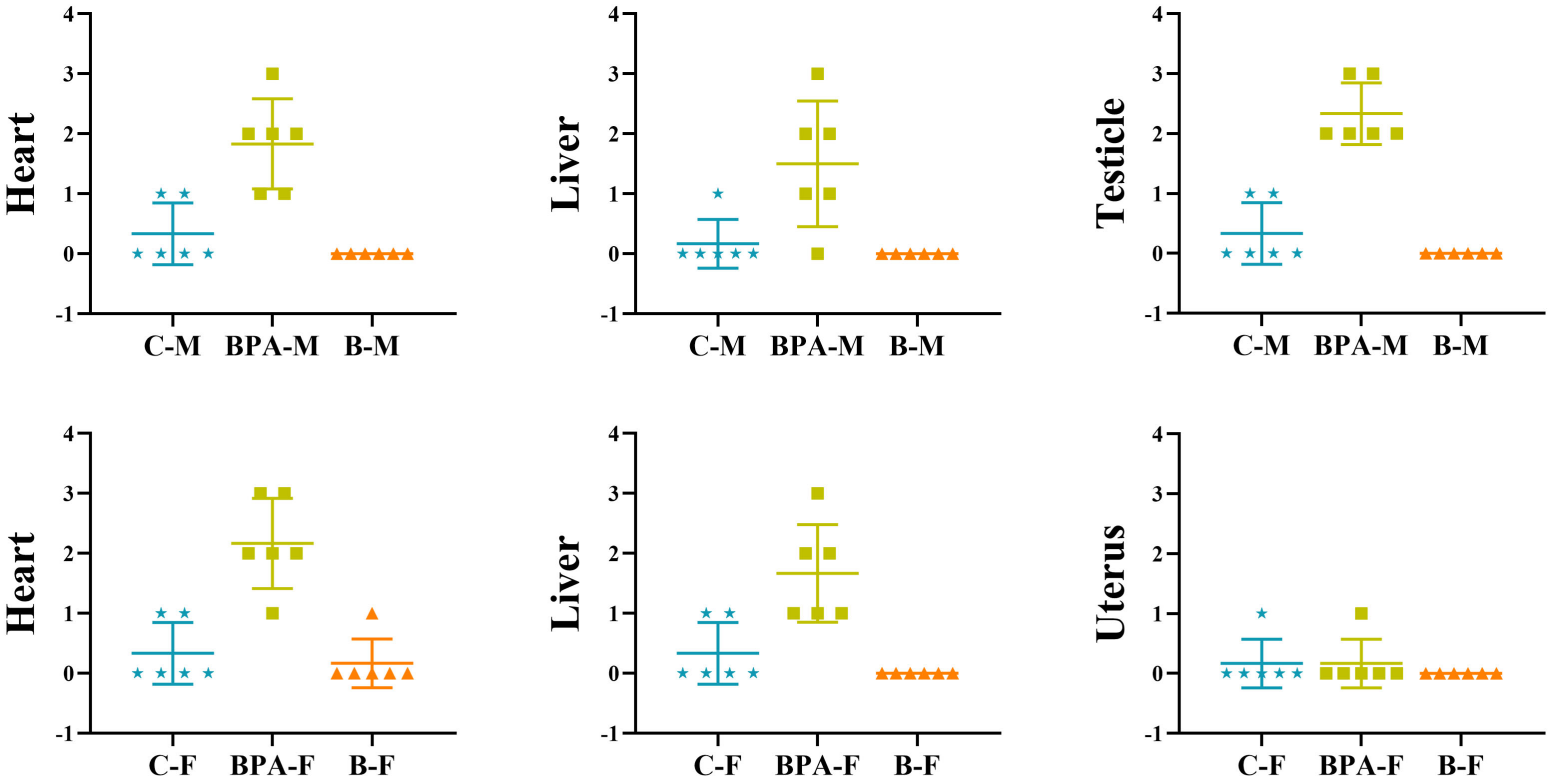
The pathological score of the heart, liver, testicles, and uterus in the control group, BPA group (300 mg/kg) and blank group. Data are mean ± SD. (n = 6), marking criterion: 0, normal; 1, mild; 2, moderate; 3, severe.

Compared with the blank and the control groups, the rat heart tissue in the BPA groups (♂ and ♀) showed cell swelling and edema degeneration, the myocardial cells were not densely arranged, and the irregular white voids increased. In the BPA groups, there was a local range of myocardial cell structure damage and muscle striation fracture, myocardial cell necrosis, and the phenomenon of nucleus release into the intercellular substance. HE staining of liver tissue indicated that the liver structure of the control group was clear, and the hepatocytes were radially arranged with rich cytoplasm and nuclei centered on the central vein. The gap between hepatocytes in the BPA groups (♂ and ♀) became larger and vacuolar degeneration, and the male group showed dilated congestion. These indicate that BPA exposure will cause damage to the livers of male and female rats. HE staining of testicle tissue indicated that there was obvious atrophy of the seminiferous tubules, the gap between the seminiferous tubules became larger, the arrangement was irregular, and the Leydig cells were also relatively reduced compared to that of the control and blank groups (**Figure 2**). The pathological scores of the heart, liver, testicles, and uterus were shown in **Figure 3**. There was no significant effect on the staining results of the uterus, kidneys and spleen (**Supplementary Figures S2**, **S3**).

### 3.2 Serum biochemical analysis

**Figure 4:**
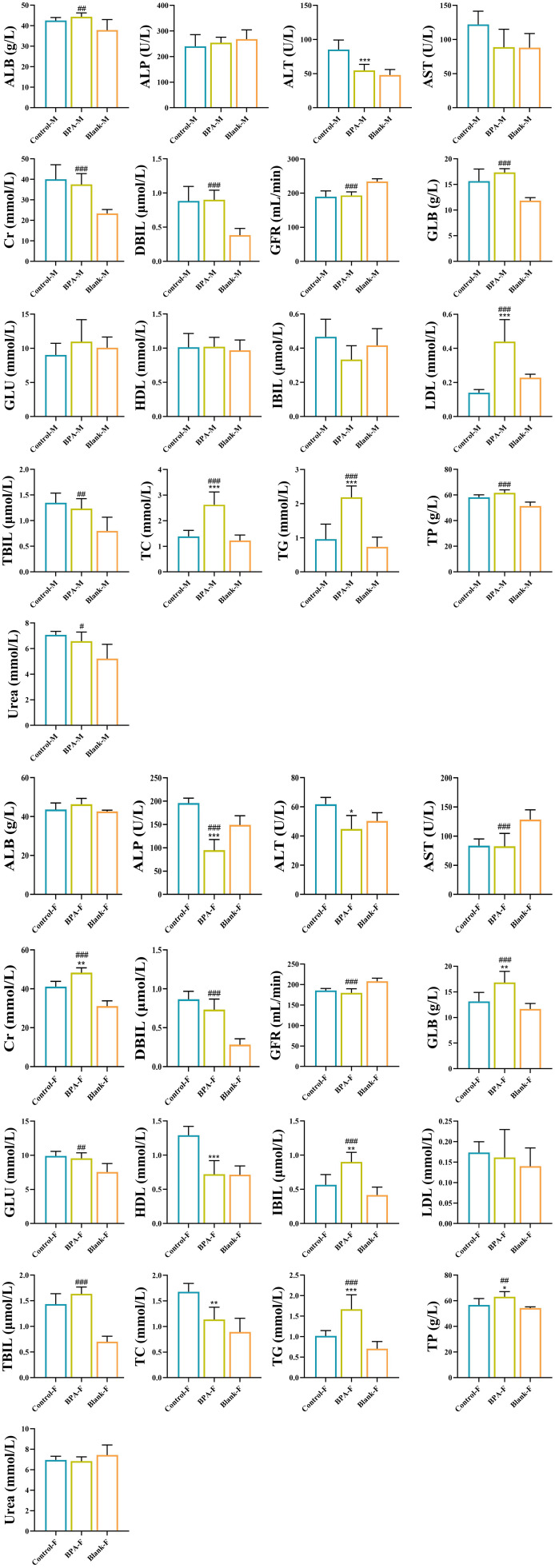
The effects of BPA (300 mg/kg) on 17 serum biochemical indexes in SD rats after 60 days of exposure. (Male group: Control-M, BPA-M, Blank-M; Female group: Control-F, BPA-F, Blank-F); n=6; ANOVA followed by Dunnett’s multiple comparison tests; the date is presented mean ± SD, *P < 0.05, **P < 0.01 and ***P < 0.001 versus the control group; ^#^P < 0.05, ^##^P < 0.01 and ^###^P < 0.001 versus the blank group.

In the BPA group (♂), the contents of TP, ALB, and GLOB in serum were significantly increased, but the ratio of ALB/GLOB was decreased, compared with that of the blank and control groups. Compared with the blank group and the control group, the contents of TG, TC, and LDL-C were significantly increased in the BPA group. In the BPA group (♀), the contents of TP and ALB were significantly increased, the ratio of ALB/GLOB was decreased, the contents of TBIL and IBIL were increased, and the contents of ALP, ALT, and AST were decreased. The creatinine (Cr) content increased significantly, and the ratio of Urea to Cr decreased significantly (**Figure 4**).

### 3.3 Analysis of intestinal flora

#### 3.3.1 Intestinal flora diversity and composition

**Figure 5:**
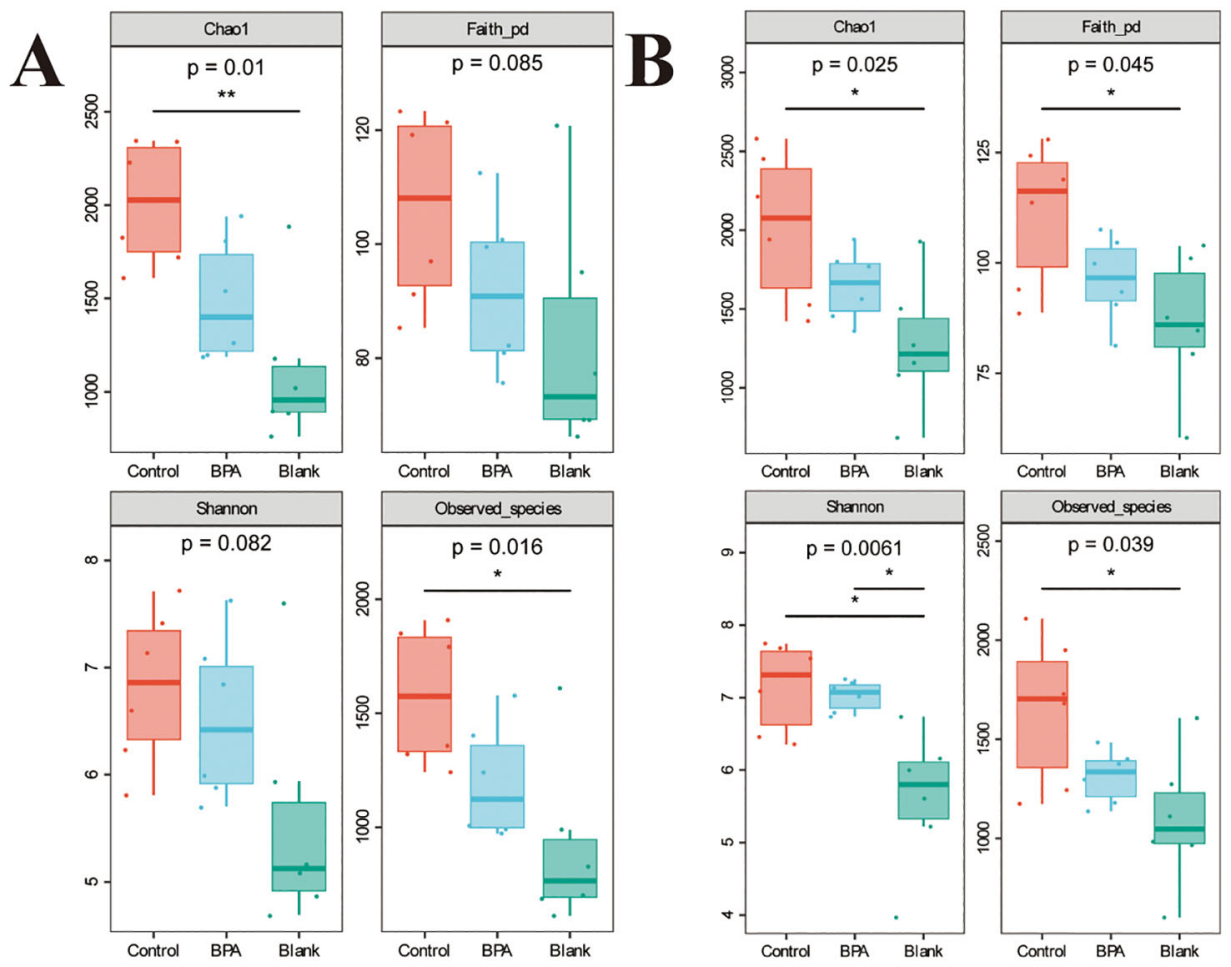
The effects of BPA (300 mg/kg) on four a diversity indexes in SD rats after 60 days-exposure. **(A)** male-BPA group; **(B)** female-BPA group; n=6; ANOVA; *P < 0.05, **P < 0.01.

**Figure 6:**
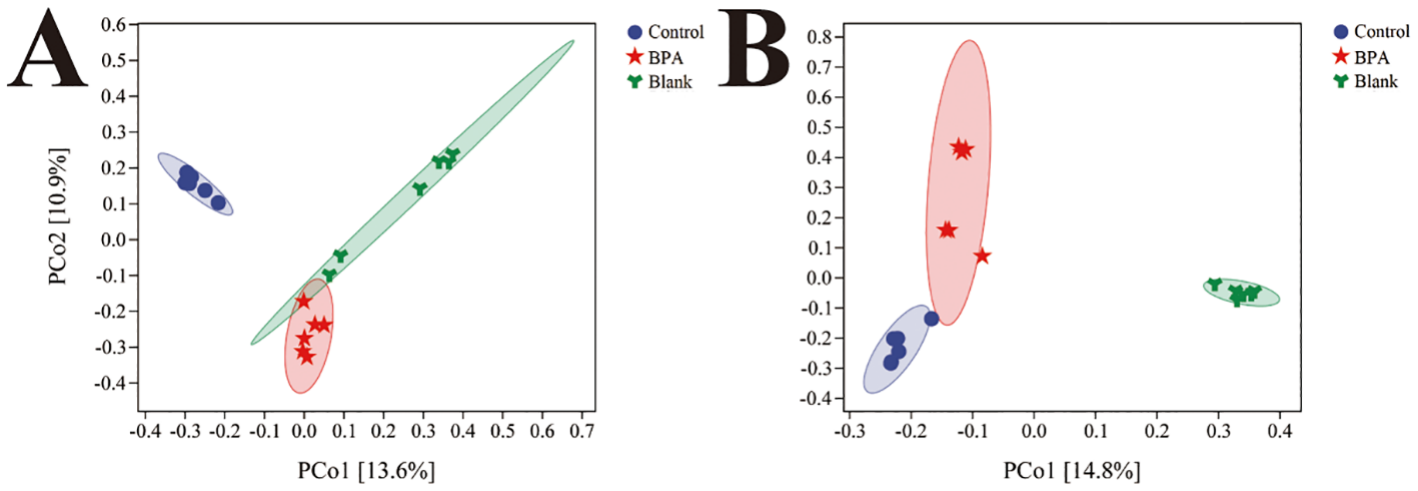
The NMDS analysis of bacterial structures for three groups **(A)** male groups; **(B)** female groups; n=6.

Based on the 16S rRNA gene data, the α diversity index was used to evaluate the number of operational taxonomic units (OTUs) and the community diversity in the sample (**Figure 5**). Compared with the control group, the Chao1 and observed species indexes both decreased in the BPA groups (♂ and ♀). Nonmetric multidimensional scaling (NMDS) was used to visualize the β diversity analysis. NMDS is a data analysis method that simplifies the research objects (samples or variables) of multidimensional space into low-dimensional space for positioning, analysis and classification, while retaining the original relationship between objects. β diversity analysis showed that the intestinal flora structure of the BPA group was significantly different from that of the control group (♂ and ♀, **Figure 6**), and the group gathered well, indicating that BPA altered the composition of intestinal flora in rats.

#### 3.3.2 Analysis of community structure and difference at the phylum level

**Figure 7:**
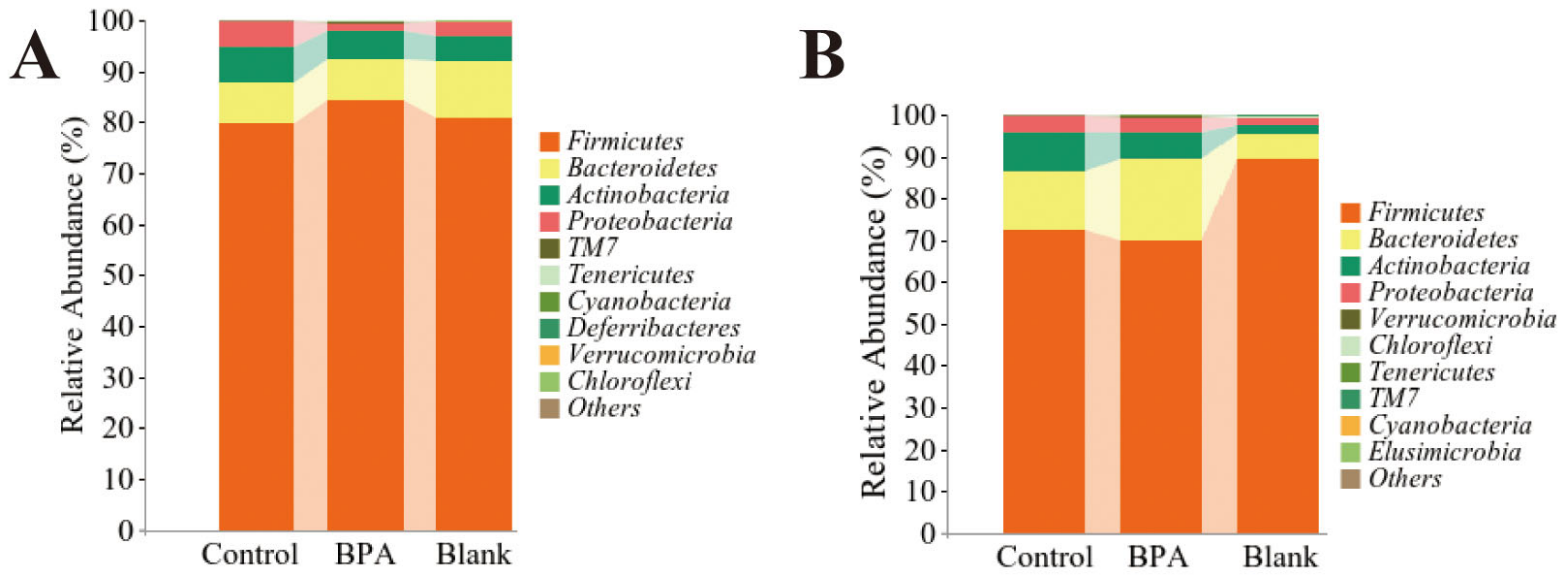
The metagenomics analysis at the phylum level. **(A)** male-BPA group; **(B)** female-BPA group; n=6.

The top 10 dominant bacteria of the relative abundance of fecal flora in the three groups of male rats were *Firmicutes, Bacteroidetes, Actinobacteria, Proteobacteria, TM7, Tenericutes, Cyanobacteria, Deferribacteres, Verrucomicrobia*, and *Chloroflexi* (**Figure 7**). The top 10 dominant bacteria of female rats among the three groups were *Firmicutes, Bacteroidetes, Actinobacteria, Proteobacteria, Verrucomicrobia, Chloroflexi, Tenericutes, TM7, Cyanobacteria*, and *Elusimicrobia* (**Figure 7**).

LEfSe (LDA Effect Size) multi-level species difference discriminant analysis is an analytical tool for discovering and interpreting biomarkers of high-dimensional data by linear discriminant analysis (LDA), the blue nodes and red nodes in the branches represent the bacteria that are enriched in the control group and the BPA group and have a significant effect on the differences between the groups.

**Figure 8:**
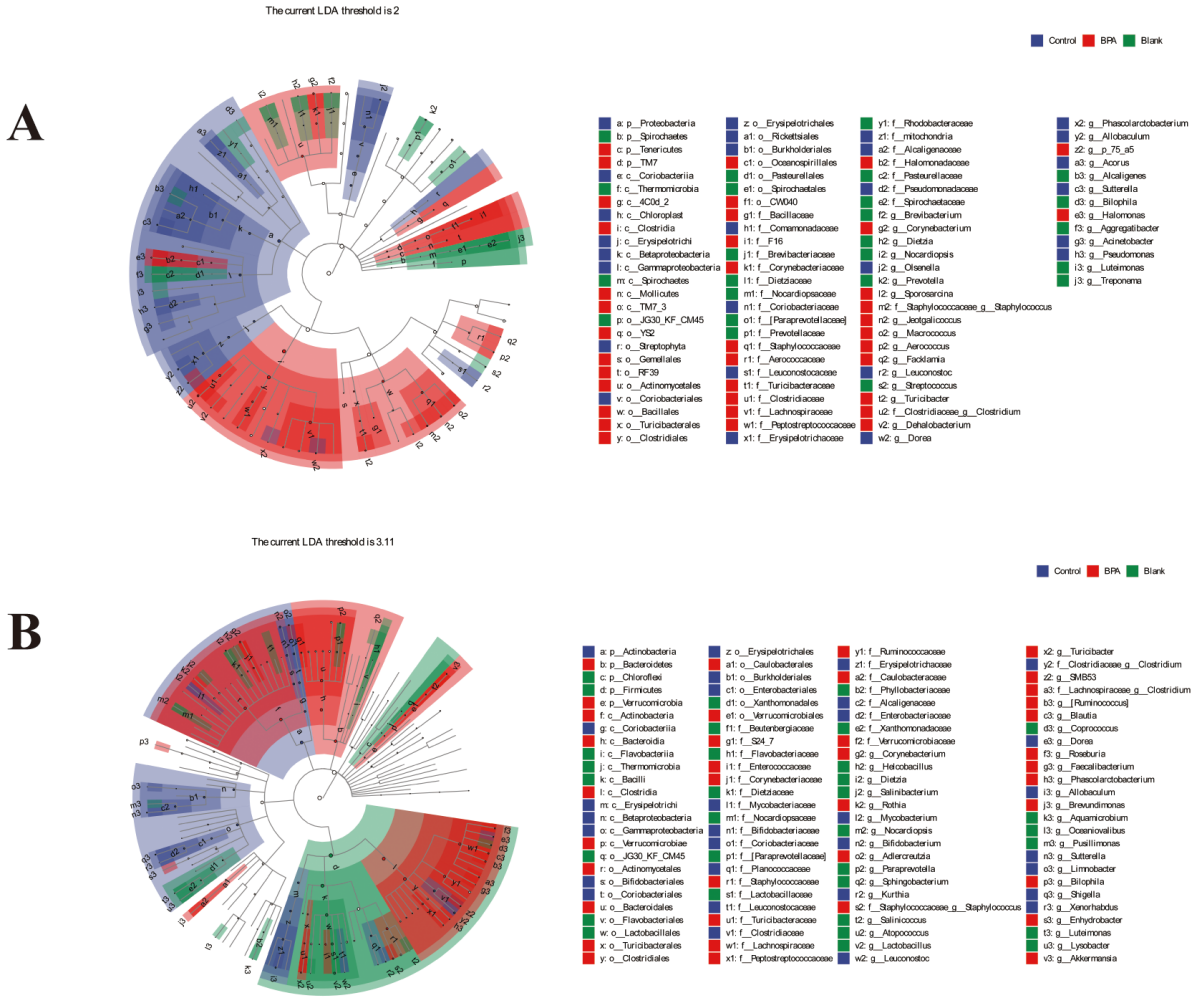
LEfSe analysis of intestinal microbiota after 60-day exposure in rats. **(A)** male groups; **(B)** female groups; n=6.

Compared with the control group, the abundance of *TM7* (*TM7, Saccharibacteria*) and *Tenericutes* increased at the phylum level, and the abundance of *Proteobacteria* decreased in the BPA group (♂, **Figure 8**). In the BPA group (♀), the abundance of *Verrucomicrobia* increased and the abundance of *Cyanobacteria* decreased, compared with the control group (**Figure 8**).

At the class level, the abundance of *4C0d 2*, *Clostridia*, *Mollicutes*, and *TM7 3* increased in the BPA group (♂, **Figure 8**). While the abundance of *Coriobacteriia*, *Erysipelotrichi*, and *Betaproteobacteria* decreased compared with the control group. In the BPA group (♀), the abundance of *Verrucomicrobiae* increased, and the abundance of *Coriobacteriia* and *Chloroplast* decreased compared with the control group (**Figure 8**). Collectively, these results demonstrated substantial alterations in the gut microbiota exposed to BPA.

### 3.4 Analysis of short chain fatty acids

**Figure 9:**
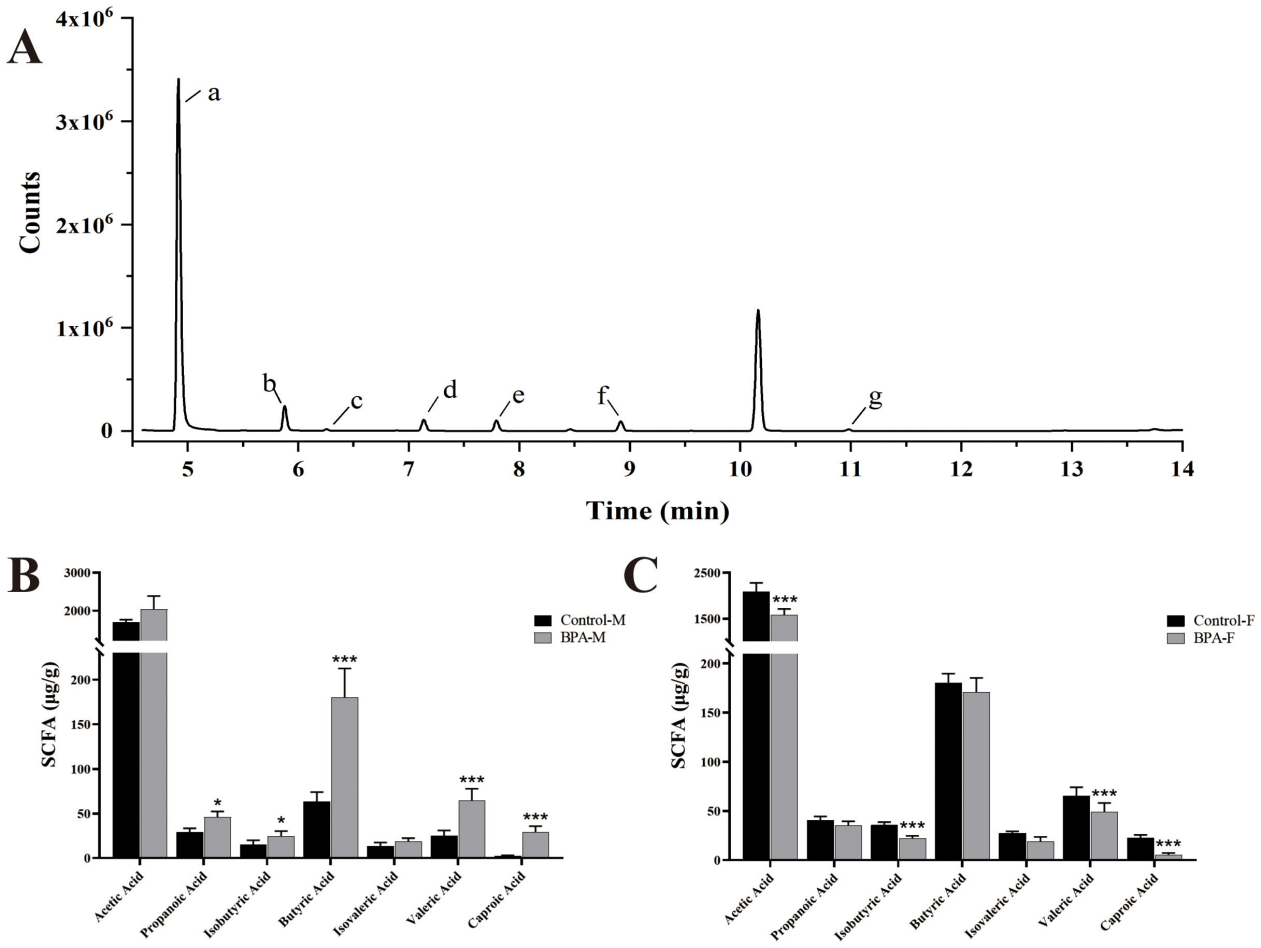
The effect of SCFAs in BPA (300 mg/kg) in rats after 60 days-exposure. Total ion chromatogram (TIC) of 7 SCFAs **(A)**, a: Acetic Acid, b: Propanoic Acid, c: Isobutyric Acid, d: Butyric Acid, e: Isovaleric Acid, f: Valeric Acid, g: Caproic Acid. SCFAs in the male groups **(B)** and female groups **(C)**. n=6; ANOVA followed by Dunnett’s multiple comparison tests; the date is presented mean ± SD. *P < 0.05. and ***P < 0.001 versus the control group.

The contents of SCFAs were quantitatively analyzed by GC-MS (**Figure 9**). The results revealed a significant decrease in the levels of caproic acid, isobutyric acid, butyric acid, and valeric acid, and propanoic acid increased significantly in the BPA group (♂) (P < 0.05). It further showed a significant decrease in the contents of caproic acid isobutyric acid, acetic acid, caproic acid, and valeric acid in the BPA group (♀) (P < 0.05). It can be seen that BPA exposure has different effects on the SCFA levels of different sexes.

### 3.5 Metabolomics analysis

#### 3.5.1 Bile acid profile

**Figure 10:**
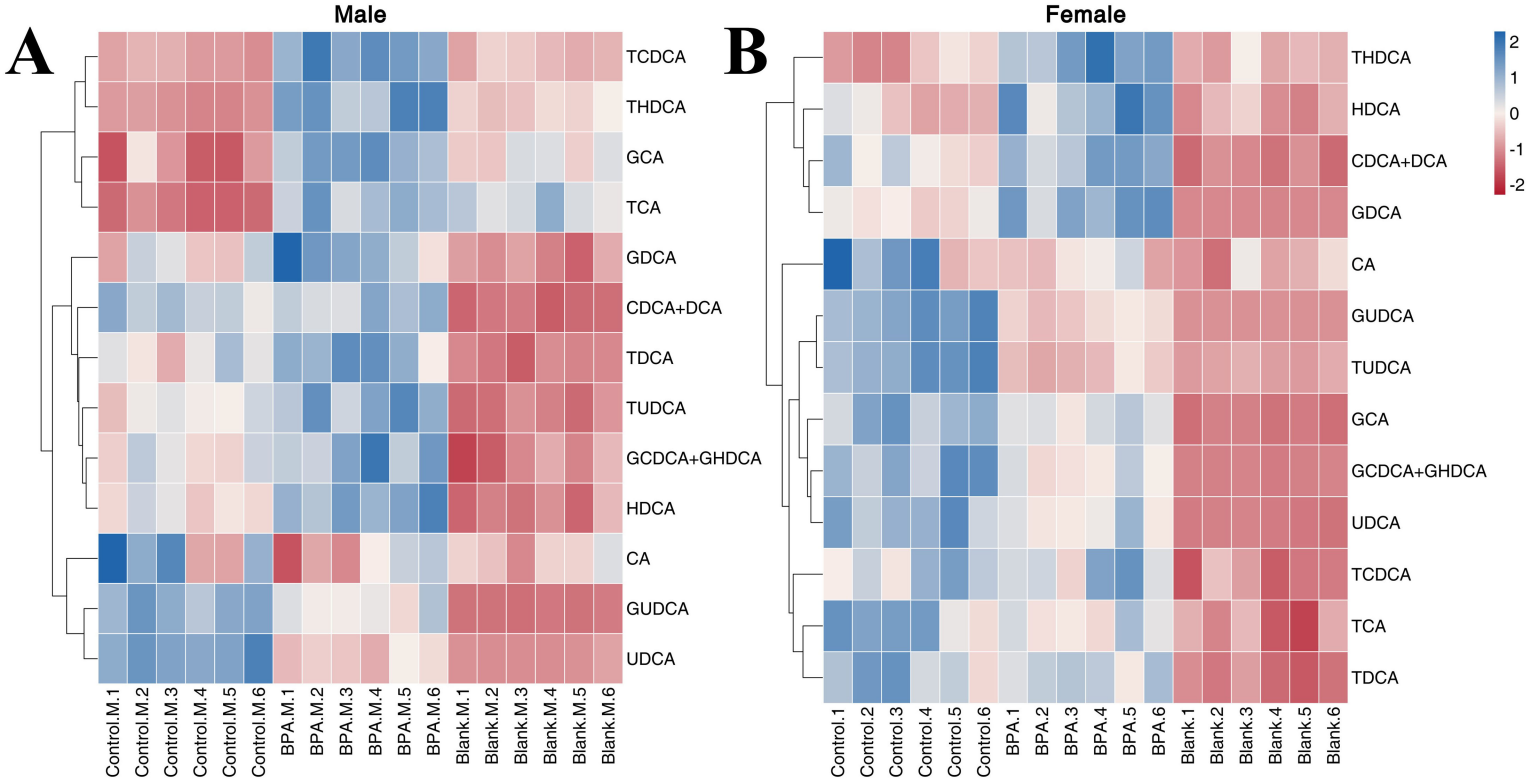
The heatmap of BAs in the serum: male group **(A)** and female group **(B)**. Blue represents the lowest while red represents the highest, hierarchical clustering separates. The X-axis of the heatmap represents different groups within 6 samples, while Y-axis stands for BA levels.

In the BPA group (♂), the levels of GUDCA and UDCA were significantly decreased compared with the control group (P < 0.05), and the levels of HDCA, TCA, TCDCA, TDCA, THDCA, TUDCA, GCDCA+GHDCA, GDCA, and GCA were increased (**Supplementary Figure S7**). In the female group, the levels of CDCA+DCA, HDCA, THDCA, and GDCA were significantly increased compared with the control group (P < 0.05), and the levels of TUDCA, GCDCA+GHDCA, GCA, GUDCA, and UDCA were significantly decreased (P < 0.05). A heatmap shows the relative levels of each bile acid (**Figure 10**). In short, exposure to BPA disrupts the BA metabolism in rats.

#### 3.5.2 Lipid metabolism

**Figure 11:**
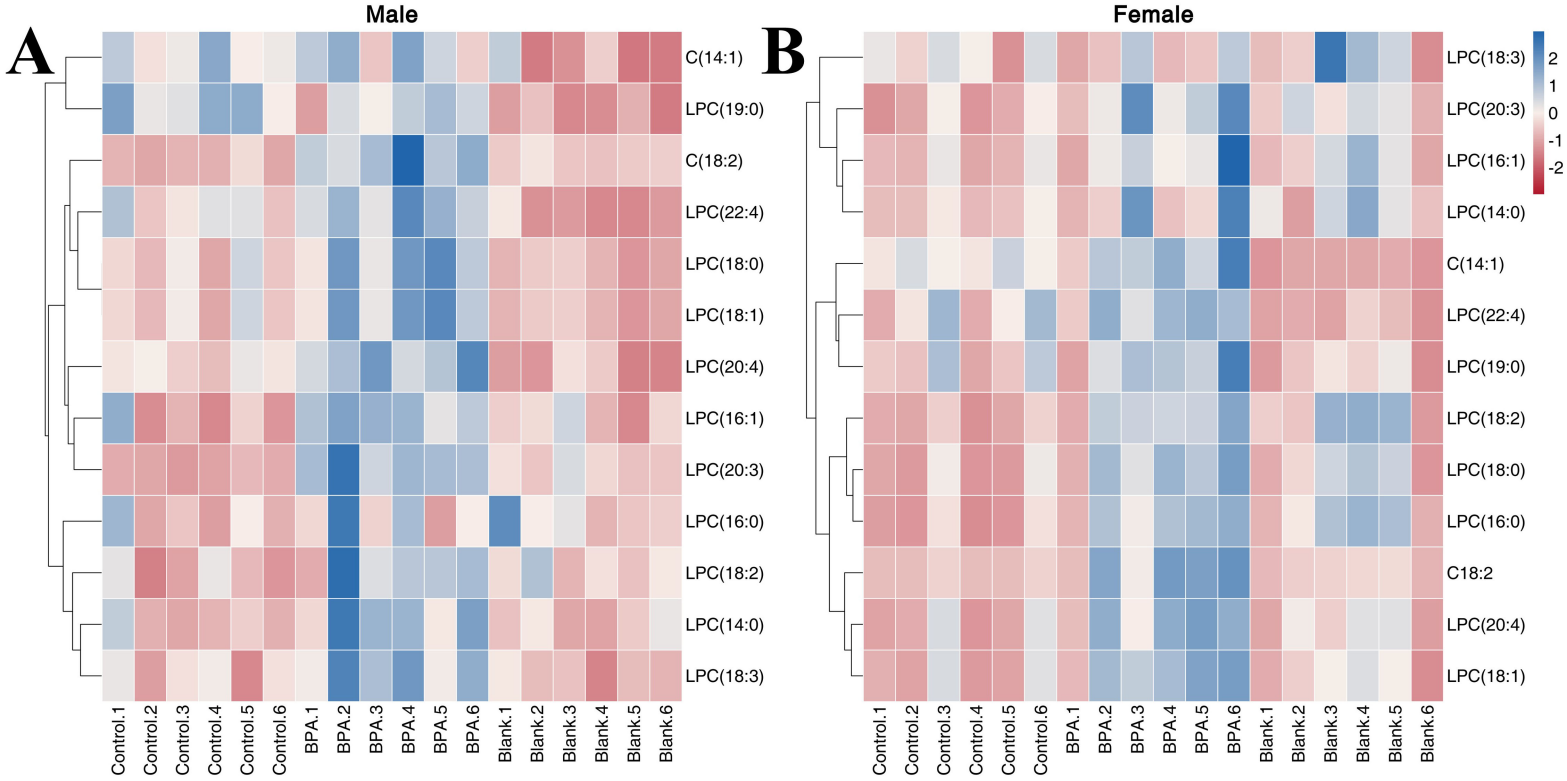
The heatmap of LPCs in the serum. Red represents the lowest while blue represents the highest, the X-axis of the heatmap represents different groups within 6 samples, while the Y-axis stands for LPC levels.

In total, 13 LPCs were determined in order to study the effect of BPA on lipid metabolism in rats (**Supplementary Figure S8**). In the male group, compared with the control group, BPA exposure led to an increase in the contents of C(18:2), LPC(14:0), LPC(16:1), LPC(18:0), LPC(18:2), LPC(18:3), LPC(22:4), LPC(20:4), and LPC(20:3). In the BPA group (♀), the contents of C(18:2), LPC(16:0), LPC(18:0), LPC(18:1), LPC(18:2), LPC(20:3), and LPC(20:4) increased compared with the control group. A heatmap shows the relative levels of each LPC (**Figure 11**). In summary, BPA exposure in rats of different sexes increases the contents of some LPCs.

#### 3.5.3 Amino acid, TCA and oxidative stress metabolism

The contents of 11 amino acids in rats were determined (**Supplementary Figure S9**). In male rats, the contents of Aminohippuric acid, L-Arginine, L-Lysine, L-Ornithine, L-Phenylalanine, L-Proline, L-Threonine, L-Valine, and GABA were significantly decreased after BPA exposure (P < 0.05). In female rats, the contents of Aminohippuric acid, GABA, L-Citrulline, L-Lysine, L-Ornithine, L-Phenylalanine, L-Proline, and L-Threonine were significantly decreased, and the content of L-Arginine was significantly increased (P < 0.05). It can be seen that BPA exposure led to a decreasing trend in amino acid levels.

**Figure 12:**
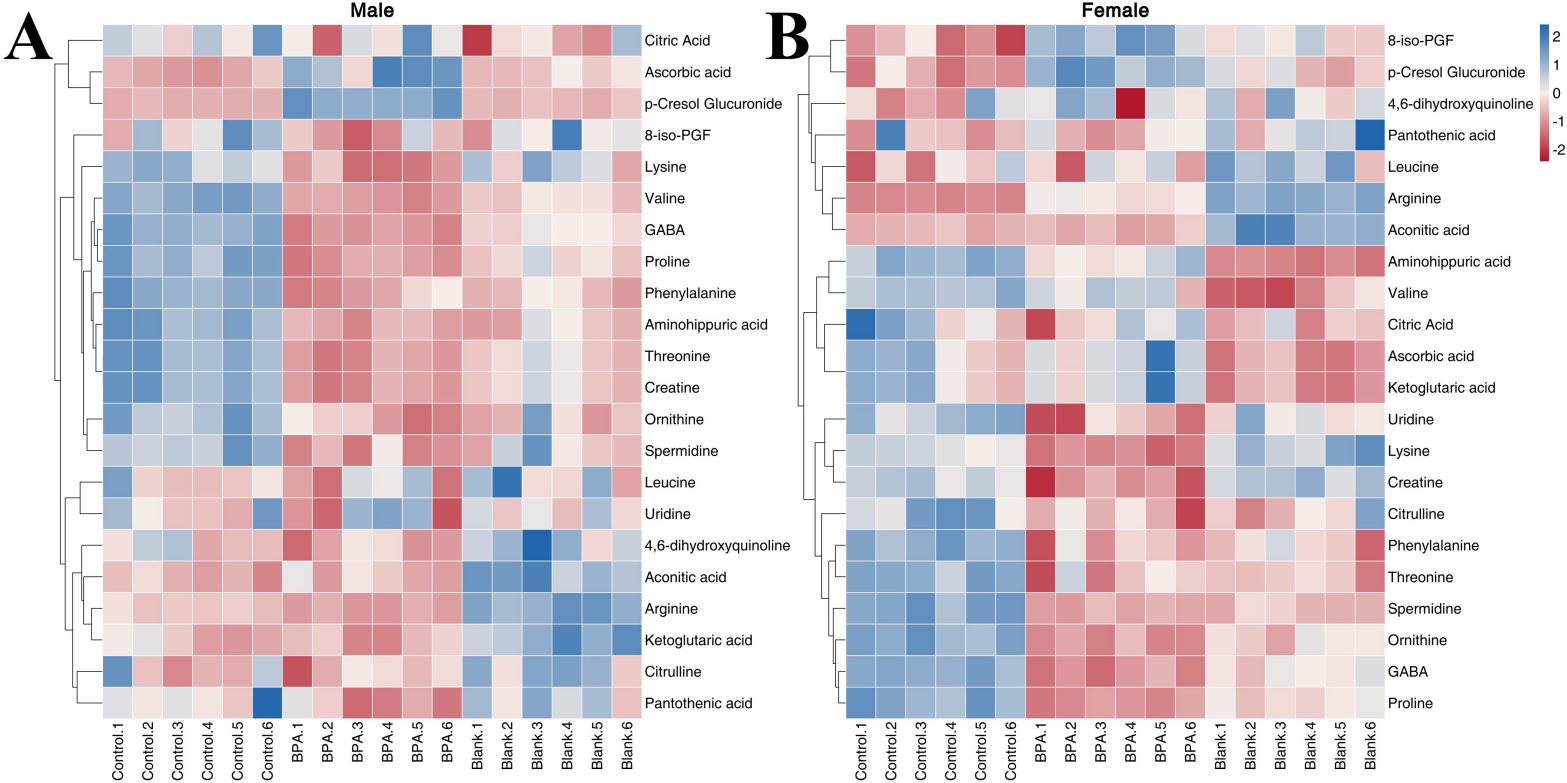
The heatmap of amino acids, TCA, and oxidative stress metabolism: male group **(A)** and female group **(B)**. Blue represents the lowest while red represents the highest. The X-axis of the heatmap represents different groups within 6 samples, while Y-axis stands for metabolite levels.

**Figure 13:**
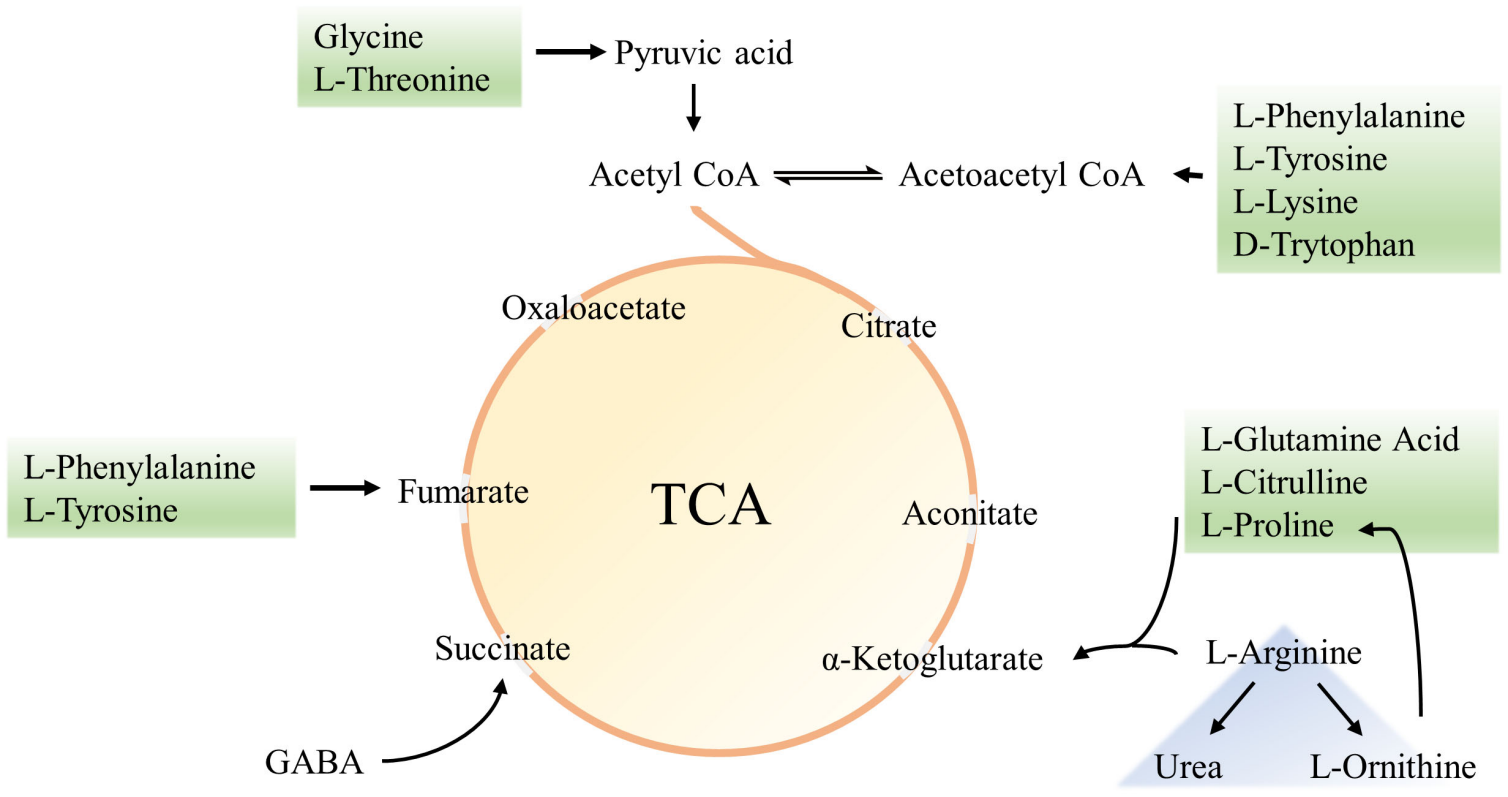
Amino acids and TCA schematic diagram.

In the BPA group (♂) the contents of creatine, pantothenic acid, and spermidine were significantly decreased compared with the control group (P < 0.05), and the contents of ascorbic acid and p-Cresol Glucuronide were significantly increased (P < 0.05). In the BPA group (♀), the contents of 8-iso-PGF and p-Cresol Glucuronide were significantly increased compared with the control group (P < 0.05), and the contents of creatine, spermidine, and uridine were significantly decreased (P < 0.05) (**Supplementary Figure S9**). A heatmap shows the relative levels of each metabolite of amino acids, TCA, and oxidative stress metabolism (**Figure 12**). BPA had an impact on oxidative stress-related metabolites, but it had no significant effect on the level of TCA-related metabolites. As a structural unit of protein, amino acids are important substrates for energy supply in the body and are closely related to the TCA cycle (**Figure 13**), which is the hub of nutrient metabolism in the body. However, after BPA exposure, an increase in amino acid metabolism levels did not significantly affect the TCA cycle.

#### 3.5.4 Steroid hormone metabolism

In recent years, the effect of intestinal flora on the reproductive system has become a new research focus. There is a significant gender difference in the intestinal flora (32), and intestinal flora transplantation can significantly change the levels of various sex hormones (33). Hormones also have an impact on the gut microbiota, with both gonadectomy and hormone replacement therapy causing significant changes in the species composition of the gut microbiota (20).

**Figure 14:**
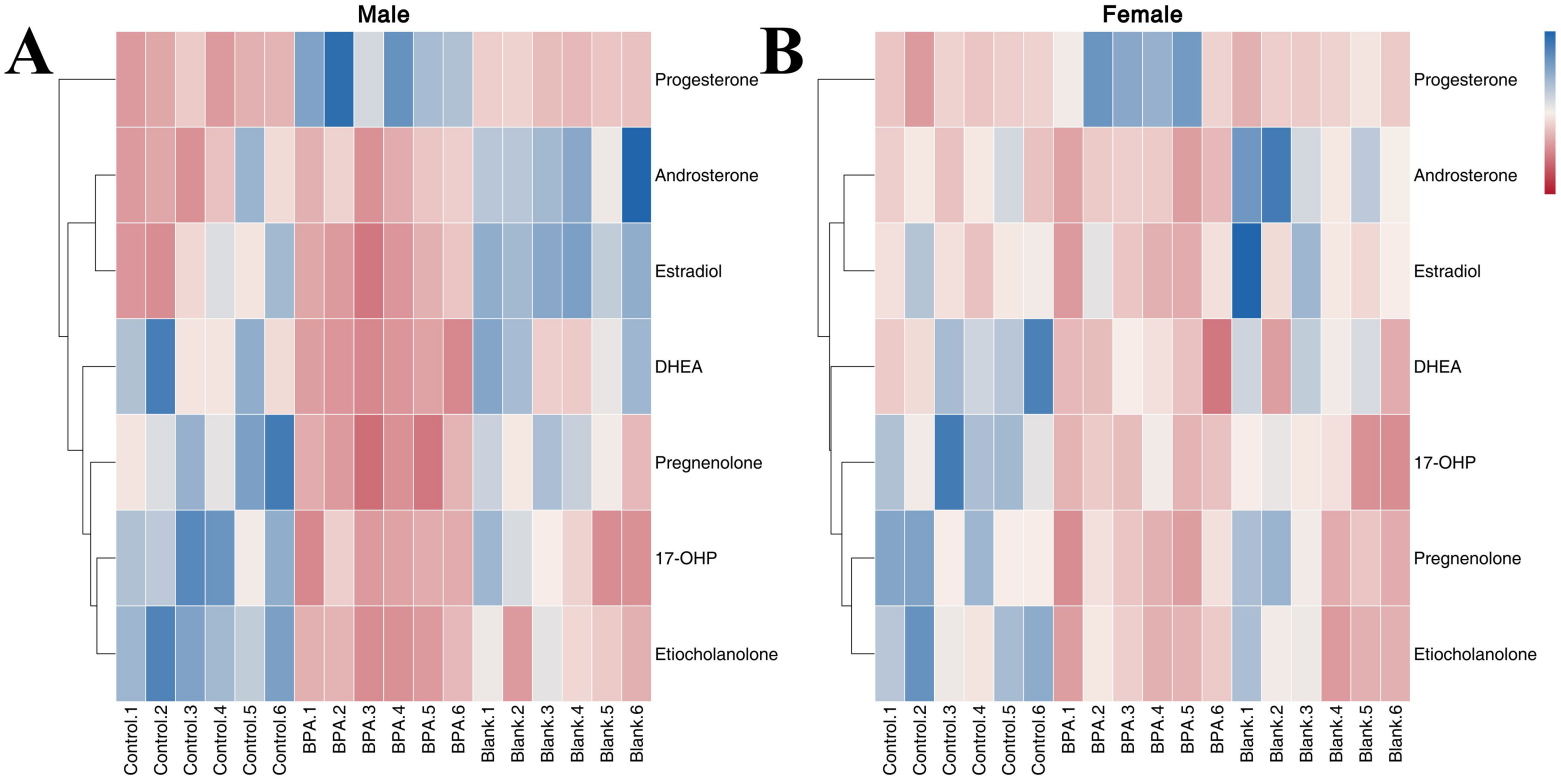
The heatmap of steroid hormone metabolism in male groups **(A)** and female groups **(B)**. Blue represents the lowest while red represents the highest. The X-axis of the heatmap represents different groups within 6 samples, while the Y-axis stands for metabolite levels.

The contents of seven hormones in the serum of rats were analyzed to study the effect of BPA exposure on sex hormone levels (**Supplementary Figure S10**). Compared with the control group and blank group, the contents of etiocholanolone, 17-OHP, pregnenolone, and DHEA in the BPA group (♂) were decreased significantly (P < 0.05), and the content of progesterone was increased significantly. In the BPA group (♀), the contents of 17-OHP, etiocholanolone, DHEA, and pregnenolone were significantly decreased (P < 0.05), and the content of progesterone was significantly increased (P < 0.05). A heatmap shows the relative levels of hormones (**Figure 14**). In summary, BPA exposure regulated the hormone levels in rats, and it is worth noting the significant increase in progesterone.

### 3.6 Analysis of inflammatory factors

**Figure 15:**
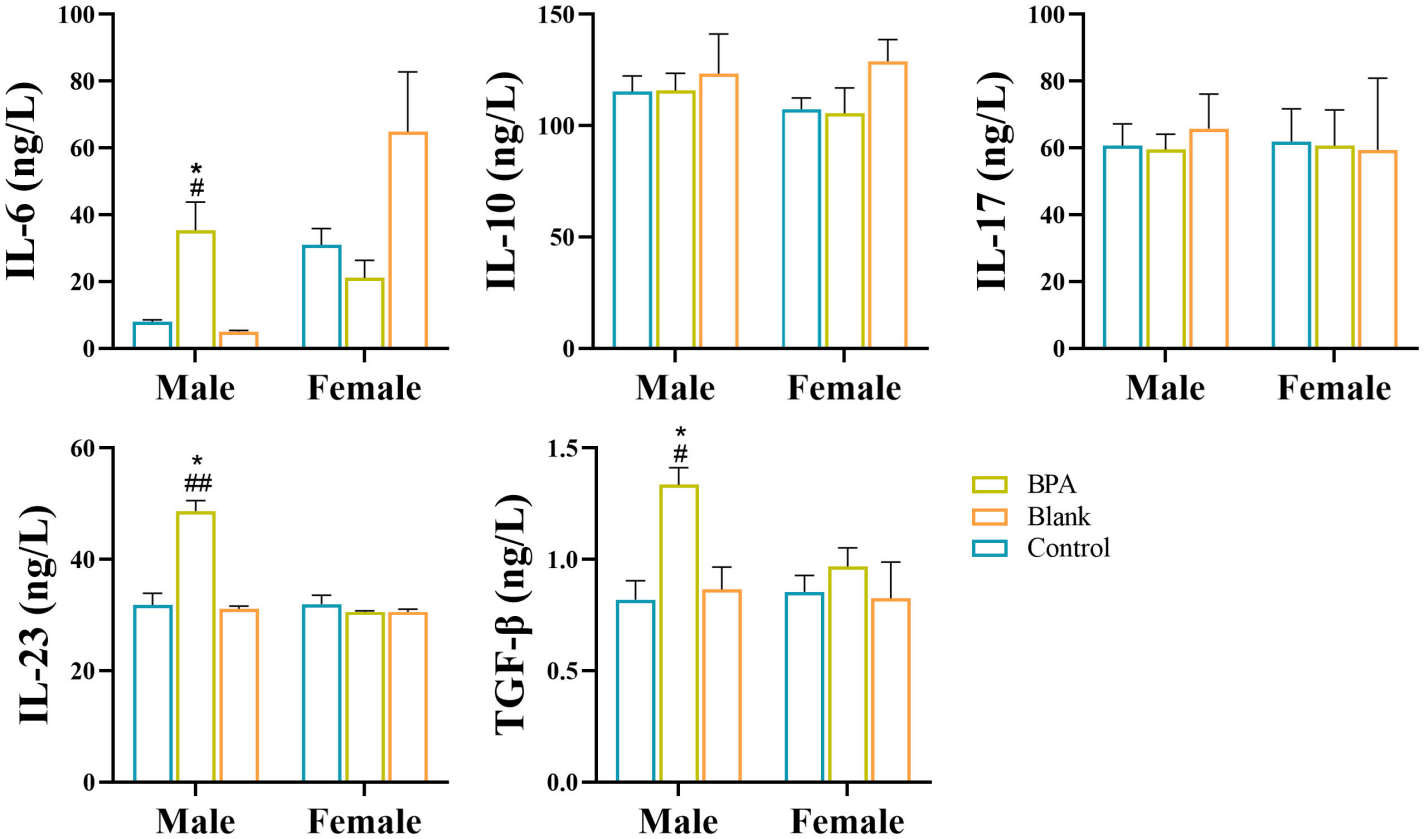
Analysis of inflammatory factors by ELISA. #P < 0.05, ##P < 0.01 *vs.* Blank group; *P < 0.05 *vs.* Control group. n=6; ANOVA followed by Dunnett’s multiple comparison tests; the data is presented as mean ± SD.

Compared with the control group, the contents of IL-6, IL-23, and TGF-β in the BPA group (♂) were increased (P < 0.05) as shown in **Figure 15**. BPA had no significant effect on inflammatory factors in the female group. Th17 cells are a prominent subset of CD4^+^ T cells and play a crucial role in promoting inflammatory immune responses. Their differentiation is dependent on specific polarized cytokines such as TGF-β, IL-6, and IL-23 (34). The increase in IL-6, IL-23, and TGF-β, suggests that BPA exposure affected the autoimmune inflammatory response in the BPA group (♂).

There were statistically significant differences in the levels of metabolites, biochemical indexes, and inflammatory factors (P < 0.05) between the BPA group (♂ or ♀) and the control group (♂ or ♀), suggesting that BPA exposure had a significant impact on the gut-liver-hormone axis.

## 4 Discussion

BPA, a traditional endocrine-disrupting chemical, exhibits estrogen-like effects and has been linked to various diseases including obesity and reproductive disorders (35–37). However, a comprehensive understanding of its toxicity mechanism is still lacking, particularly in relation to its impact on the liver-gut-hormone axis.

The results of biochemical indicators showed that BPA exposure affected the contents of biochemical indicators. TP is an important indicator of liver function, reflecting the reserve capacity of the liver, which is the sum of ALB and GLOB (38). When exposed to BPA, the content of TP increased in male and female groups, and there was also an increase in ALB and GLOB levels. However, the ratio of ALB to GLOB (A/G) decreased, indicating that the increase in GLOB was higher than that of ALB.

The liver plays an important role in the metabolism of bilirubin (BIL), including the uptake, binding, and excretion of unconjugated BIL in the blood by hepatocytes (39). TBIL is the combined amount of DBIL and IBIL (40). Obstacles in the processes of uptake, binding, and excretion can cause an accumulation of BIL in the blood and increase the content of BIL. In the BPA group, the increase in TBIL was mainly caused by an increase in DBIL. DBIL is the conversion of IBIL in hepatocytes, which is combined with glucuronic acid. An increase in DBIL indicates that the excretion of BIL from the bile duct is blocked after toxicity exposure. The increase in TBIL in the female rats was mainly due to an increase in IBIL. IBIL is a form of bilirubin that does not bind to glucuronic acid, making it insoluble in water and unable to be excreted through the kidneys (41). High levels of IBIL are produced when a significant number of red blood cells are broken down in the body. Liver damage can impair the metabolism of normal BIL, leading to an accumulation of IBIL in the blood. The changes in biochemical indicators suggest that BPA may have an effect on the liver.

The Chao1 index in the BPA group (♂) decreased significantly (P < 0.05). This indicated that exposure to BPA reduced the species diversity and changed the structure of gut flora. The decrease in Faith’s PD index indicated that under the influence of BPA, the evolutionary difference of rat intestinal flora became smaller. The effect of BPA on male rats was higher than that on female rats.

*Firmicutes* and *Bacteroidetes* are the most important bacteria in the body, and their relative abundance of them accounts for more than 80% of the species (42). *Firmicutes* mainly produce butyric acid, and *Bacteroidetes* mainly produce acetic acid and propionic acid (43, 44). The ratio of *Firmicutes/Bacteroidetes* (F/B) is an important indicator for evaluating gut microbes (45). In both the male and female groups, dysbacteriosis was observed following BPA intake, as evidenced by changes in the F/B ratio. Previous studies have established a strong correlation between an increased F/B ratio and fat deposition as well as metabolic disorders. Conversely, a decreased F/B ratio has been associated with reduced levels of SCFAs, accumulation of lipopolysaccharide, and the induction of an immune inflammatory response (46, 47).

SCFAs are the end products of intestinal flora. SCFAs participate in the maintenance of intestinal mucosa integrity, improve glucose and lipid metabolism, control energy expenditure, and regulate the immune system and inflammatory responses (48). The bacteria responsible for producing SCFAs in the body primarily include *Anaerobic Bacteroides*, *Bifidobacteria*, *Eubacteria*, *Streptococcus*, and *Lactobacillus* (49). Experimental results indicate that the intake of BPA can lead to changes in SCFA-producing bacteria and subsequently affect SCFA levels. The impact of BPA on SCFAs varied between the male and female rats. In the male group, SCFA levels increased to varying degrees, while in the female group, they decreased to varying degrees. *Clostridiaceae Clostridium*, a major group of bacteria producing different SCFAs in the intestine, exhibited a changing trend similar to SCFAs (50). This sex difference may be attributed to inherent variations in hormone levels between sexes, potentially mediated through a reciprocal relationship between sex hormones and gut flora, influencing changes in levels. There were also sex differences in hormone level results. Functional differences in murine estrogen receptor β status have been reported between representative orders (e.g., *Lactobacillus*) and specific phyla (e.g., *Proteobacteria, Bacteroidetes*, and *Firmicutes*), suggesting that nuclear steroid receptor status and nutritional complexity may play important roles in maintaining the microbiome (51). α-diversity has a negative correlation with estradiol concentrations, but the mechanism remains unclear (52). Etiocholanolone is a metabolic product of testosterone and androstanolone, which decreases with androstanolone level. Dehydroepiandrosterone is an important precursor of androgens and estrogens, a decrease in it directly affects the subsequent biosynthesis of estrogens and androgens. Sex hormones are mainly metabolized and degraded in the liver, and some hormones are transported into the intestine, which is affected by the metabolites of intestinal flora (53). The abundance of *Clostridium* in the family *Clostridiaceae* was correlated with a change in estrogen (54). The abundance of *Clostridiaceae* increased in the BPA group (♂), but decreased in the BPA group (♀). BPA could affect the hormone contents in rats by affecting the levels of intestinal flora.

UDCA is a secondary bile acid produced by intestinal bacteria after conversion of CDCA. It is a key factor in the integrity of the intestinal barrier and has an important influence on lipid metabolism (55). After BPA exposure, its levels were significantly reduced compared to that of control group, indicating that it may have an impact on intestinal and lipid metabolism. In male rats, except for GUDCA, the levels of other conjugated bile acids were increased, and together with the transaminase results, it can be concluded that toxic exposure led to an increase in the levels of upstream substances such as cholesterol and bilirubin, while promoting the release of free bile acids.

BAs are produced from cholesterol in hepatocytes. Primary BAs, including CA and CDCA, are essential for the digestion and absorption of lipids and vitamins (56) and 90% of them are actively reabsorbed by the terminal ileum and recirculated in the liver (enterohepatic circulation). Primary BAs are also converted into secondary BAs and deconjugated into unconjugated BAs by intestinal microbiota (57), which can be passively reabsorbed into the BA pool or excreted through feces. The contents of conjugated BAs in the rats were significantly increased, which indicated that the intake of BPA interferes with the conversion of conjugated BAs into unconjugated bile acid via intestinal microbiota.

It is well-recognized that gut microbiota influences host lipid metabolism (58). A high-fat diet (HFD) in mice has been found to promote the development of colorectal cancer by causing intestinal microbial disorders which led to an increase in lysophosphatidic acid (59). After exposure to BPA, there was a significant alteration in the levels of LPCs in the rats, suggesting that BPA intake can disrupt lipid balance via intestinal microbiota, increasing the risk of high fat and obesity.

Amino acids interact with intestinal flora, participate in microbial metabolism, and play a role in maintaining the balance of the intestinal environment (64). It is produced during the fermentation of intestinal flora, proteins, and bioactive peptides (60). Studies have shown that in the treatment of hyperlipidemia, the positive regulation of drugs on intestinal flora can increase the content of citrulline and ornithine (61). The results of this study showed that toxic exposure reduced the content of some amino acids by affecting the intestinal flora.

The results showed that the expression of the pro-inflammatory factors IL-6, IL-23, and the anti-inflammatory factor TGF-β increased in the serum of male rats, which indicates an inflammatory process. GLOB is produced by the immune organs and its increase indicates inflammation or invasion by exogenous substances. Excessive production of GLOB by the immune system results in a low A/G ratio (62). Research has shown that T cells can regulate the synthesis and metabolism of BA through inflammatory factors (63). It can be seen that BPA exposure has an effect on the hepatic immune system and may be involved in the T cell-mediated immune response.

## 5 Conclusions

This study reported the subchronic effects of BPA on the gut-liver-hormone axis in rats of different sexes for the first time. There was pathological damage in the heart, liver, and testis tissues of rats exposed to BPA and it further affected the contents of biochemical indicators. BPA exposure reduced the diversity of intestinal flora and reduced the species category of flora. The metabolomic study showed that the contents of bile acids, short-chain fatty acids, amino acids, and other metabolites were also affected. The detection results of inflammatory factors also suggested that BPA exposure affects the hepatic immune system. The toxicity of BPA based on the gut-liver-hormone axis was evaluated systematically, which holds significant theoretical significance for understanding its toxic mechanism. Additionally, there are valuable research implications for studying the environmental intake, mechanism of action, and health risk level associated with BPA. Furthermore, this research provides important guidance for investigating the toxicological effects of environmental pollutants and for developing strategies to treat and improve water environments.

## Data Availability

The datasets presented in this study can be found in online repositories. The names of the repository/repositories and accession number(s) can be found in the article/**Supplementary Material**.
