# Supplementary material for: Subchronic toxic effects of bisphenol A on the gut-liver-hormone axis in rats via intestinal flora and metabolism
Source: Front Endocrinol (Lausanne). 2024 Aug 29;15:1415216. doi: 10.3389/fendo.2024.1415216 (PMC11390593; doi:10.3389/fendo.2024.1415216)
Supplement: Supplementary file 1 [file DataSheet1.docx]

Supplementary Material

**·****Toxic effects of Bisphenol A on gut-liver-hormone axis in rats via intestinal flora and metabolism**

Jiaqi Wang^1, 3†^, Ce Su^4†^, Mingqin Qian^5^, Xin Wang^1, 3^, Changlan Chen^1^, Yangcheng Liu^1, 3^, Wei Liu^1^, Zheng Xiang^1, 2^*, Baoli Xu^2^*

^1^ School of Pharmaceutical Science, Liaoning University, Shenyang, China

^2^ Affiliated Zhongshan hospital of Dalian University, Dalian, China

^3^ Shenyang Key Laboratory for Causes and Drug Discovery of Chronic Diseases, Shenyang, China

^4^ Pharmacy Department, Shenyang Tenth People’s Hospital, Shenyang, China

^5^ Departments of Ultrasound, People’s Hospital of Liaoning Province, Shenyang, China

The first two authors, Wang and Yu, contribute equally to this research.

*** Correspondence:**Zheng Xiang*
[rainbowaftersnow@hotmail.com](mailto:rainbowaftersnow@hotmail.com)

Baoli Xu*

[xubaoli1981@hotmail.com](mailto:xubaoli1981@hotmail.com)

**
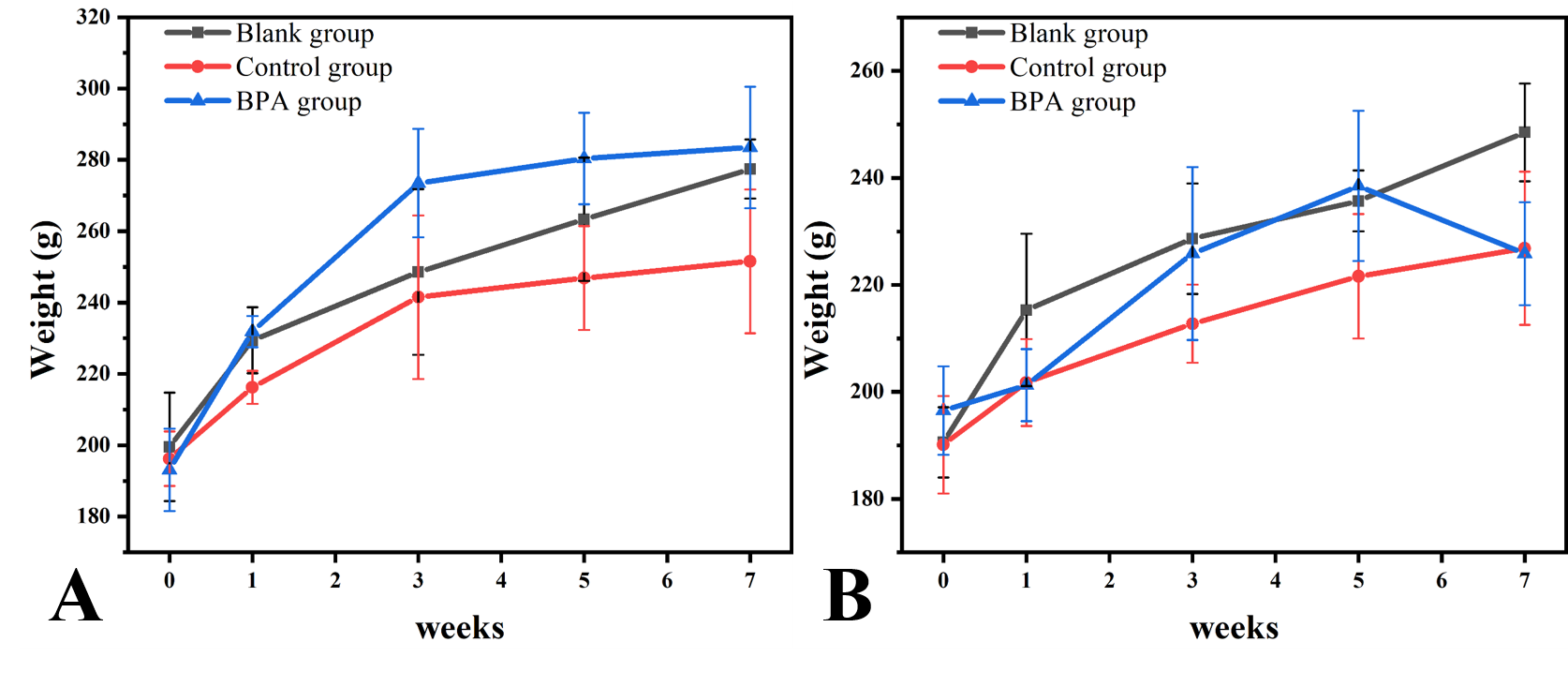
**

## **Fig. S1.** Body weight progression of male and female rats. (A: male, B: female, Mean ± SD, n=6). The difference between the final body weight and the initial body weight was analyzed through ANOVA, and the results showed that the effect of BPA intake on body weight was not significant.


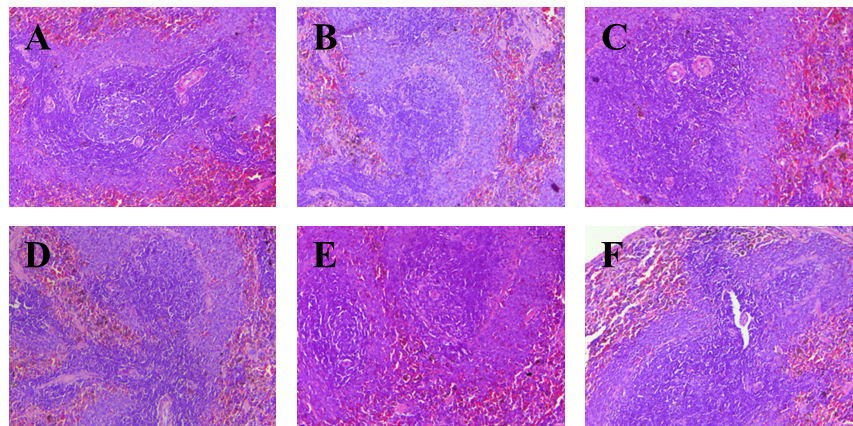


## **Fig. S2.** HE stains of the spleen (200x), A: male-blank group, B: male-BPA group, C: male-control group, D: female-blank group, E: female-BPA group, F: female-control group.


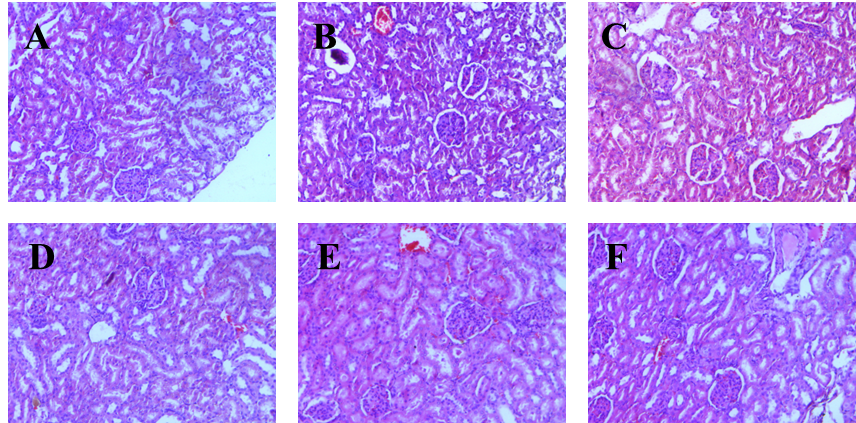


## **Fig. S3.** HE stains of the kidney (200x), A: male-blank group, B: male-BPA group, C: male-control group, D: female-blank group, E: female-BPA group, F: female-control group.


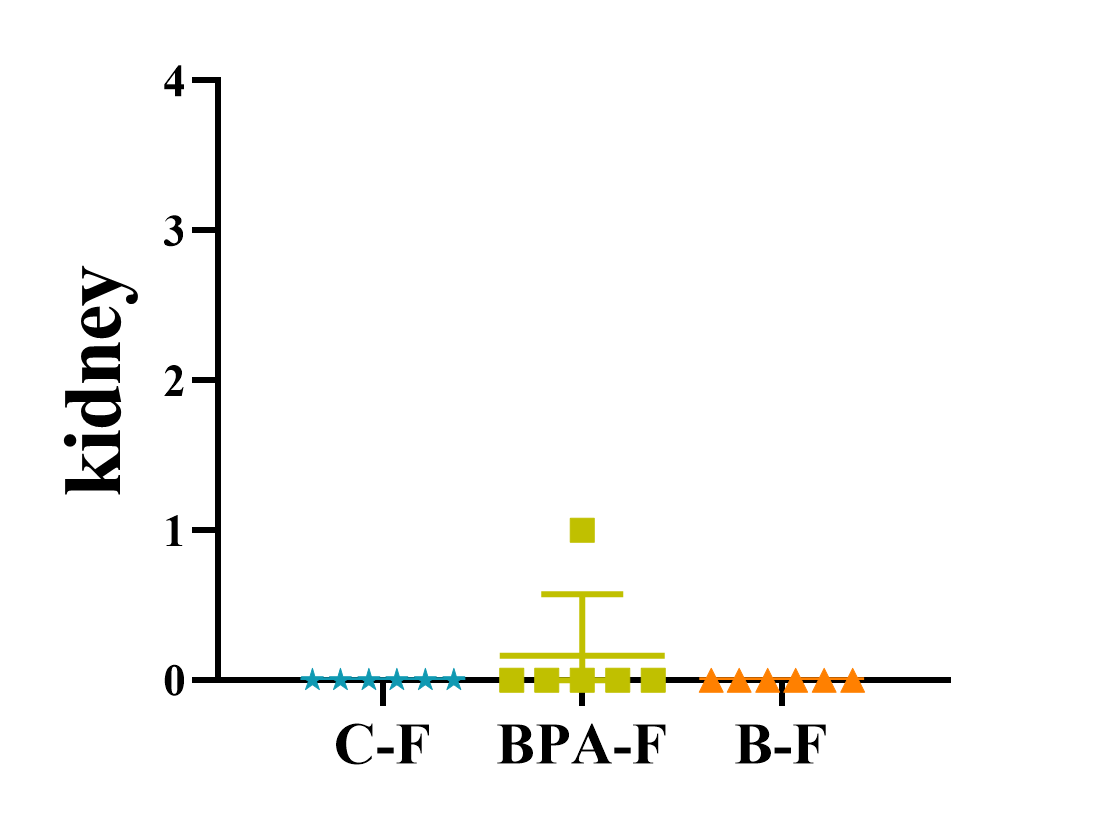

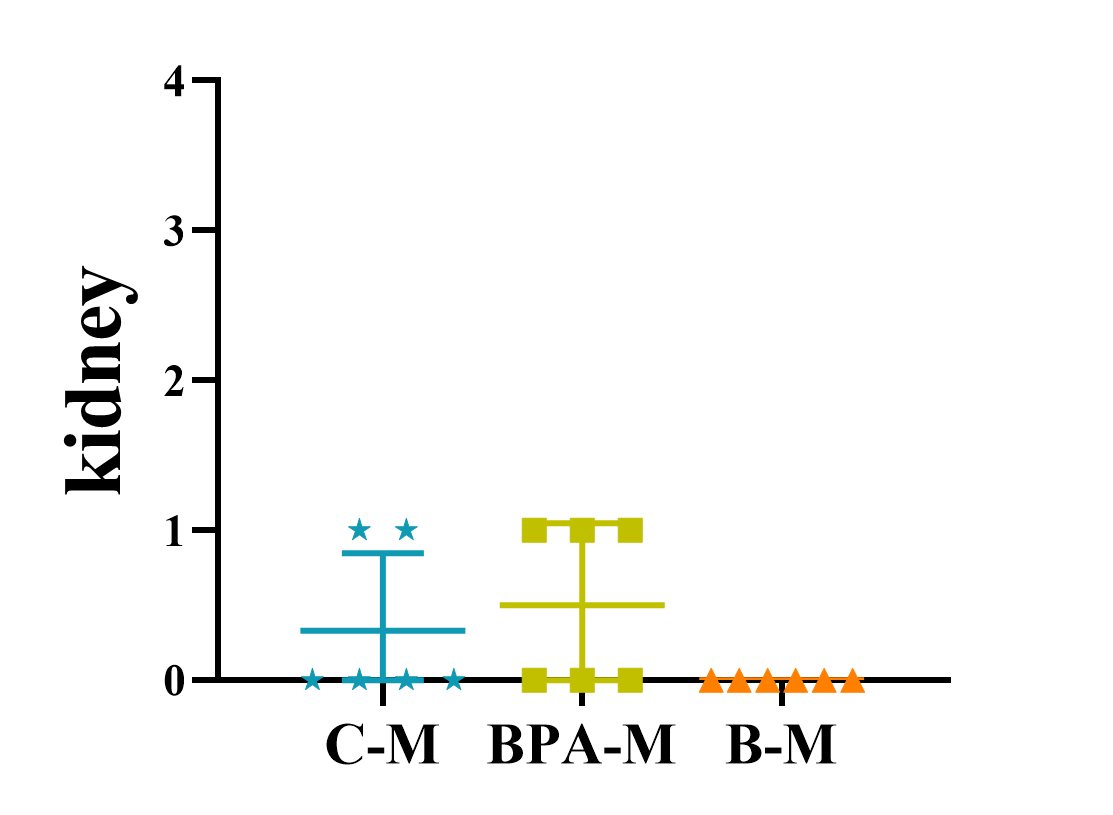

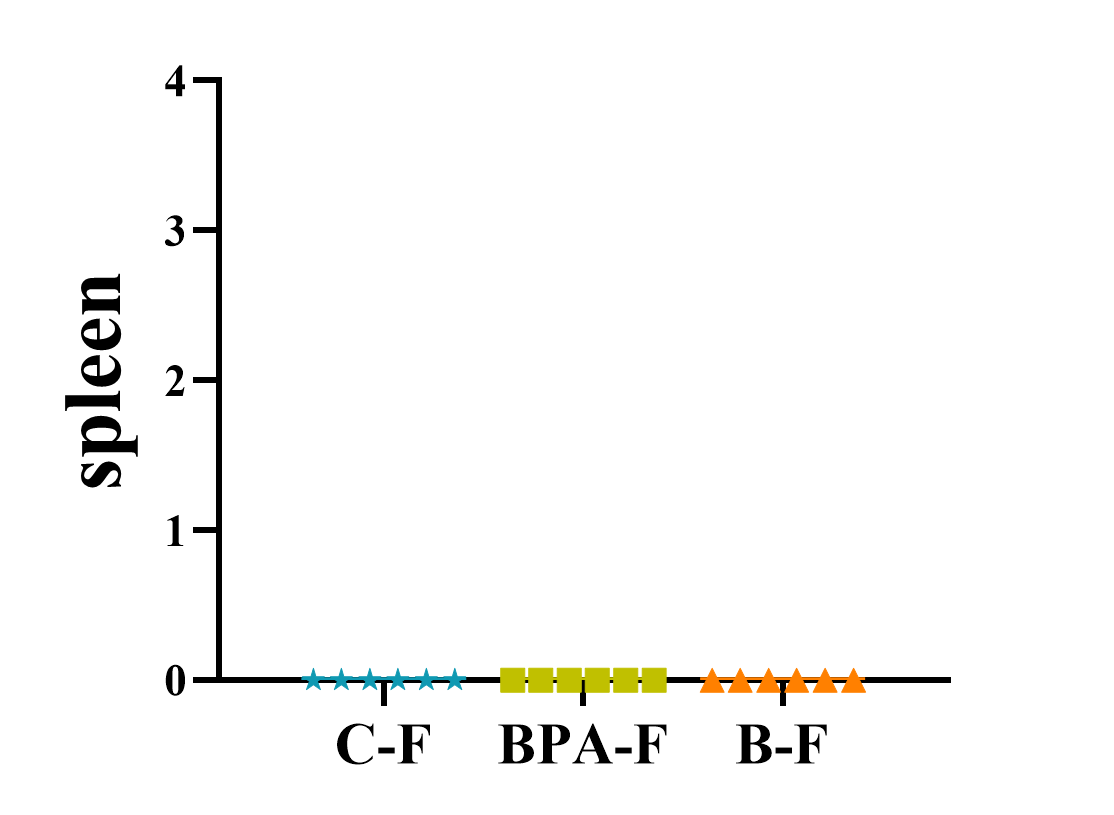

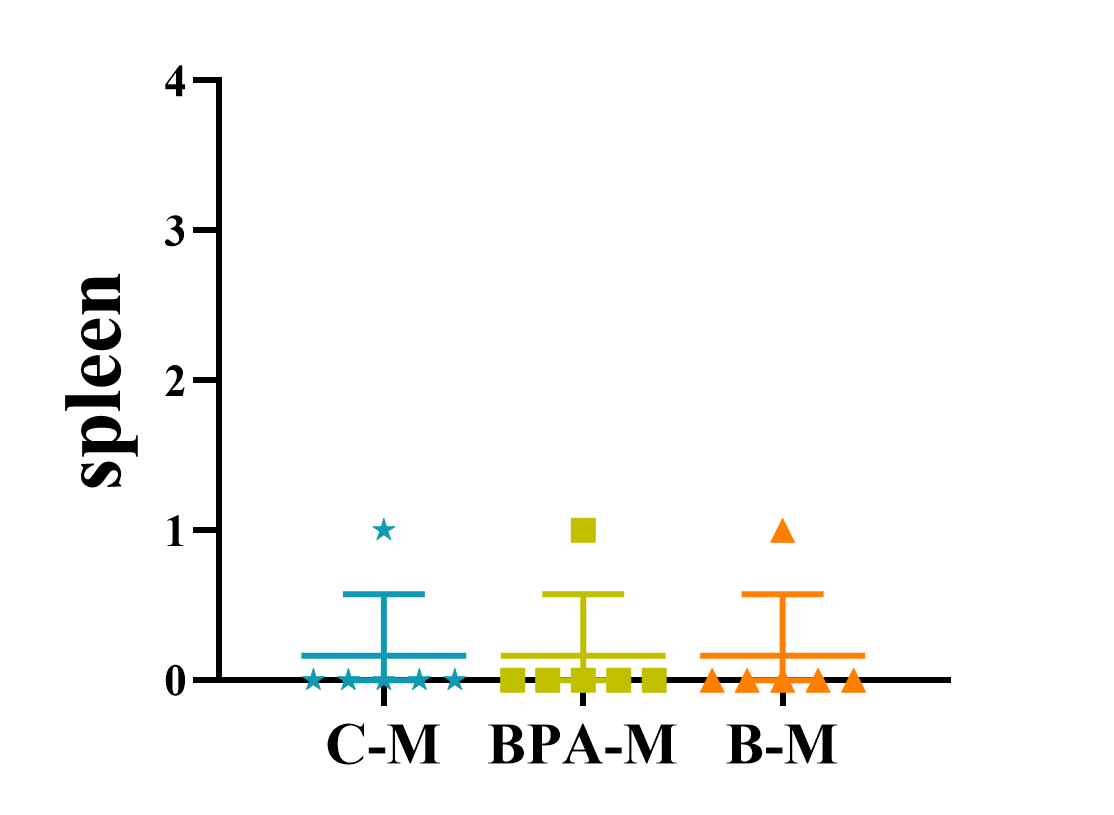


## **Fig. S4.** The pathological score of the kidneys and spleens in control group, BPA group (300 mg/kg) and blank group. Data are mean ± SD. (n = 6), marking criterion: 0, normal; 1, mild; 2, moderate; 3, severe.


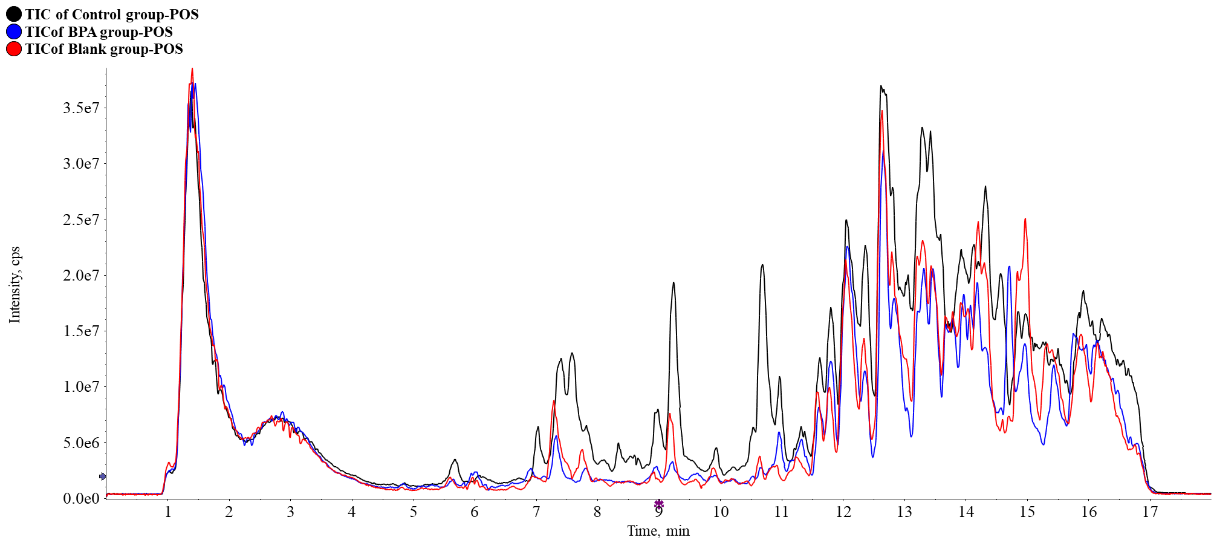


## **Fig. S5.** The total ion chromatogram of metabolites in positive ion mode.


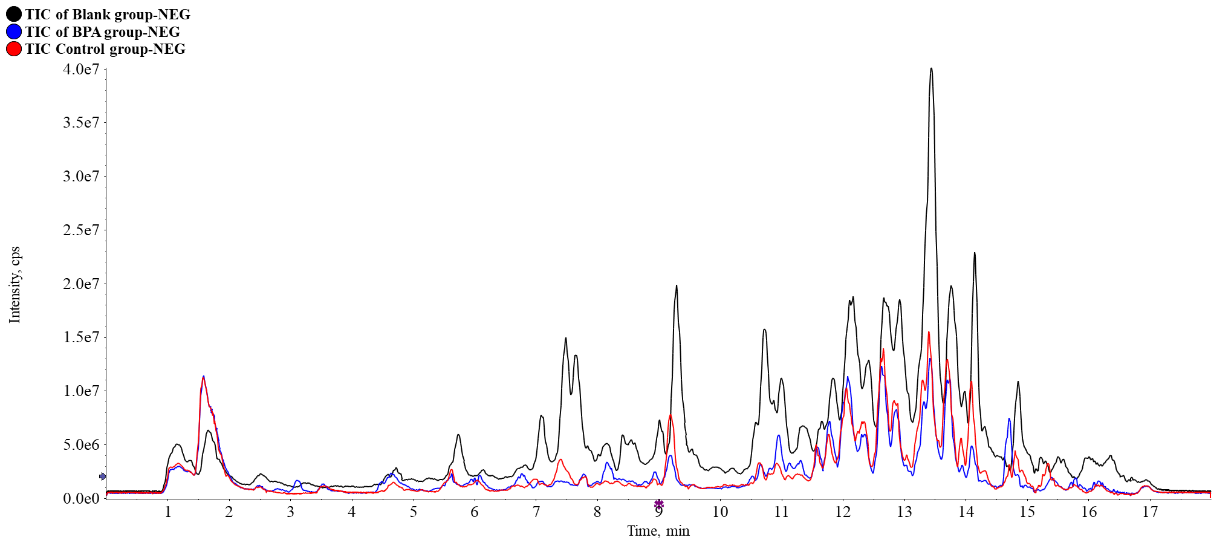


## **Fig. S6.** The total ion chromatogram of metabolites in negative ion mode.

**
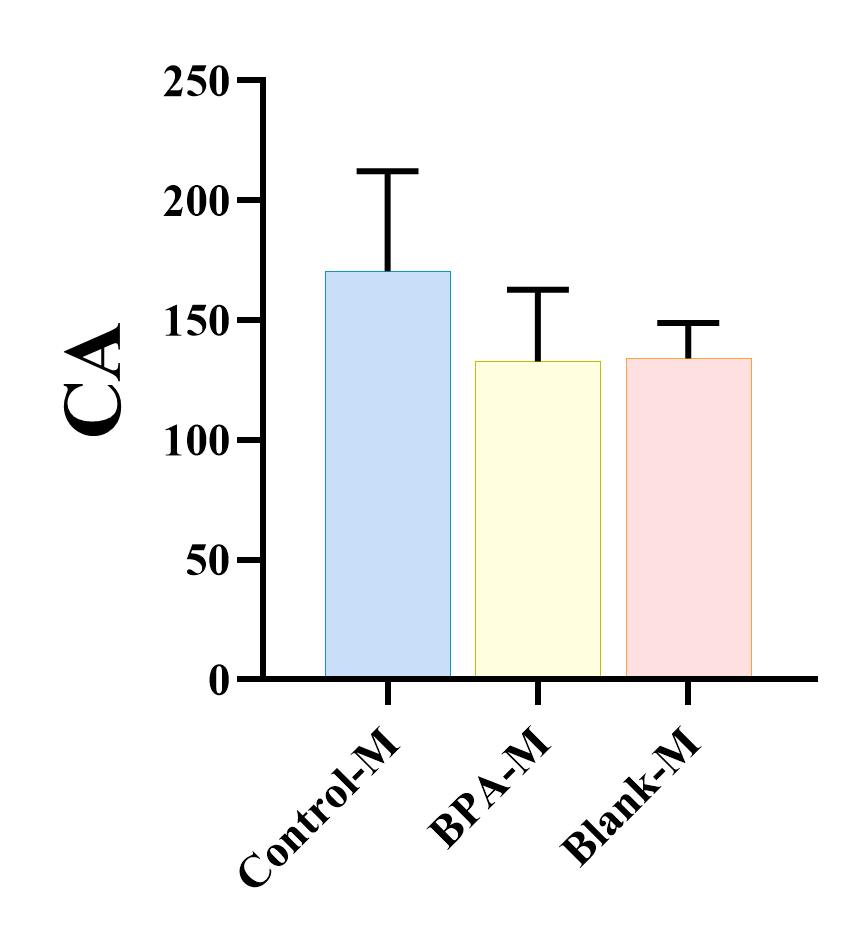

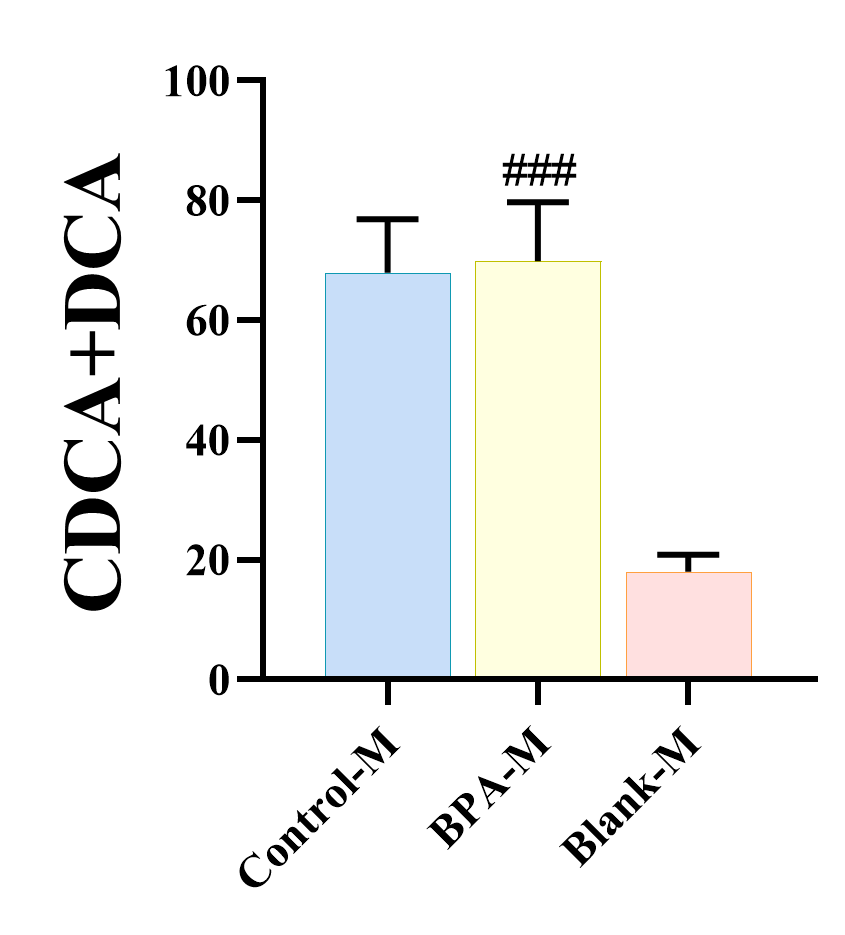
**
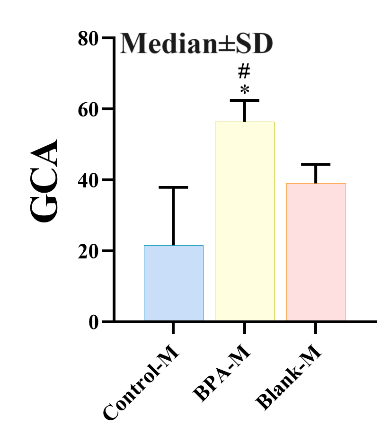


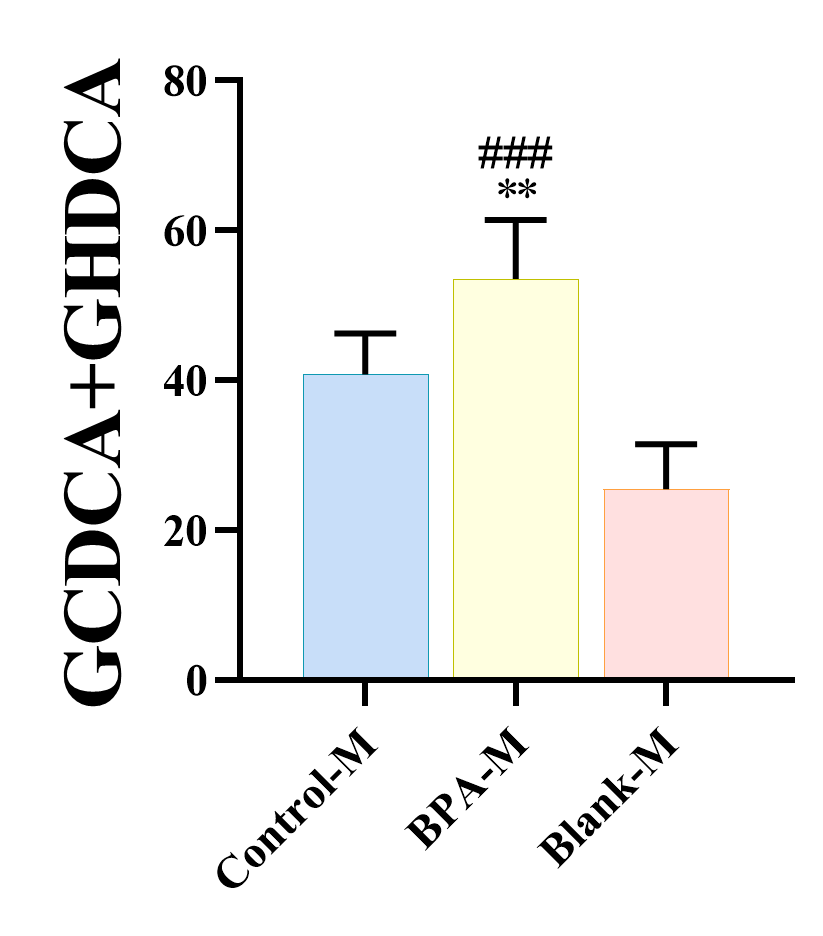

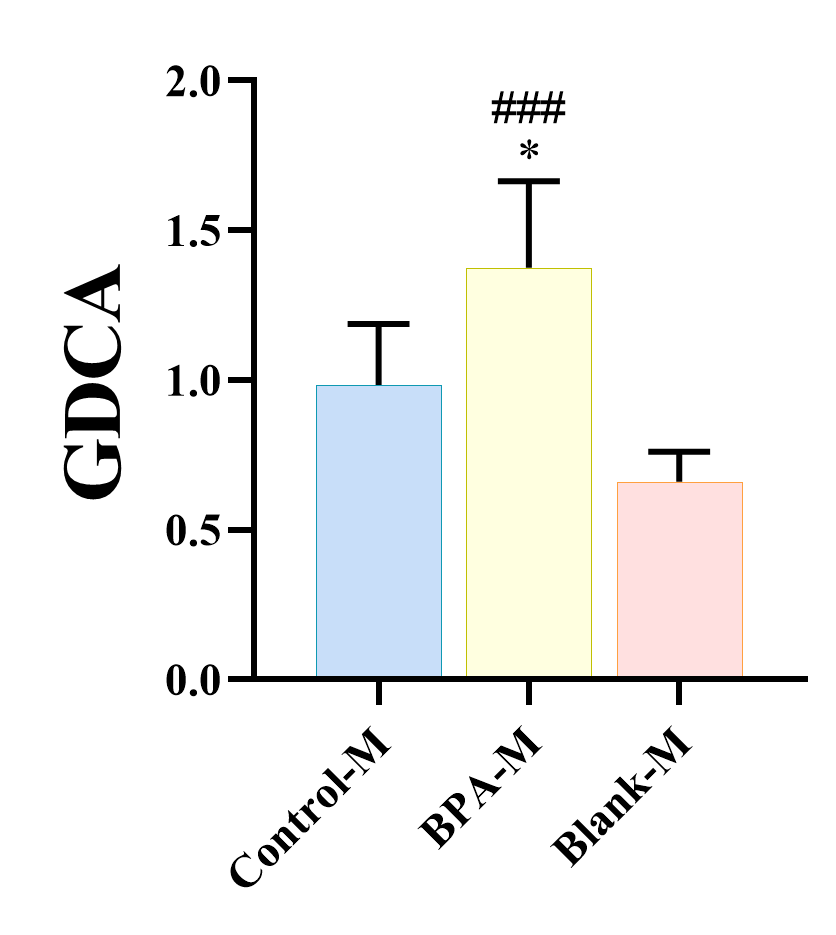

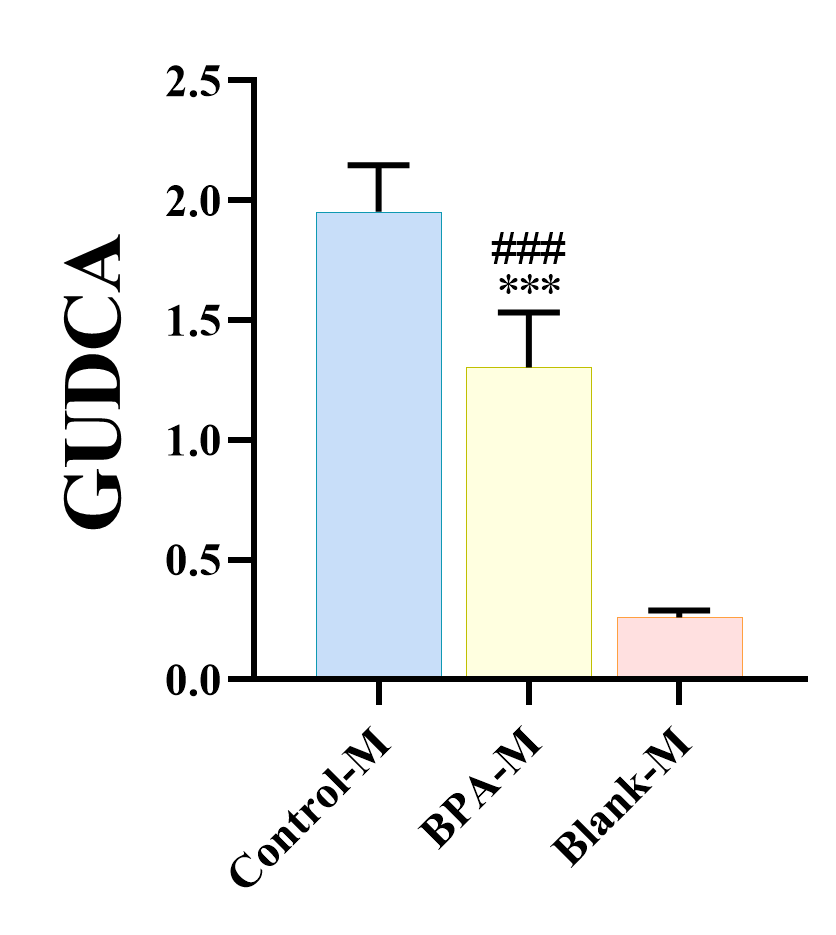

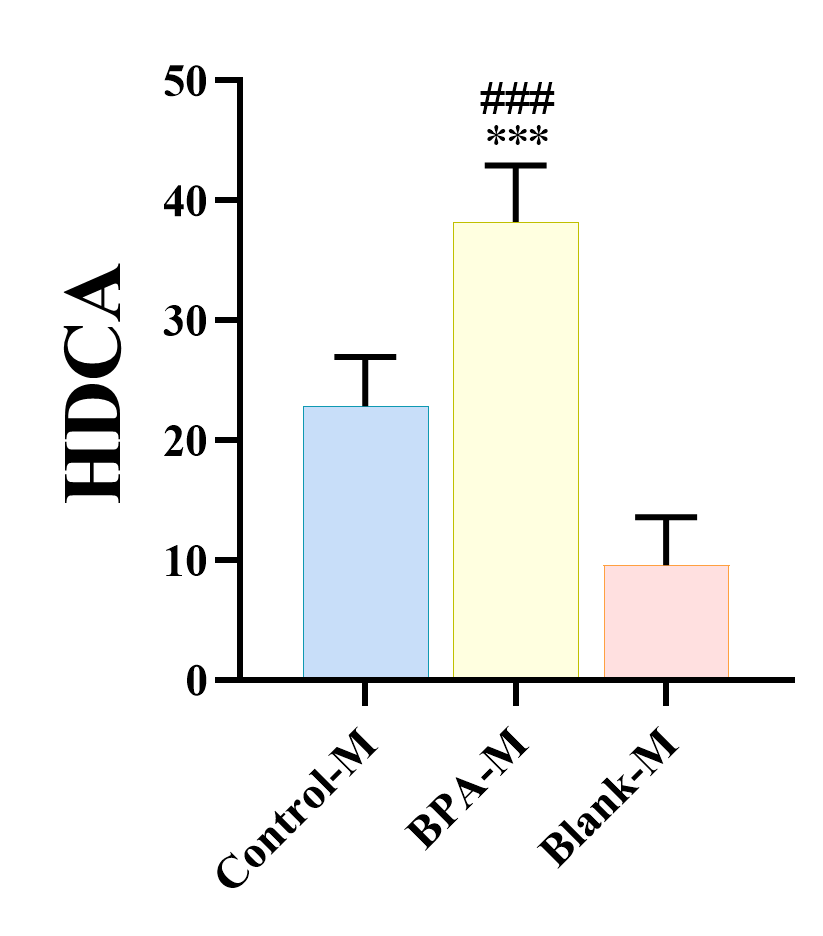

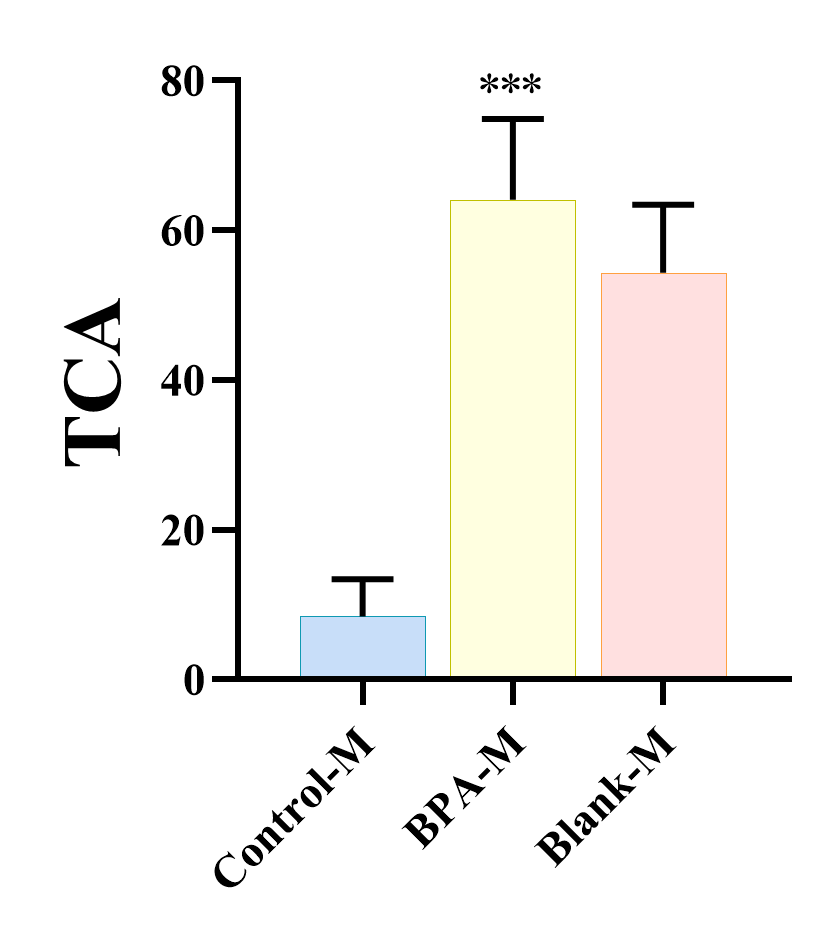

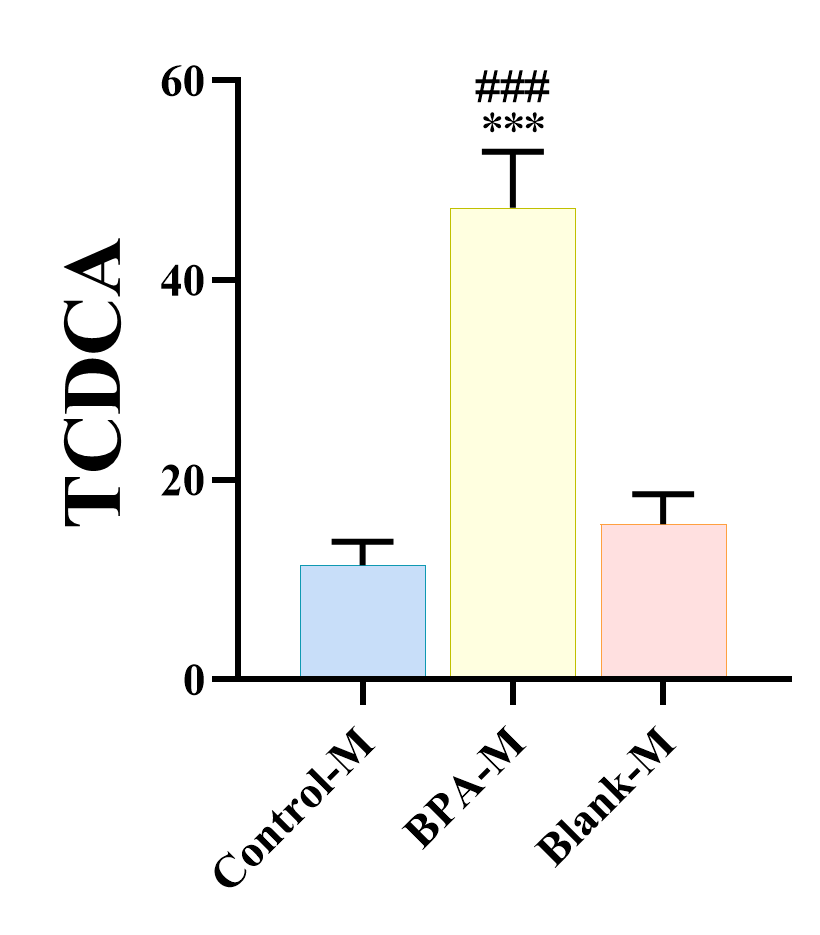

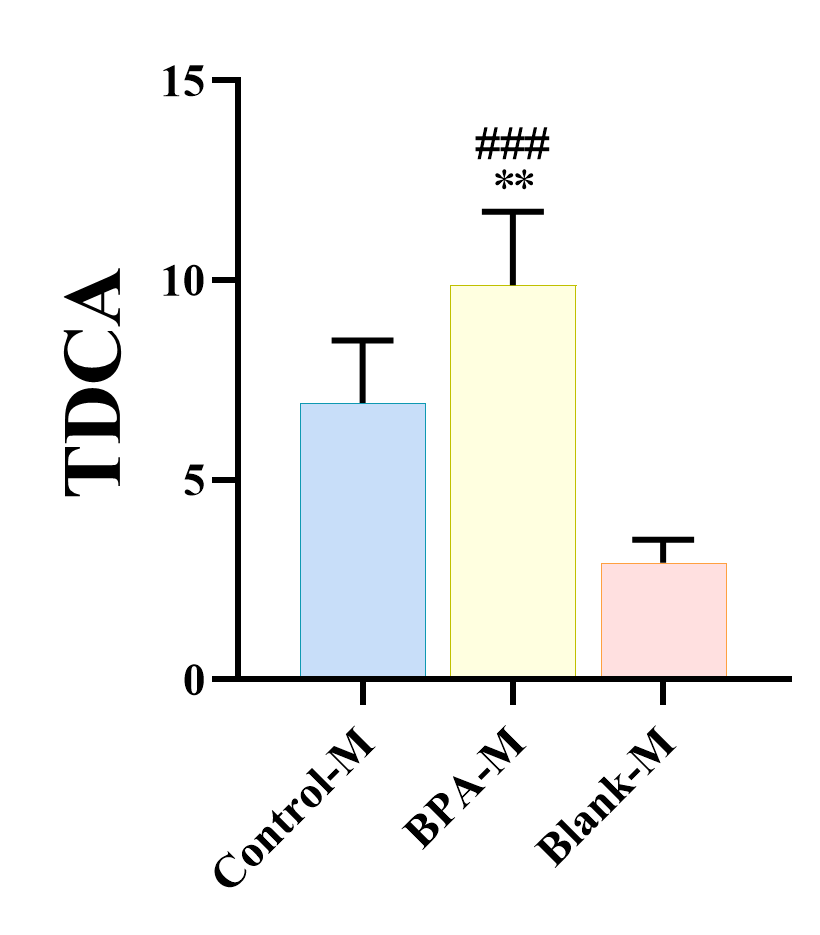

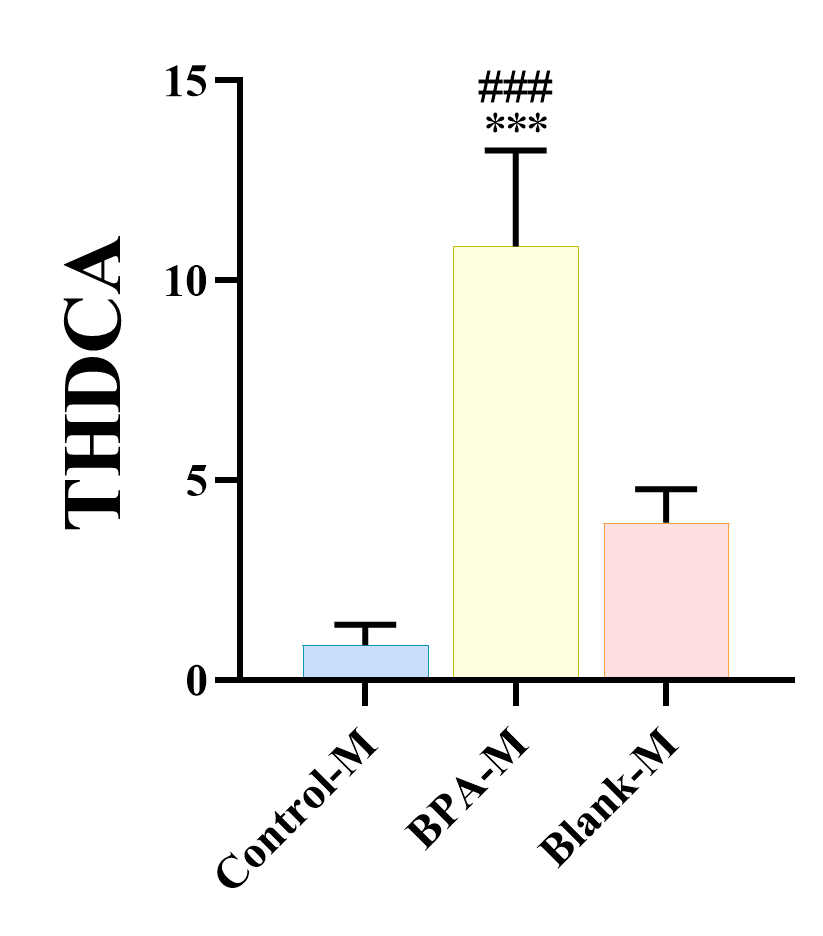

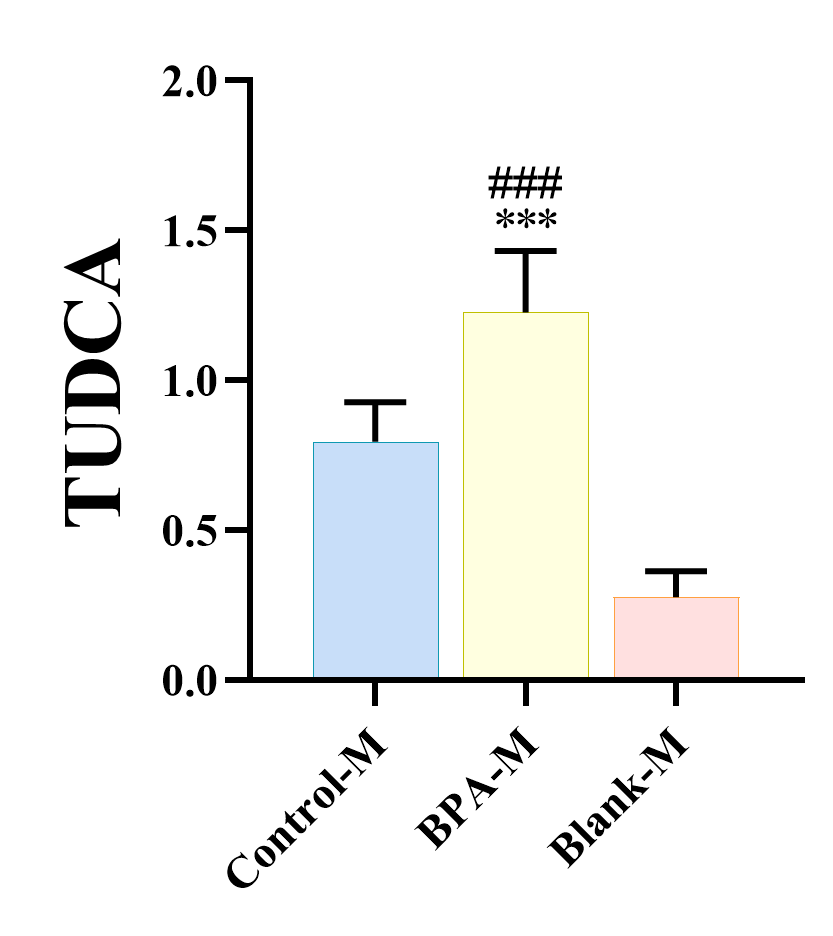

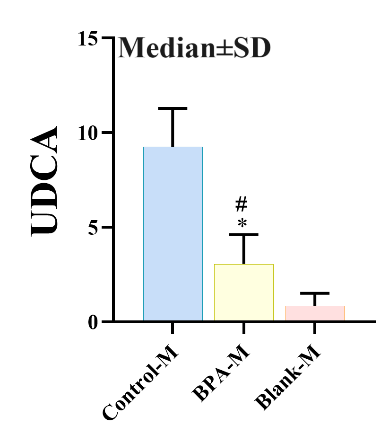

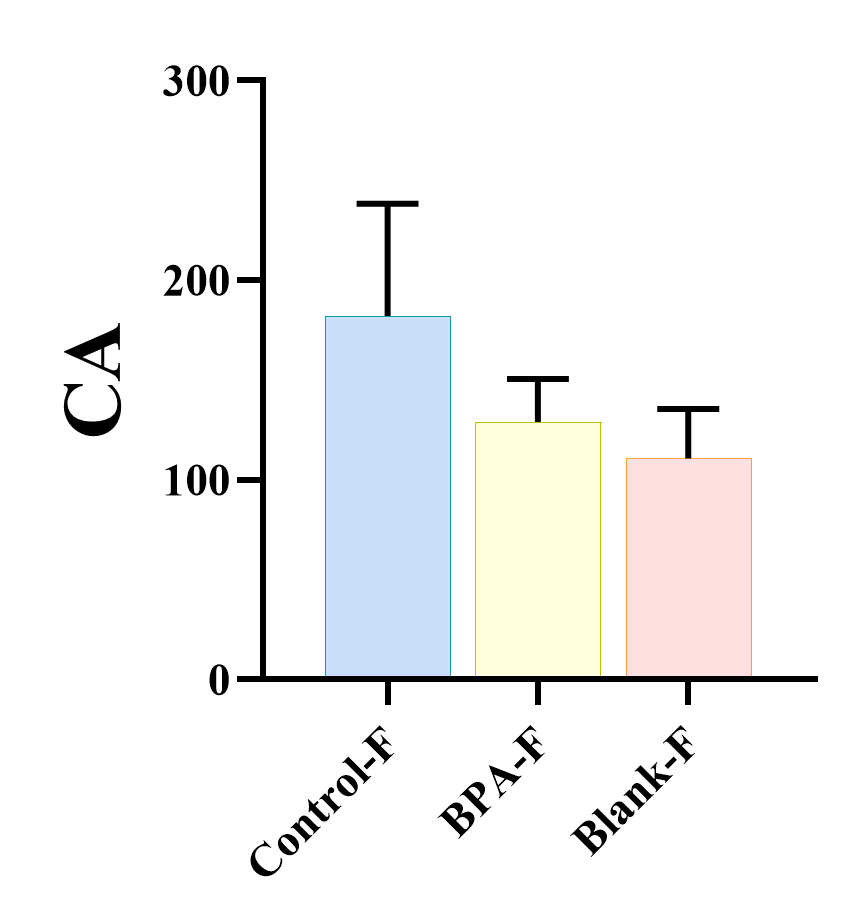

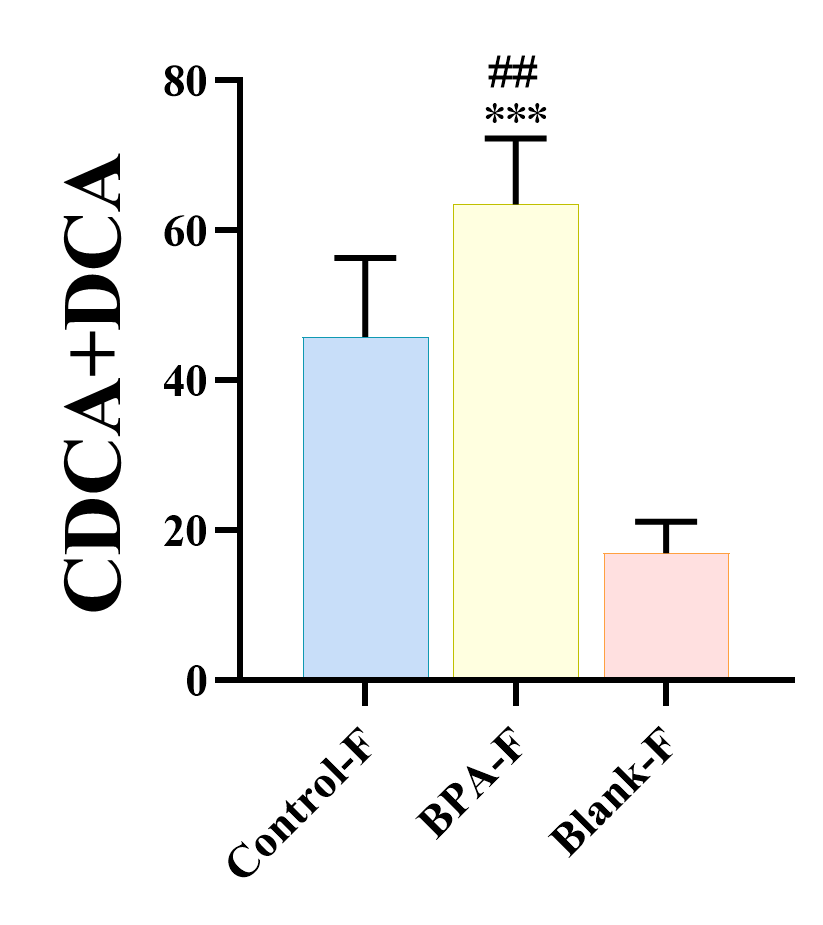

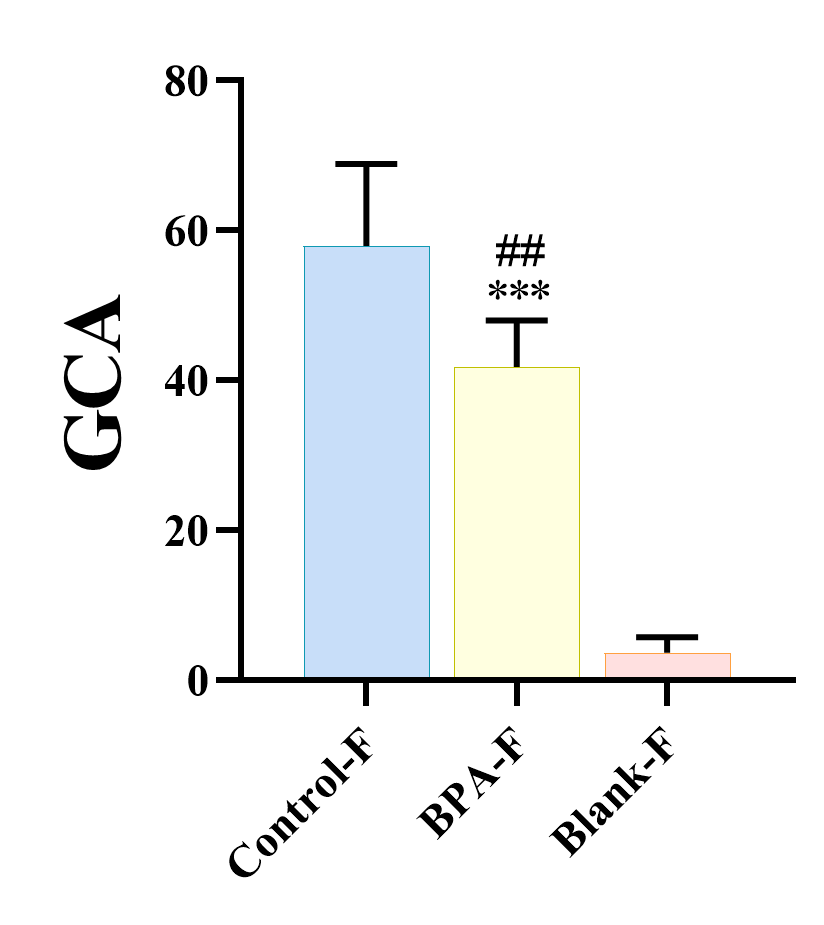

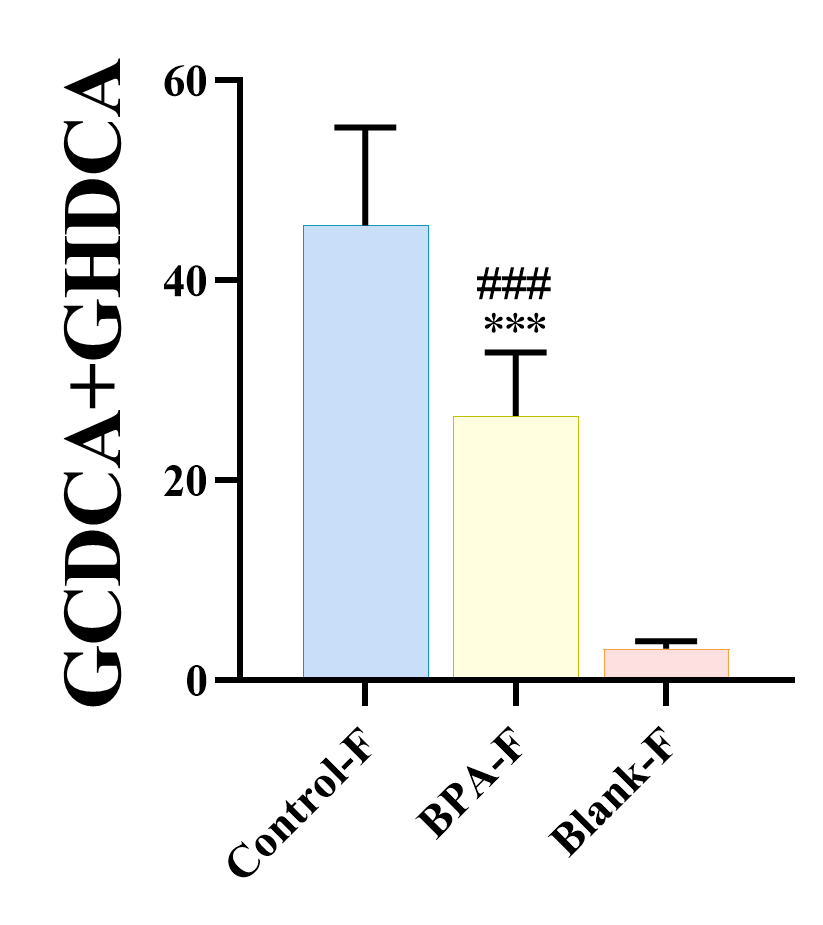

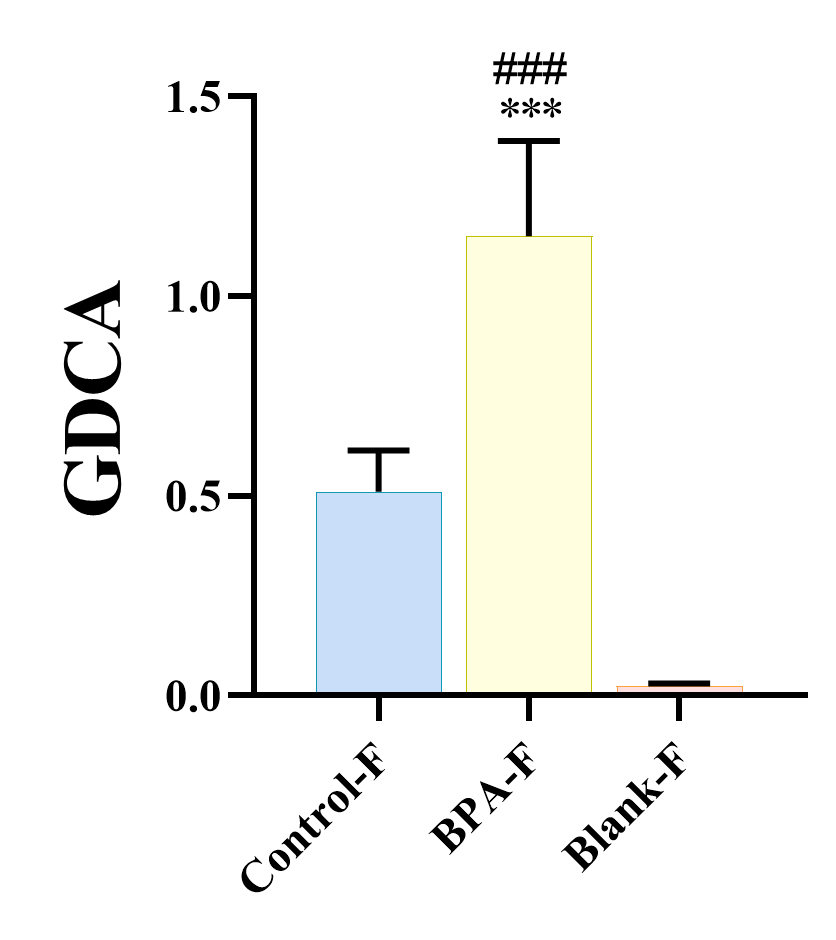

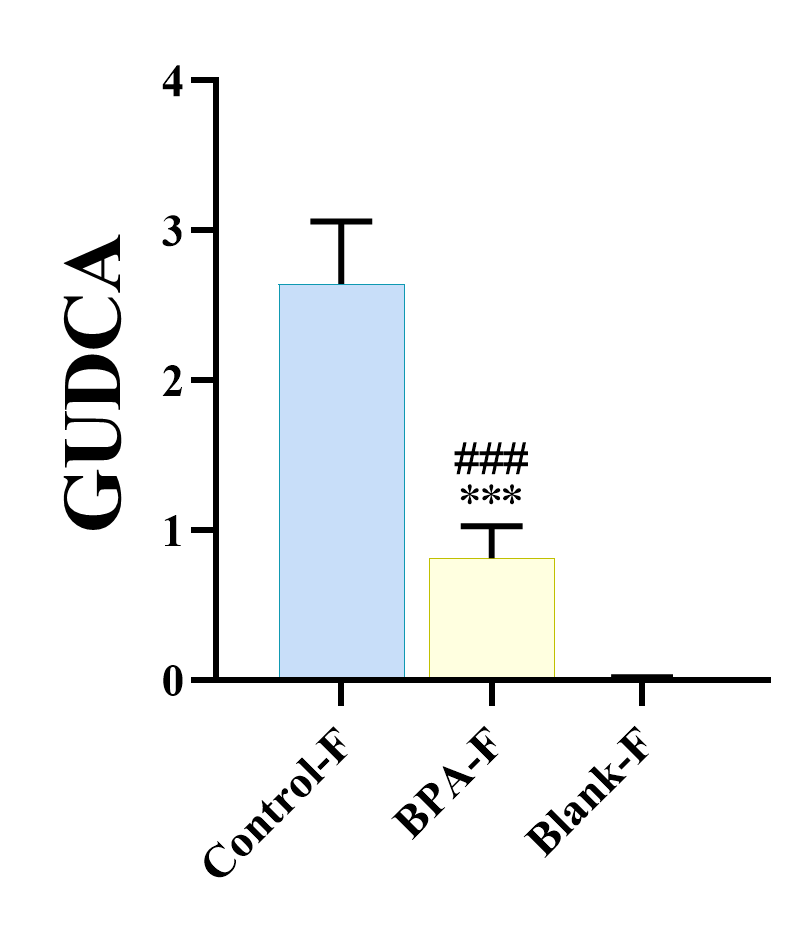

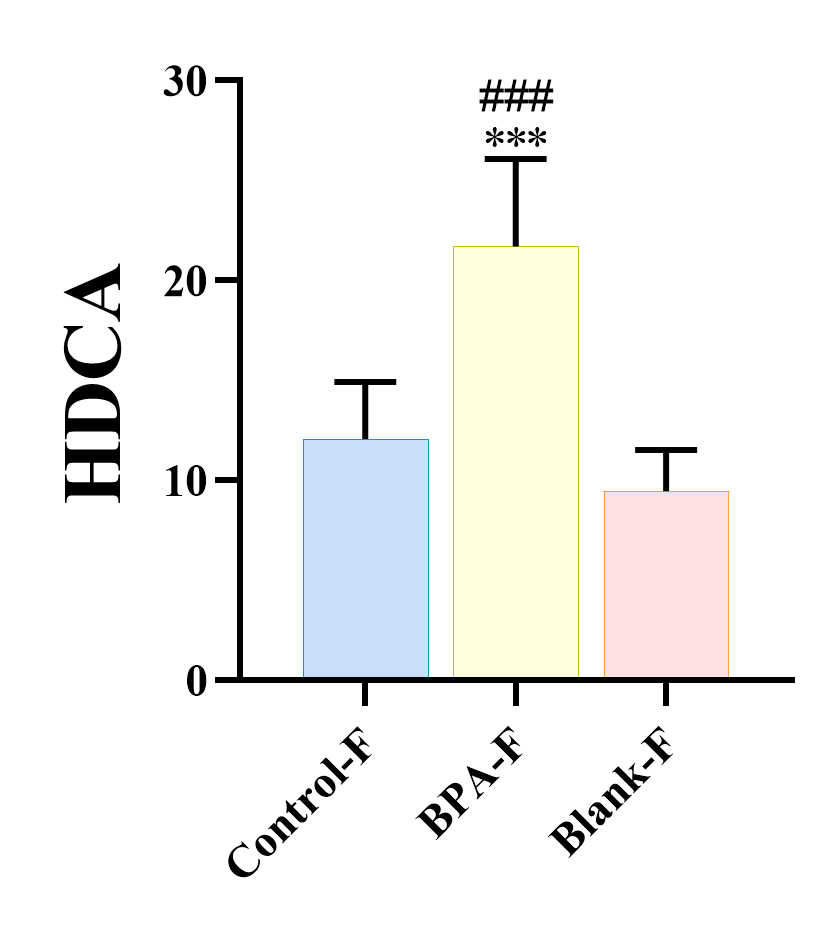

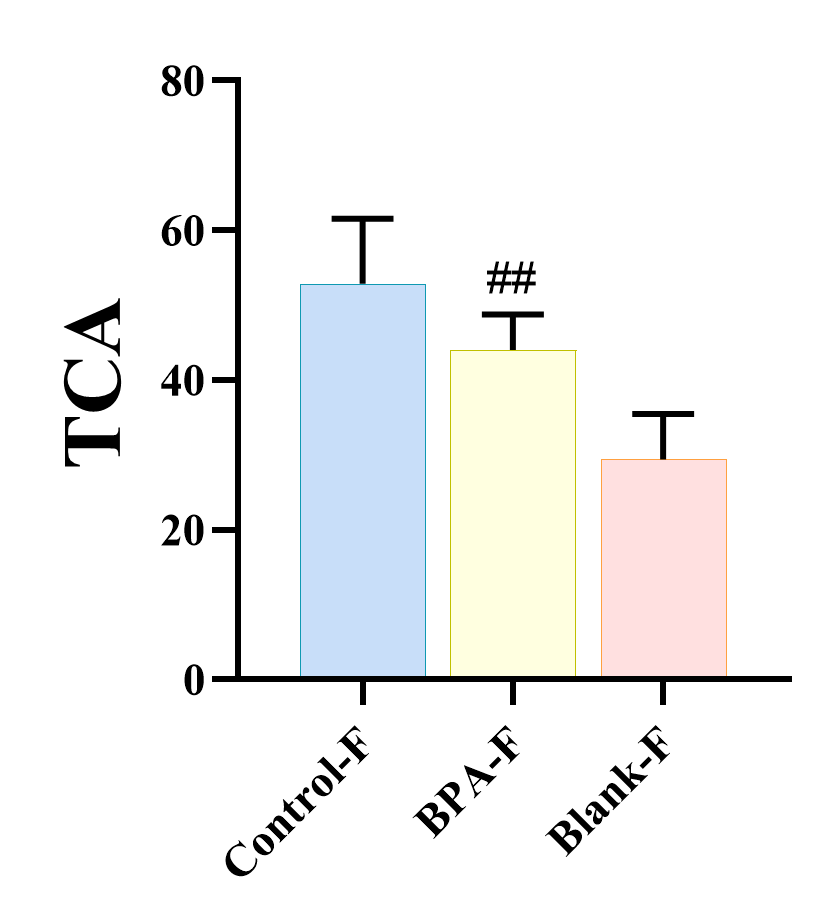

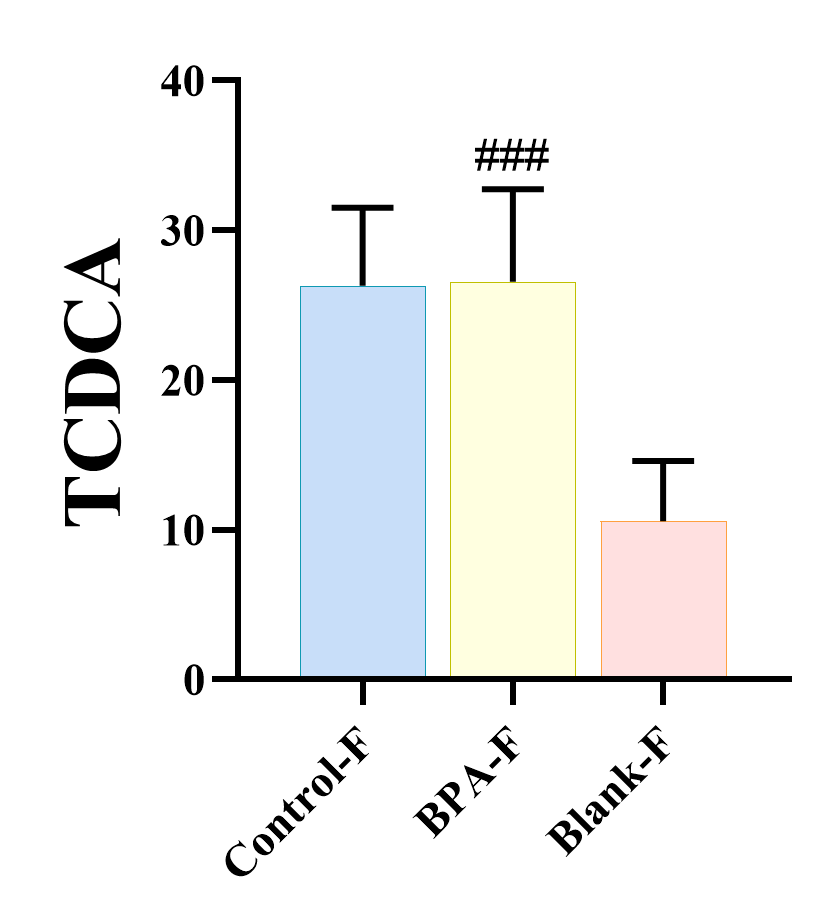

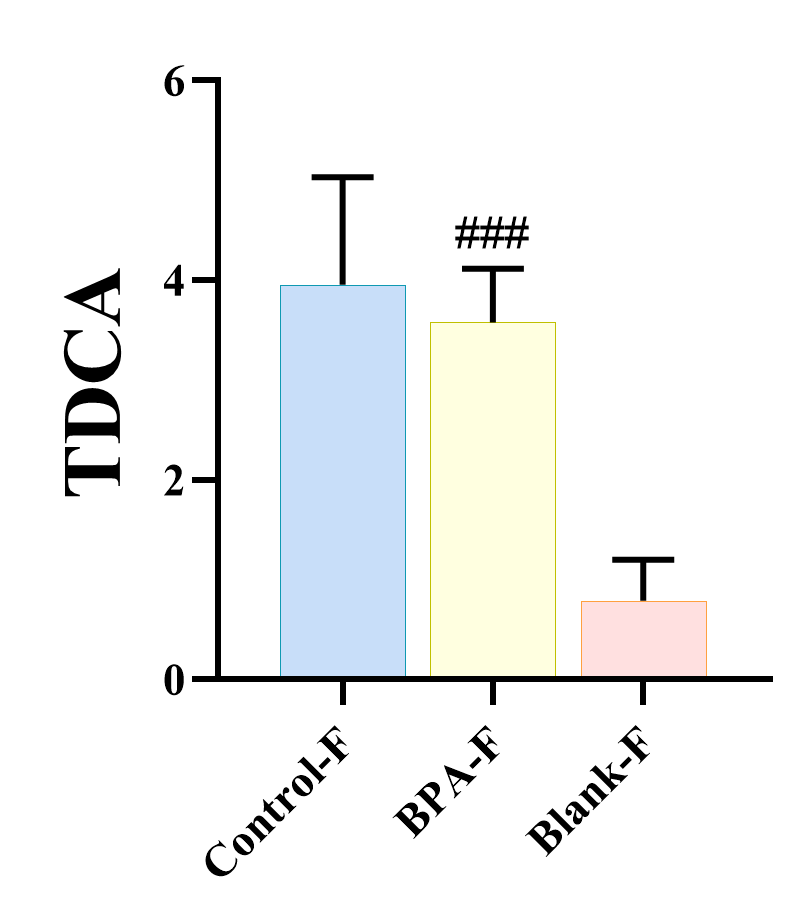

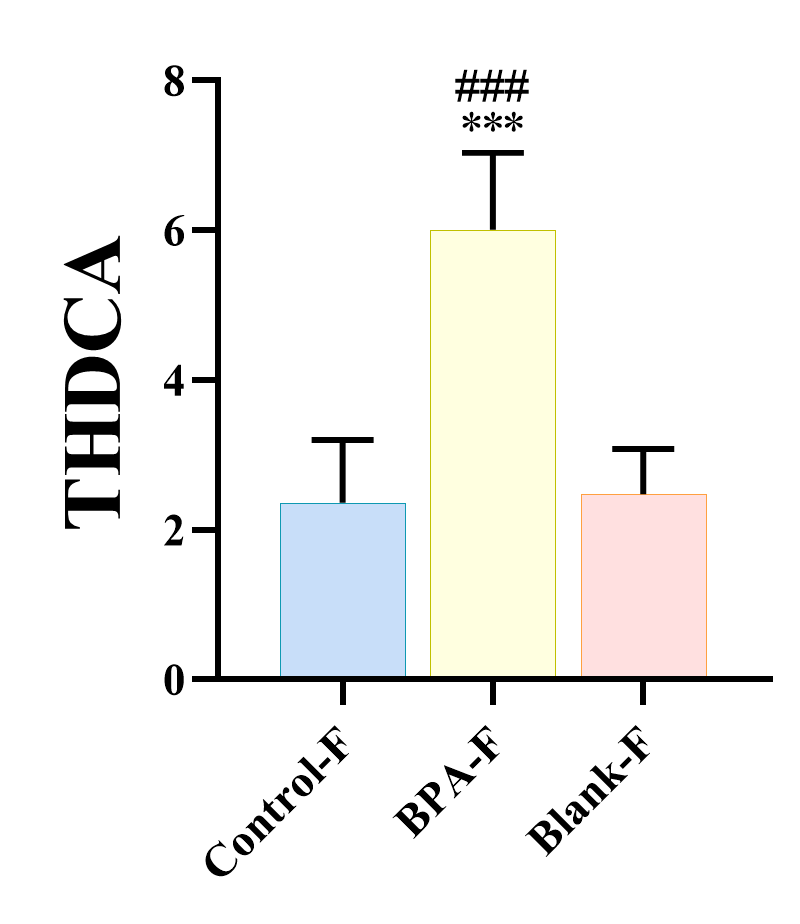

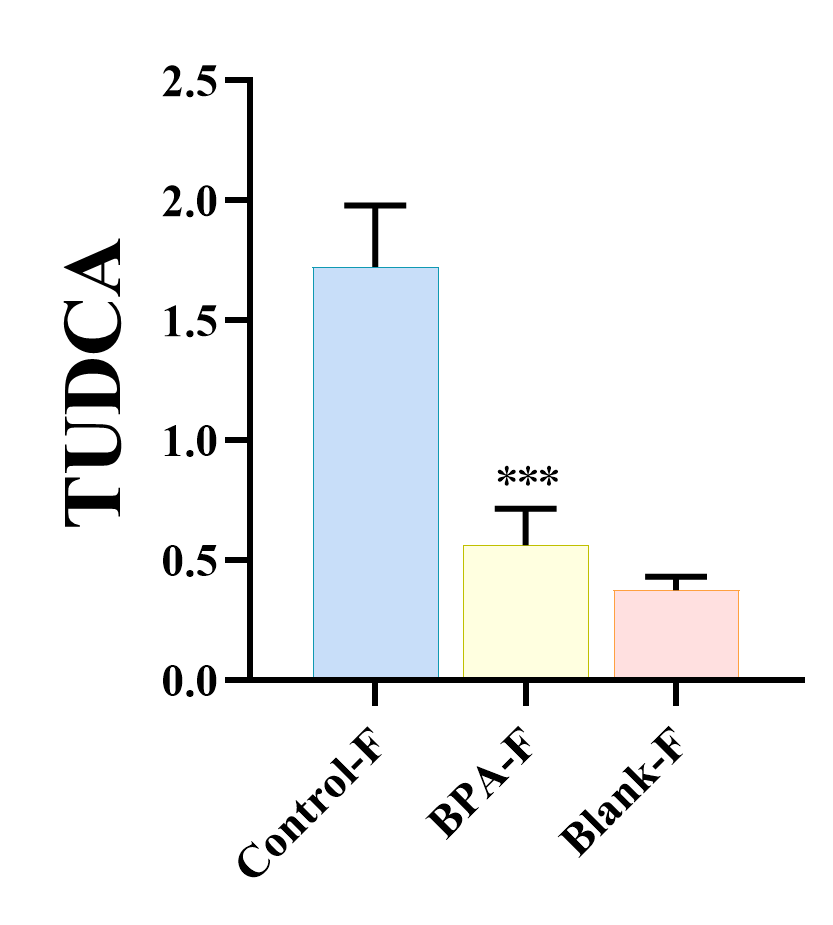

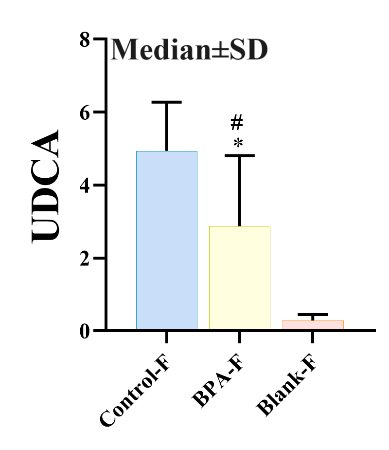


## **Fig. S7.** Relative level of BAs in the serum. Male group: Control-M, BPA-M, Blank-M; Female group: Control-F, BPA-F, Blank-F, n=6. The data did not follow a normal distribution (male group: GCA, UDCA; female group: UDCA) was presented as median and range, and analyzed through the Mann-Whitney U test. Others were presented as mean ± SD, and analyzed through ANOVA followed by Dunnett’s multiple comparison test. * p < 0.05, ** p < 0.01 and *** p < 0.001 versus the Control group; ^#^ p < 0.05, ^##^ p < 0.01 and ^###^ p < 0.001 versus the Blank group.

**
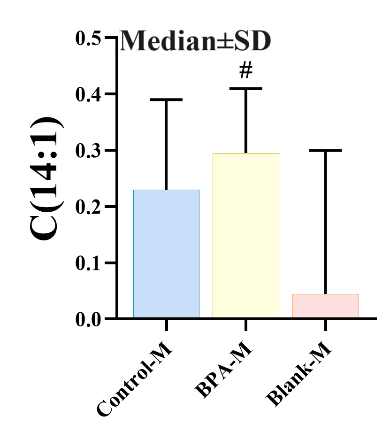

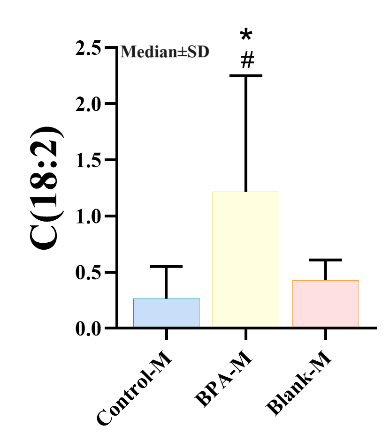

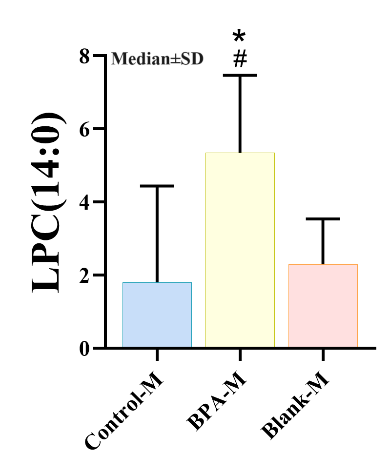
**

**
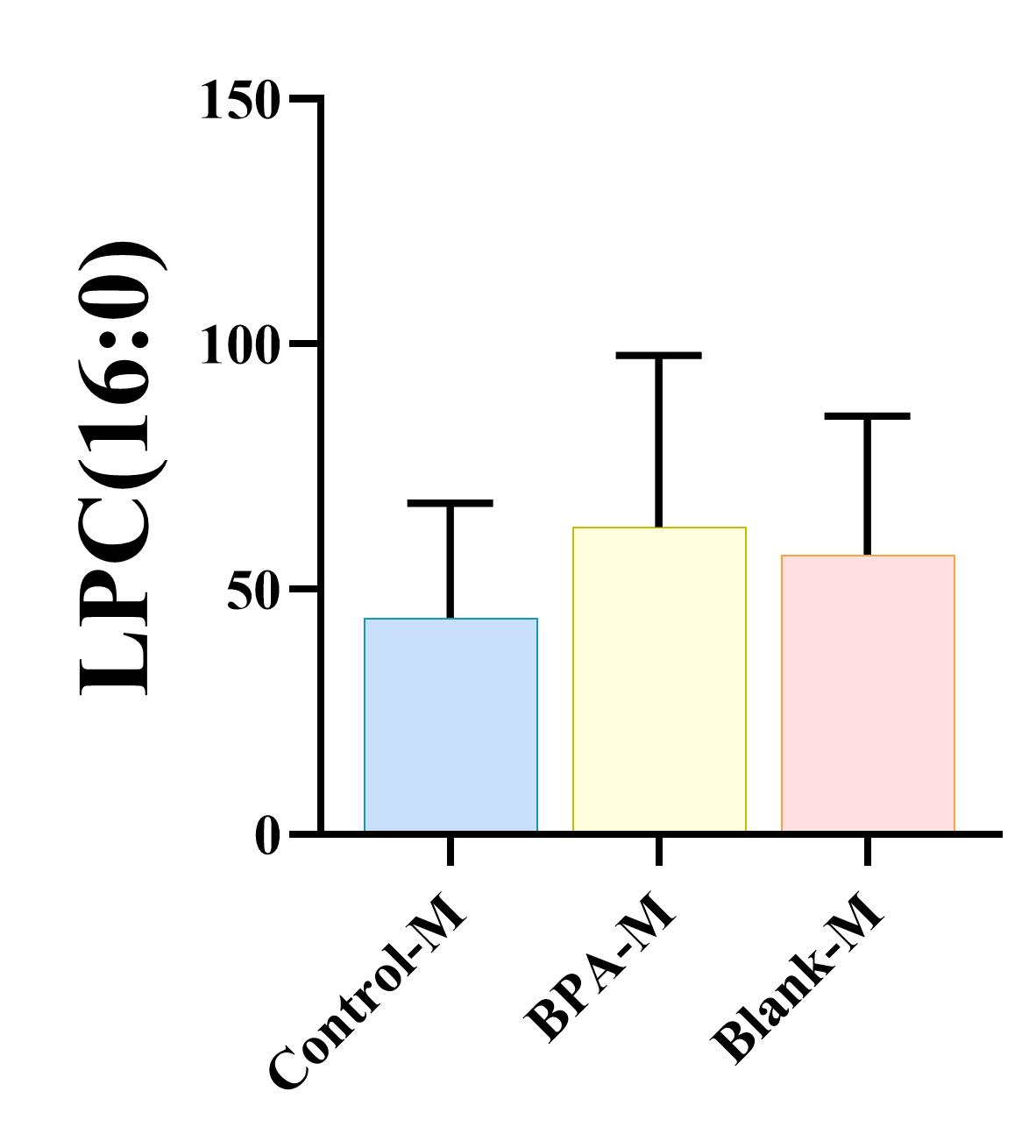

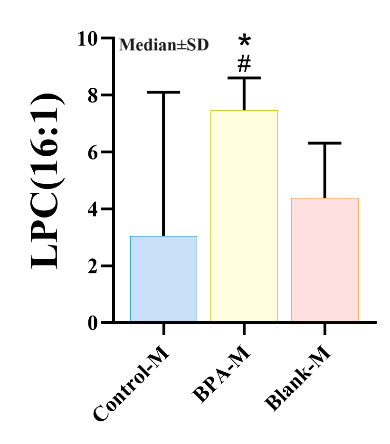

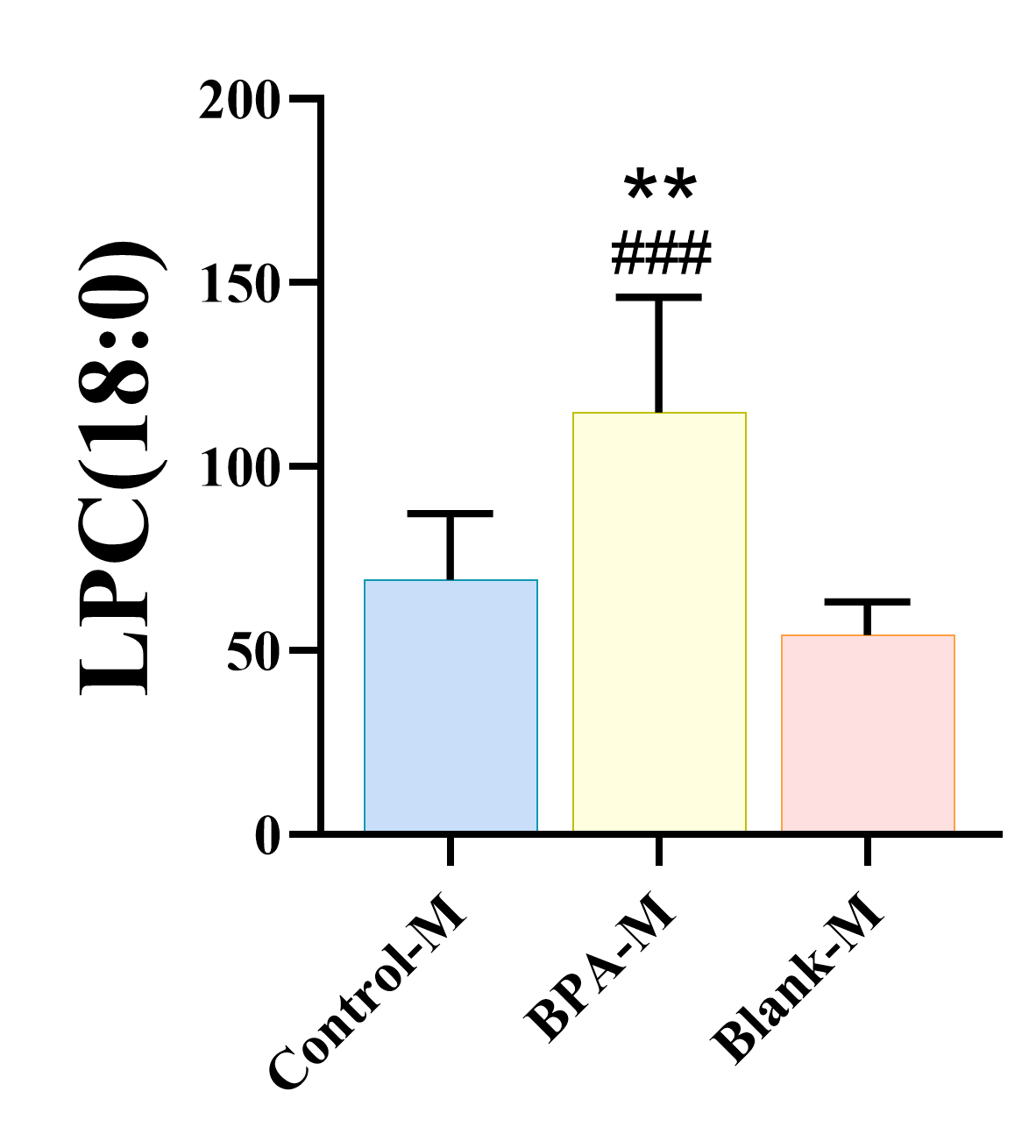
**

**
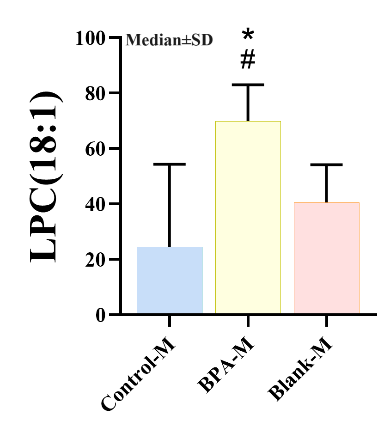

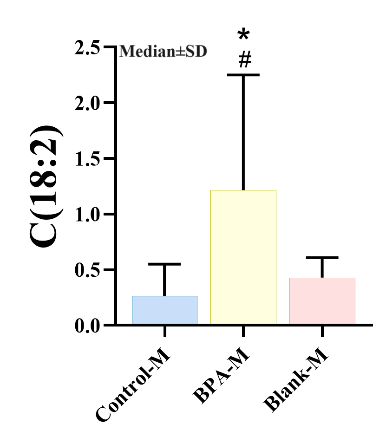

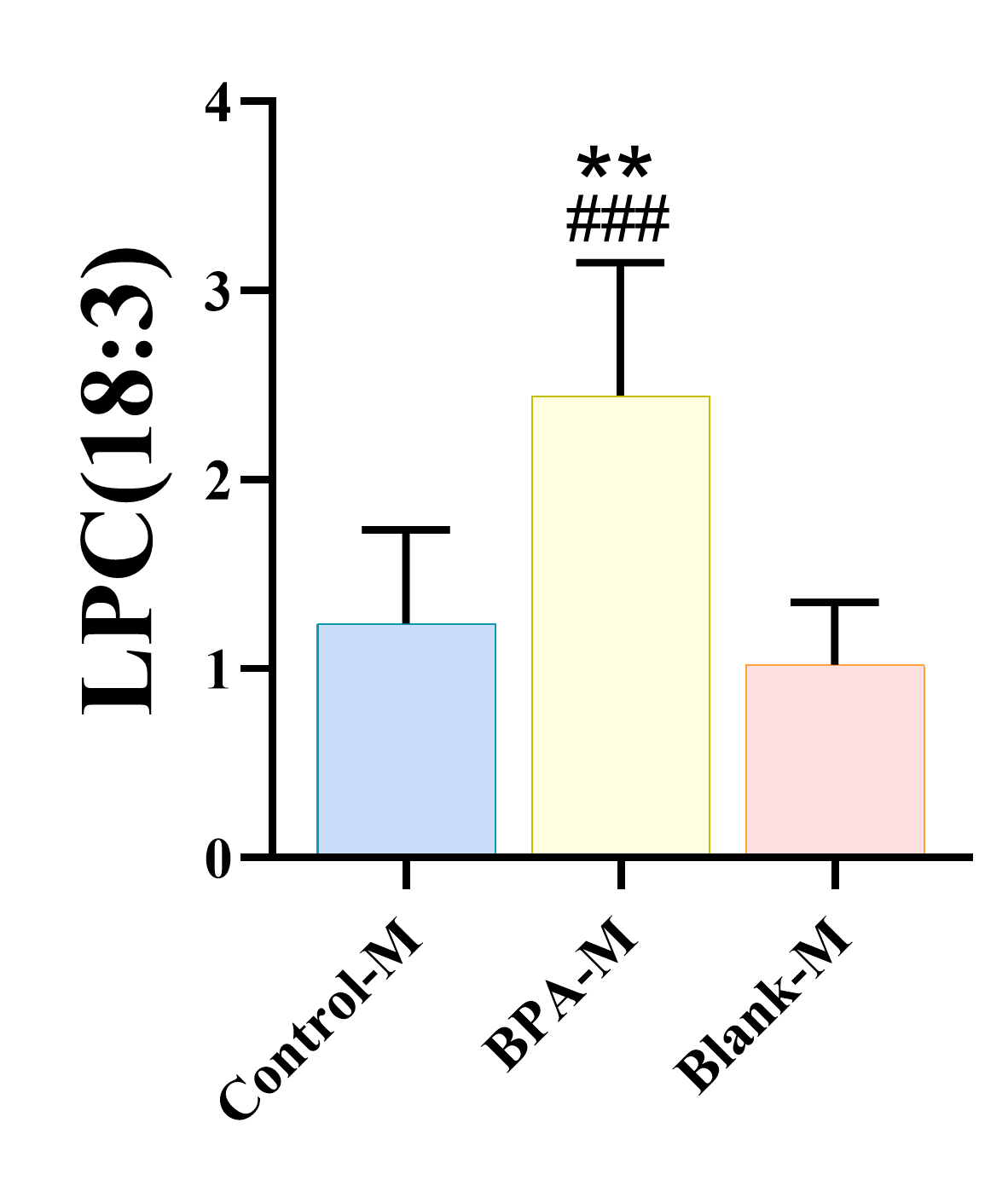
**

**
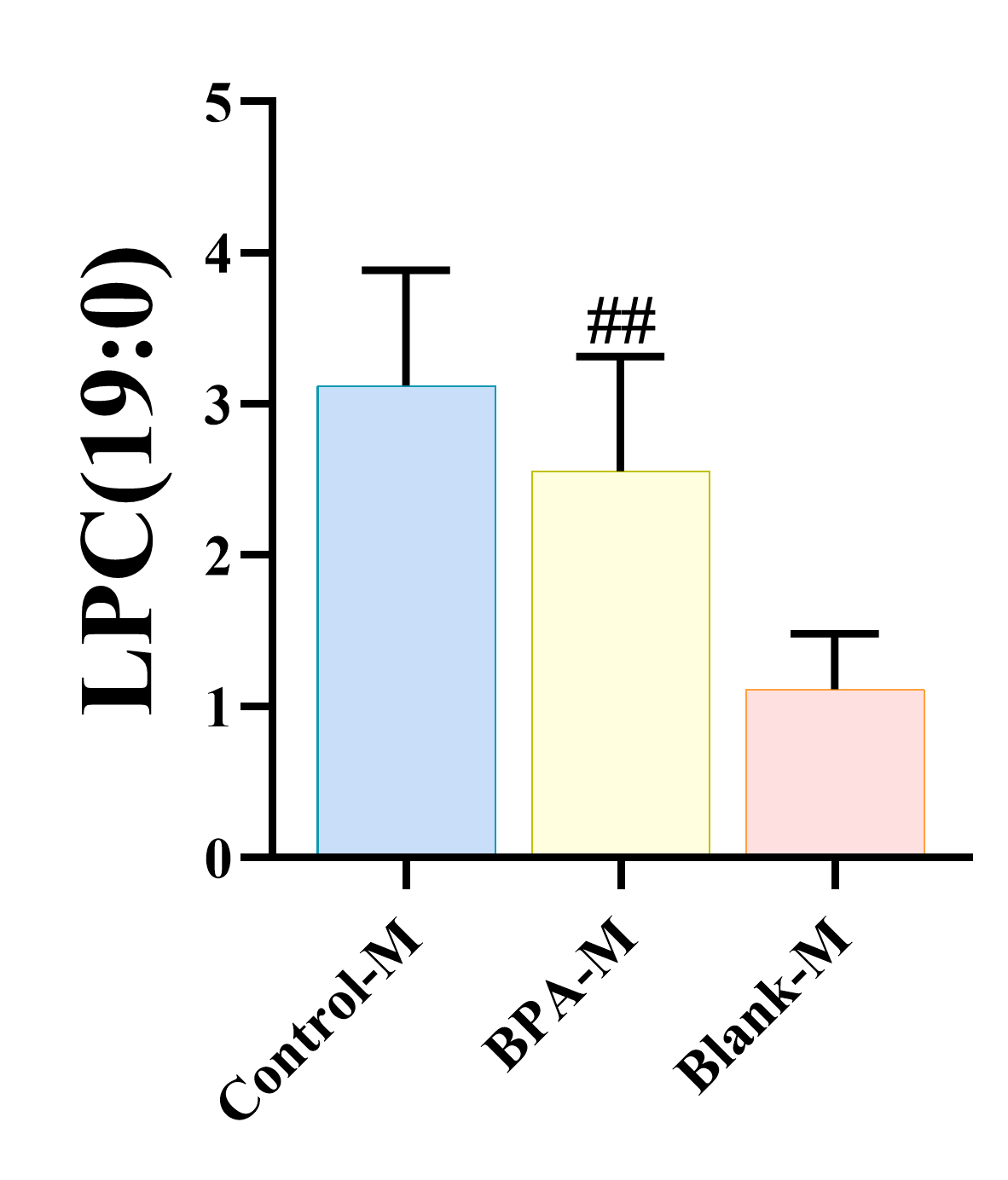

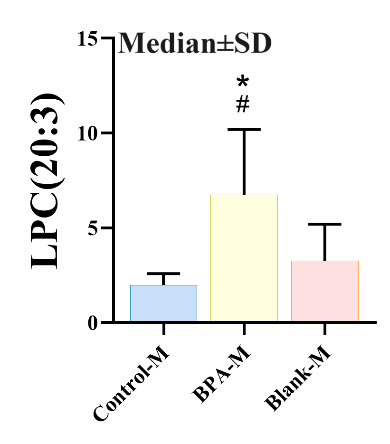

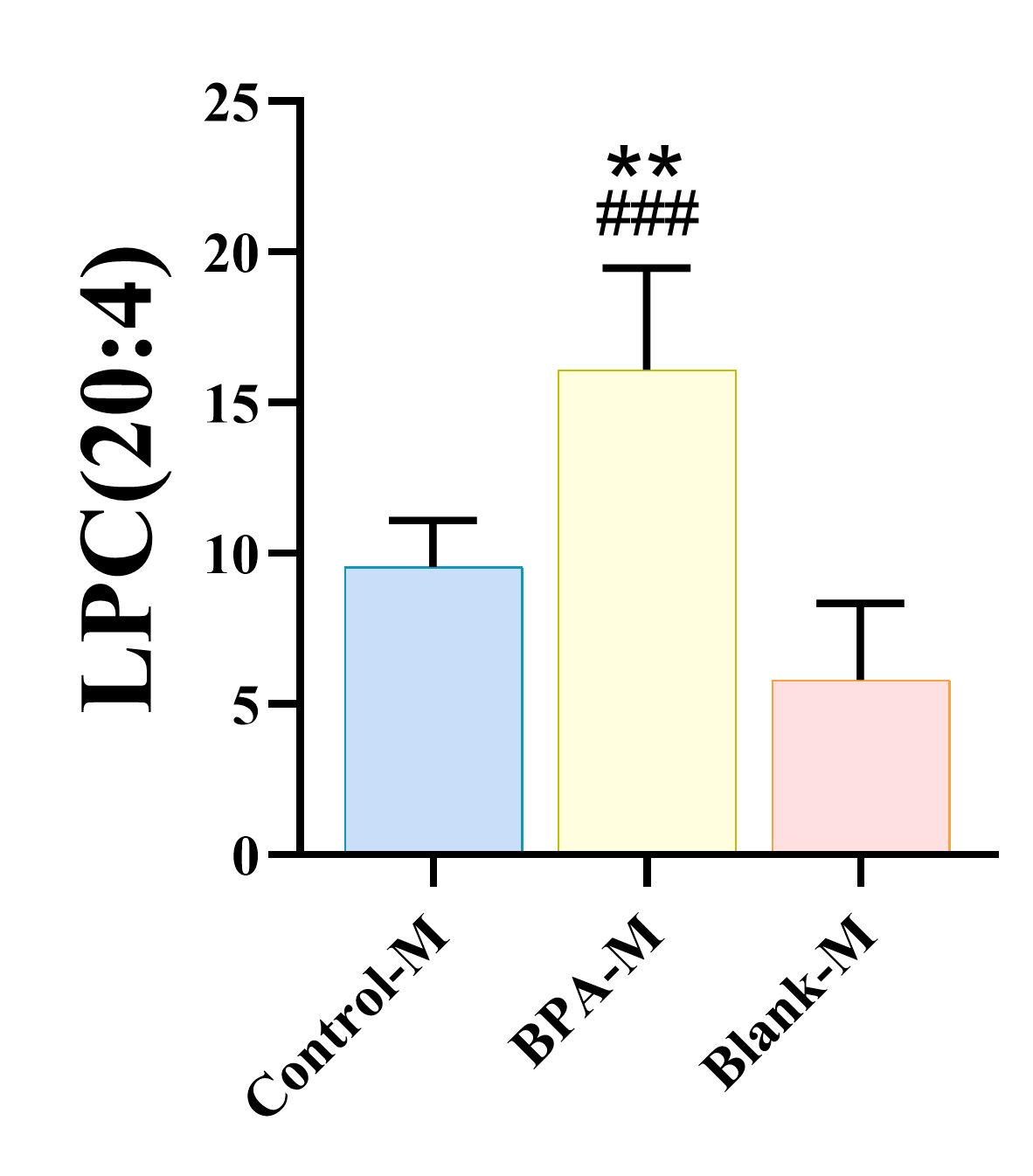
**

**
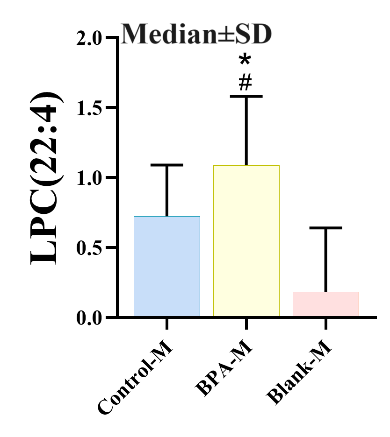
**

**
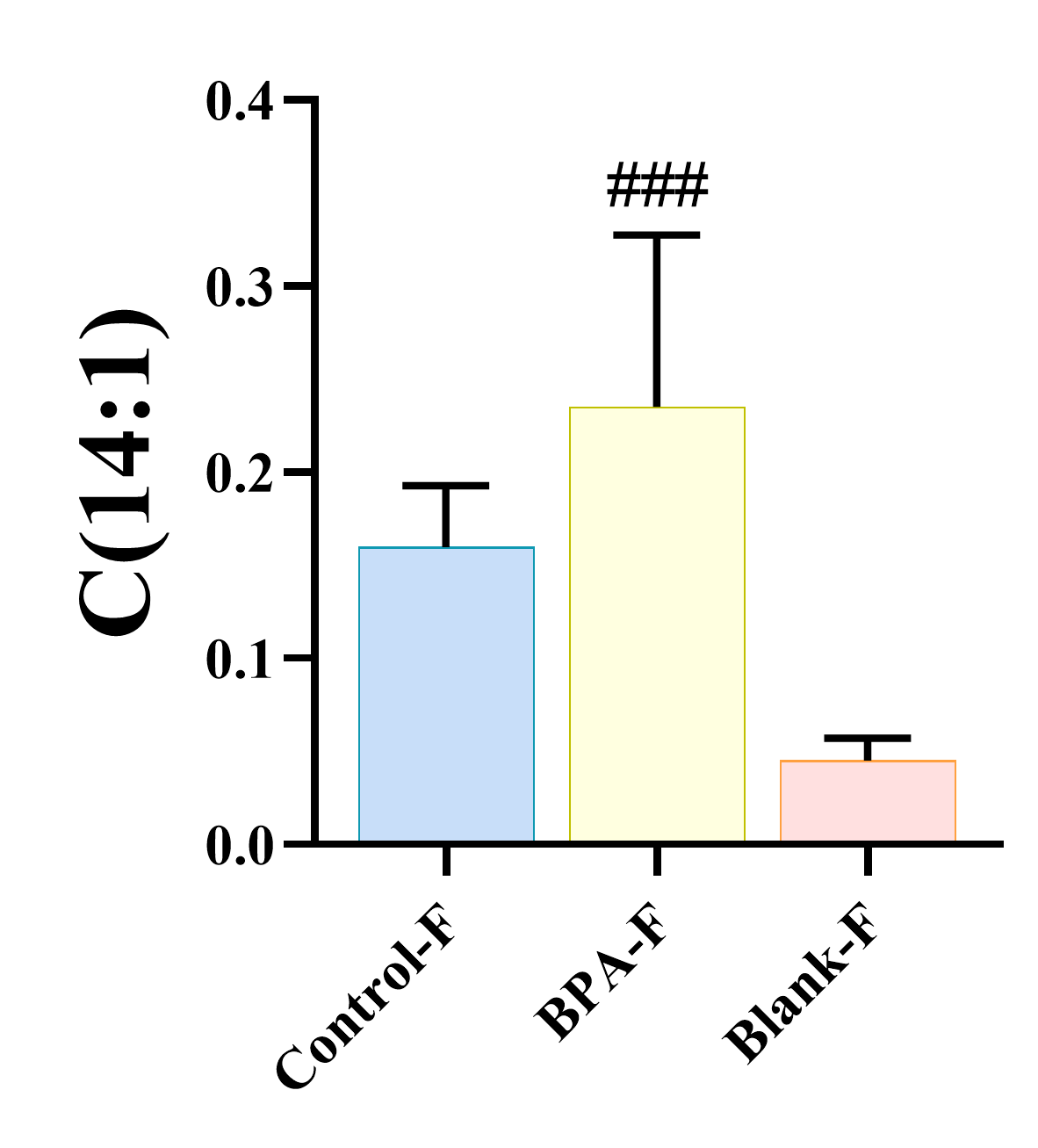

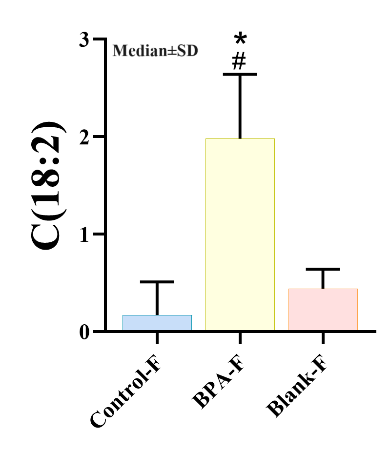

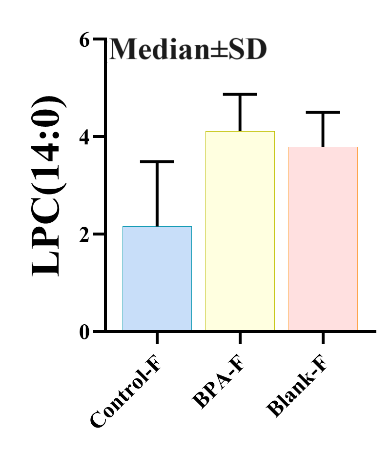
**

**
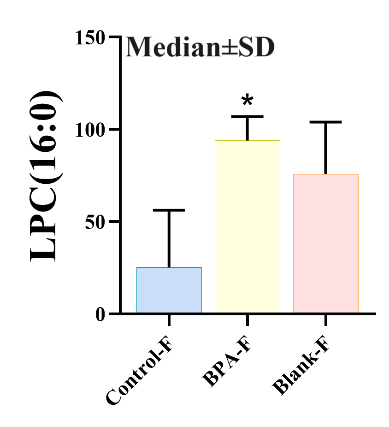

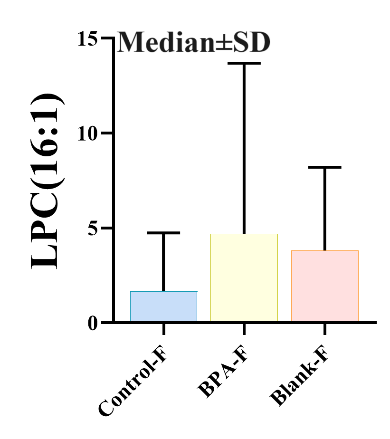

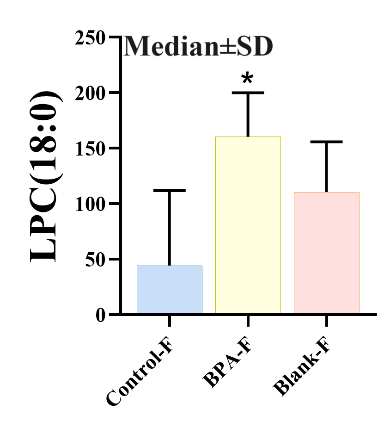

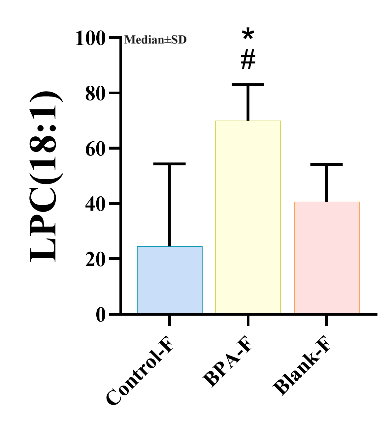

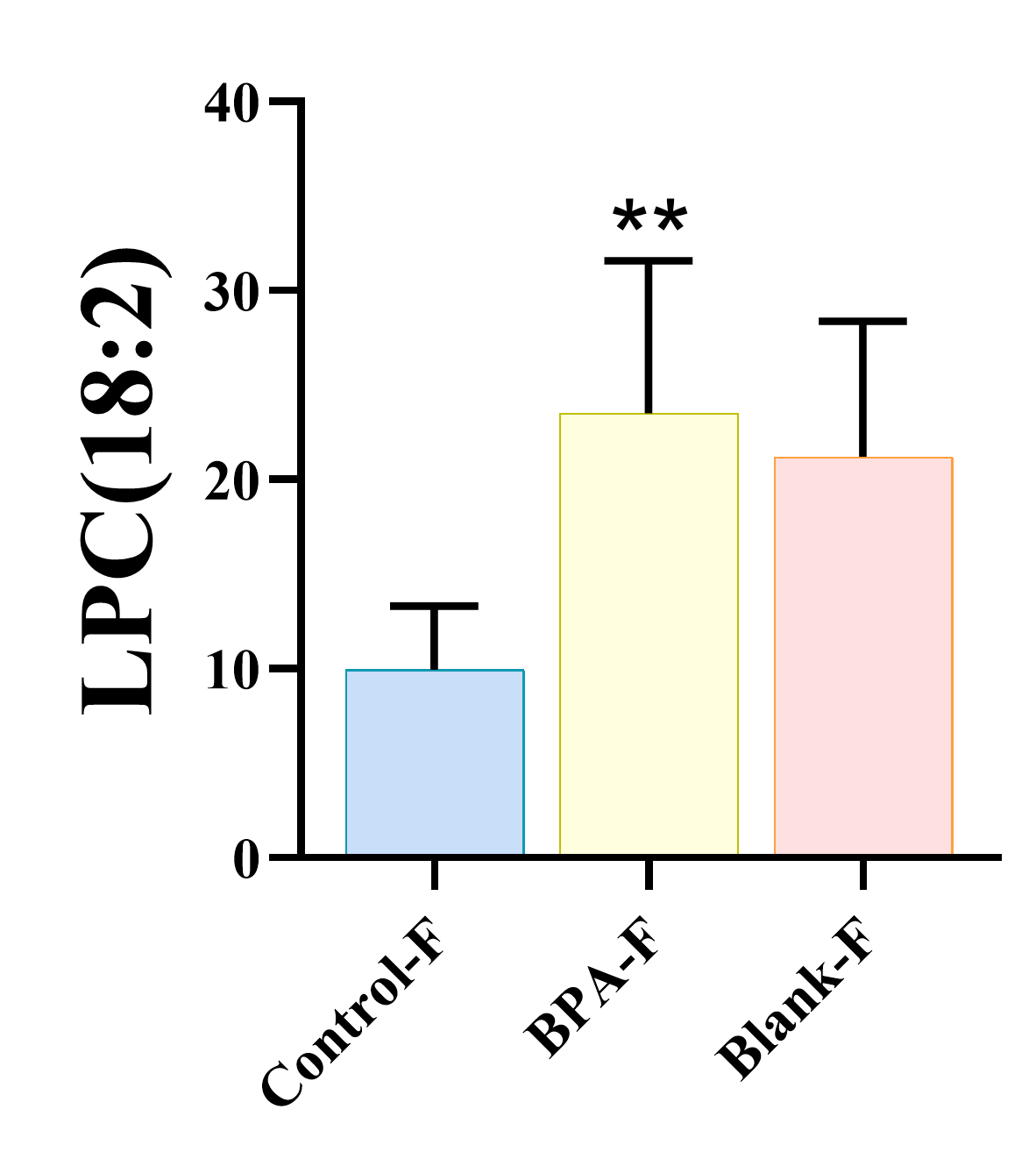

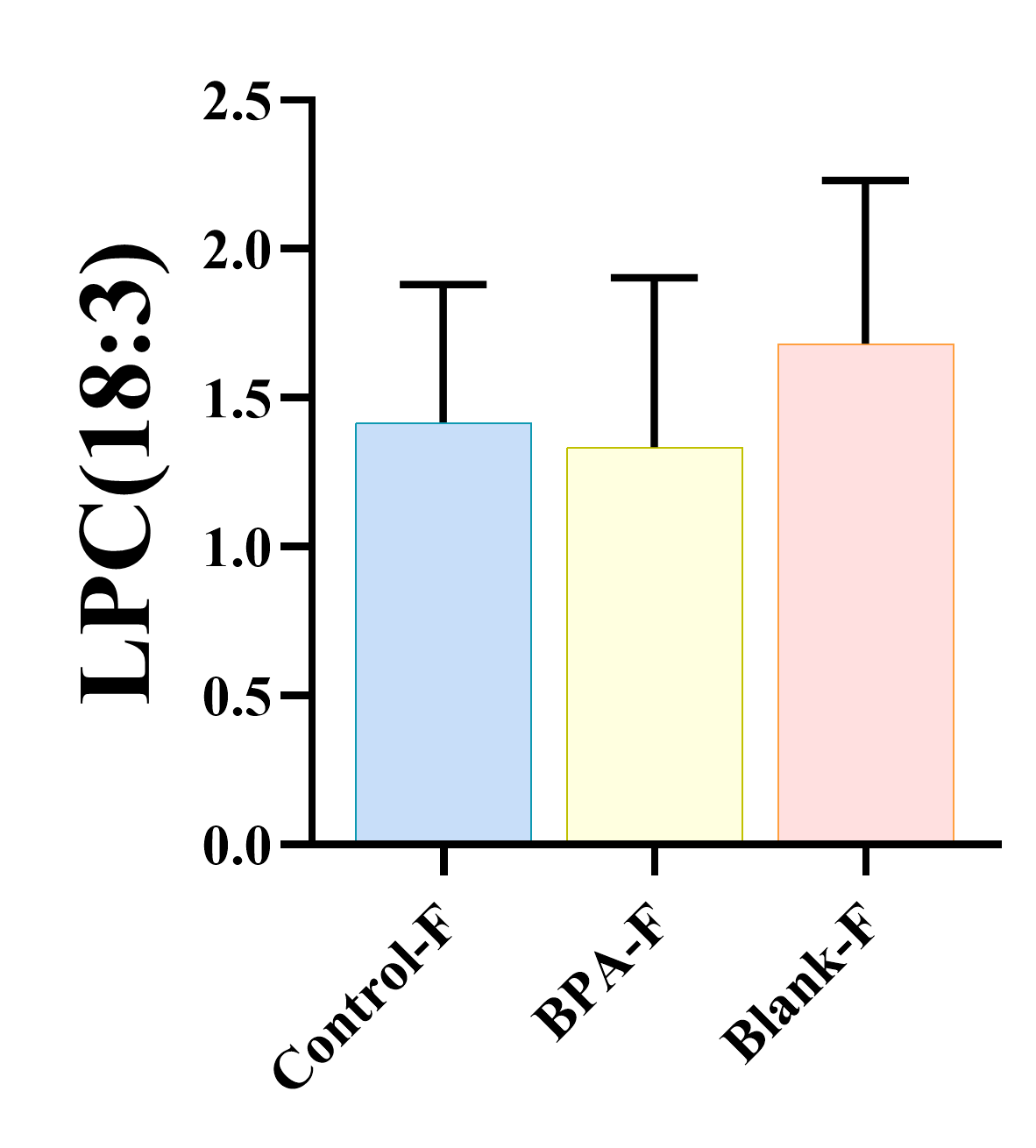
**

**
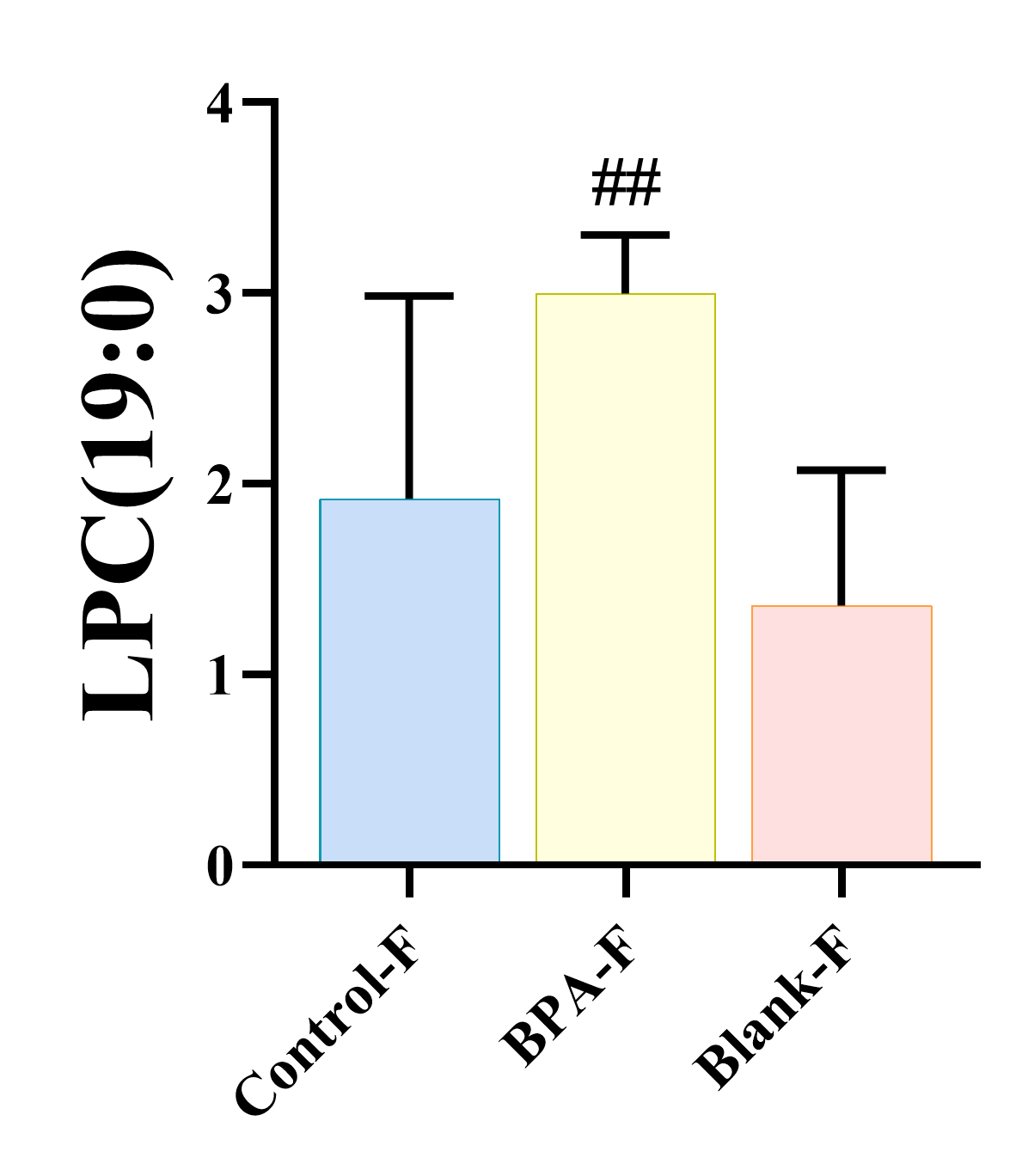

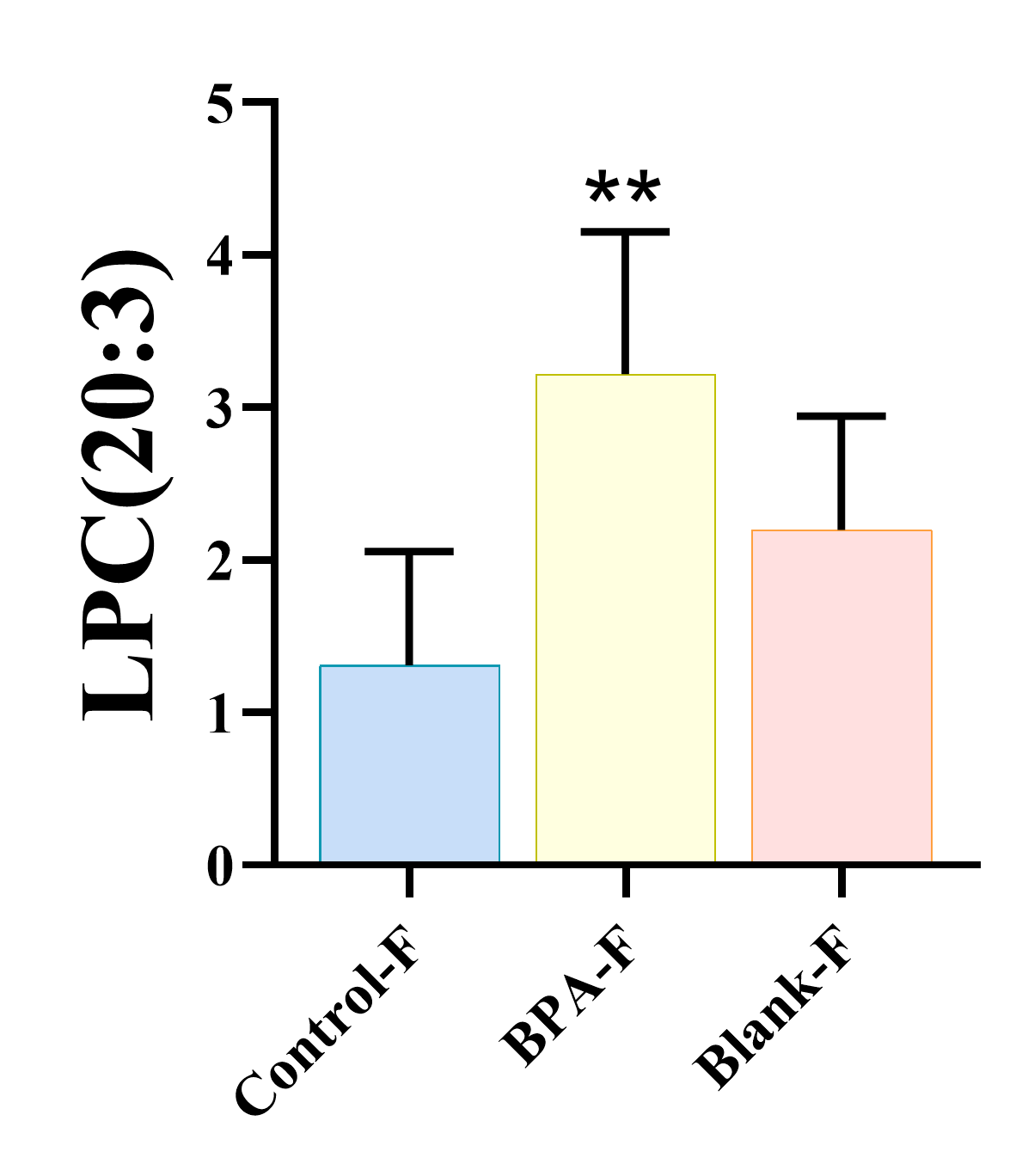

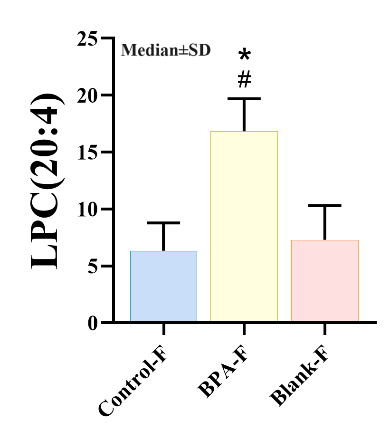
**

**
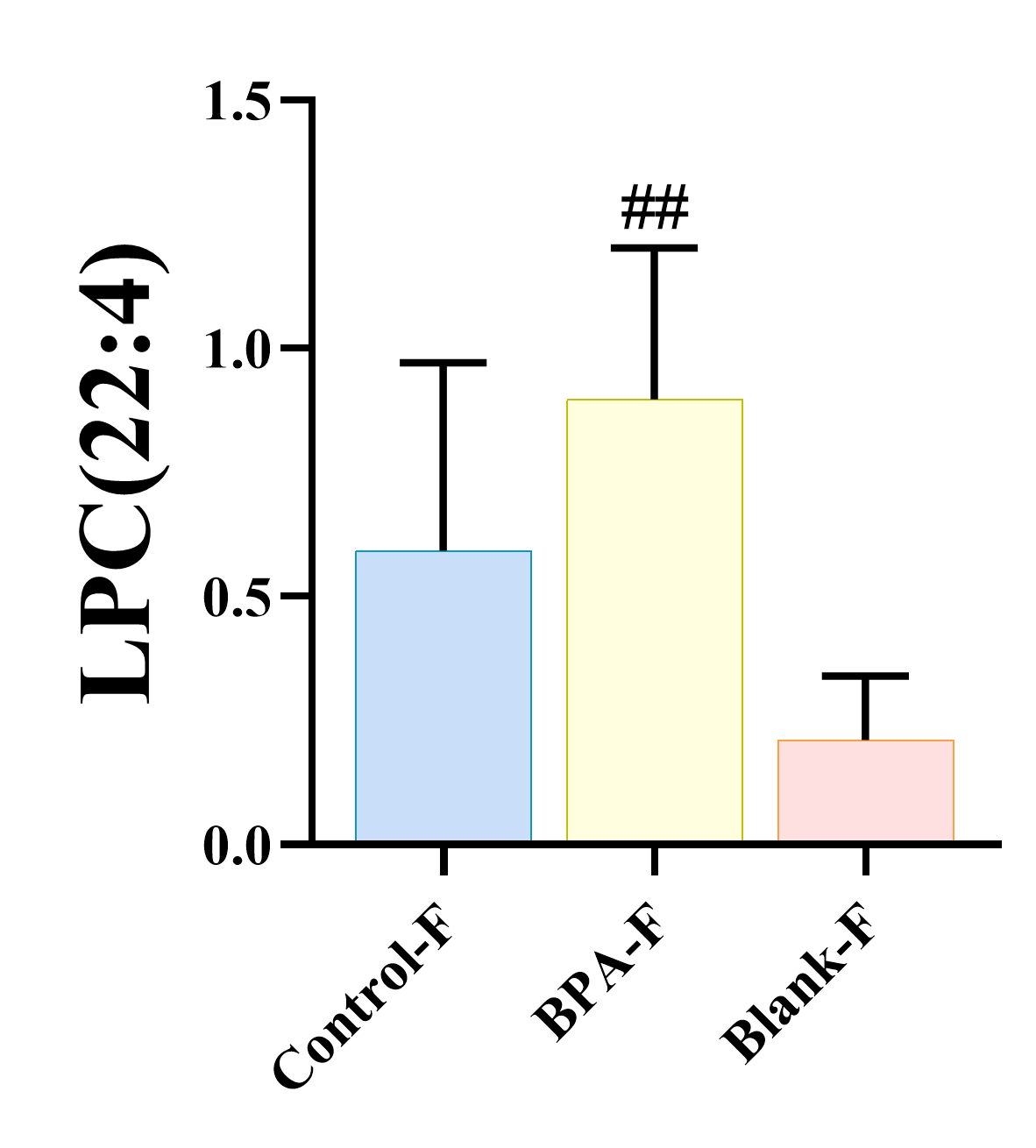
**

## **Fig. S8.** Relative level of LPCs in the serum. Male group: Control-M, BPA-M, Blank-M; Female group: Control-F, BPA-F, Blank-F, n=6. The data did not follow a normal distribution was presented as median and range, and analyzed through the Mann-Whitney U test. Others were presented as mean ± SD, and analyzed through ANOVA followed by Dunnett’s multiple comparison test. * p < 0.05, ** p < 0.01 and *** p < 0.001 versus the Control group; ^#^ p < 0.05, ^##^ p < 0.01 and ^###^ p < 0.001 versus the Blank group.


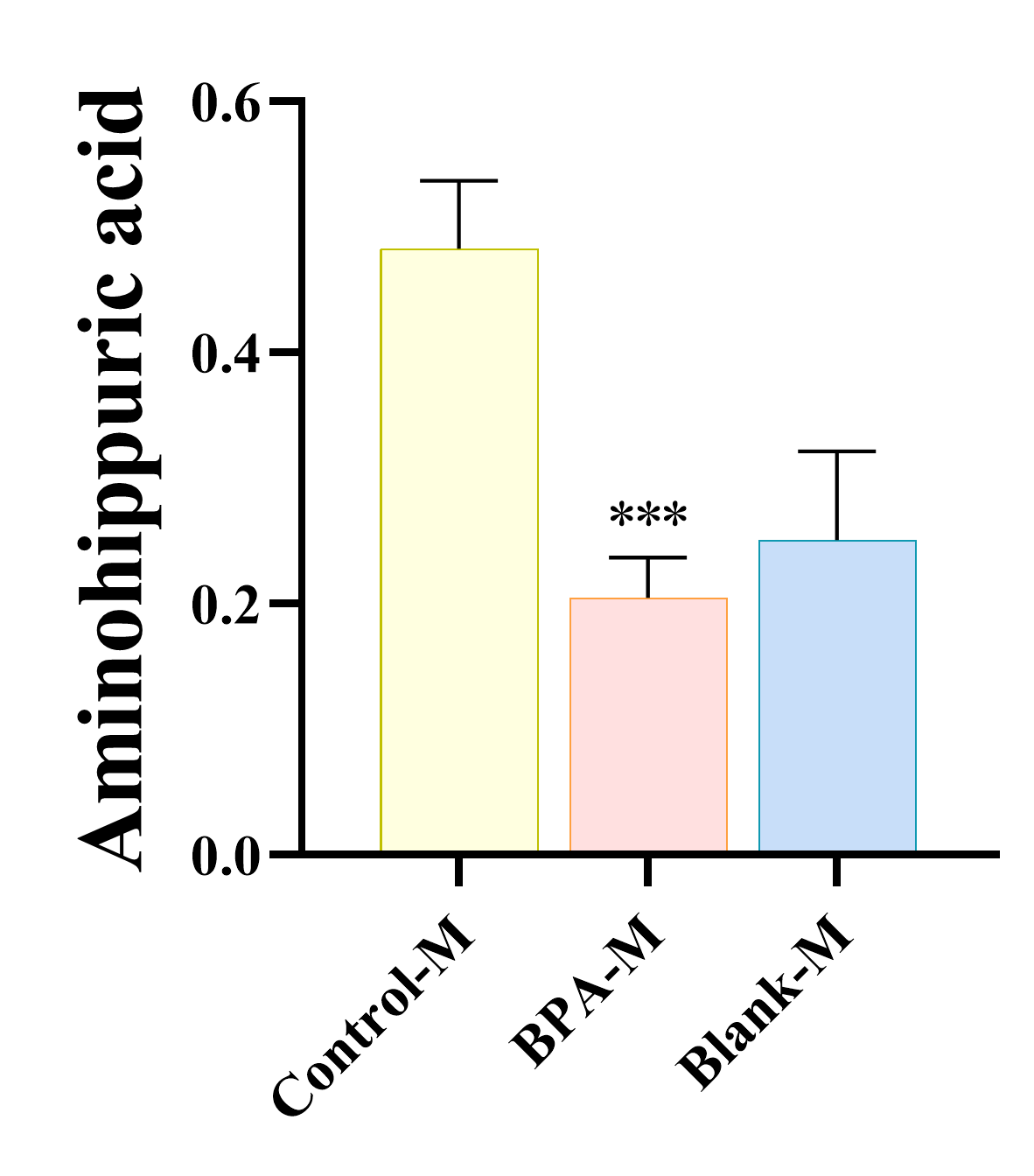

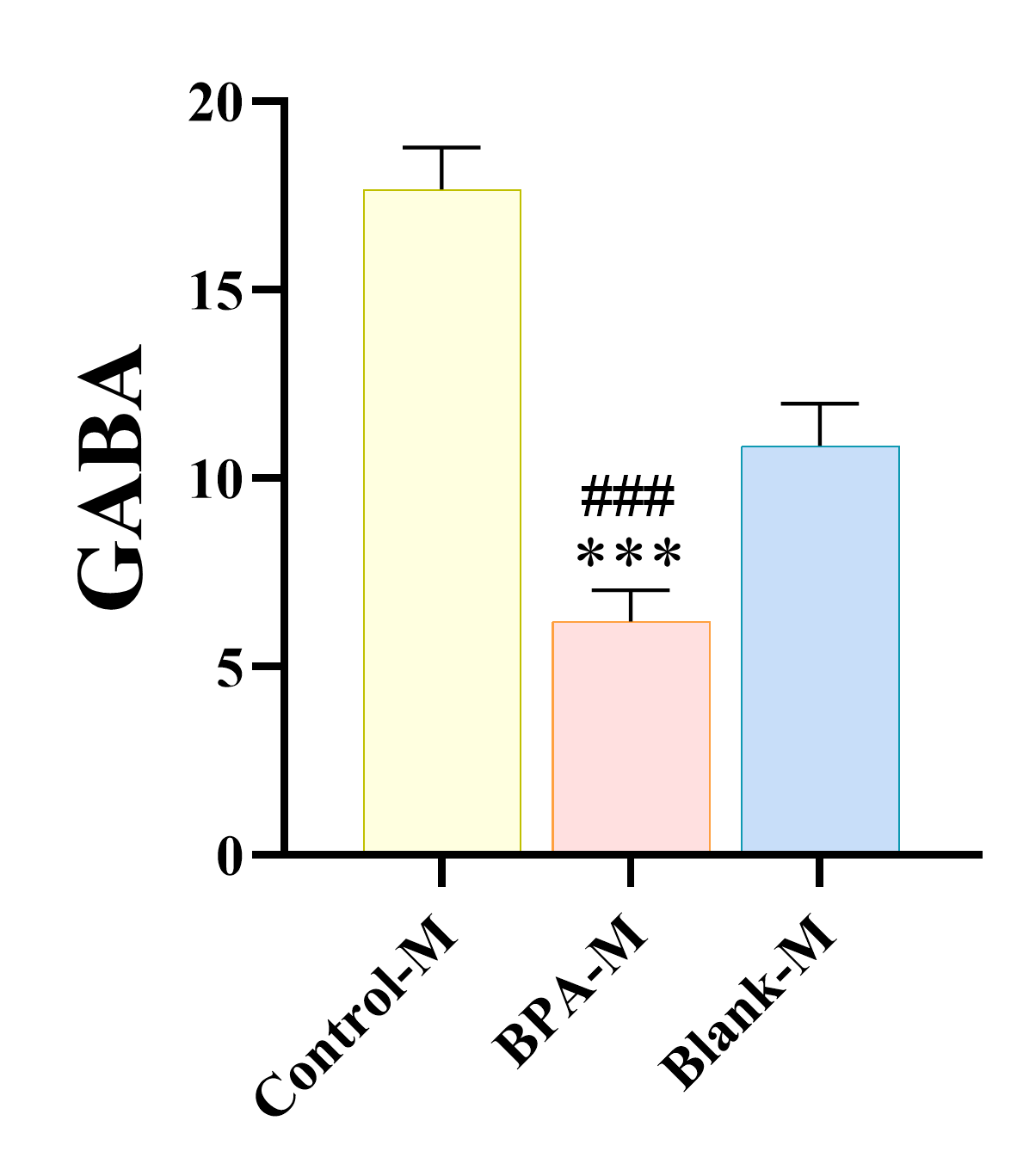

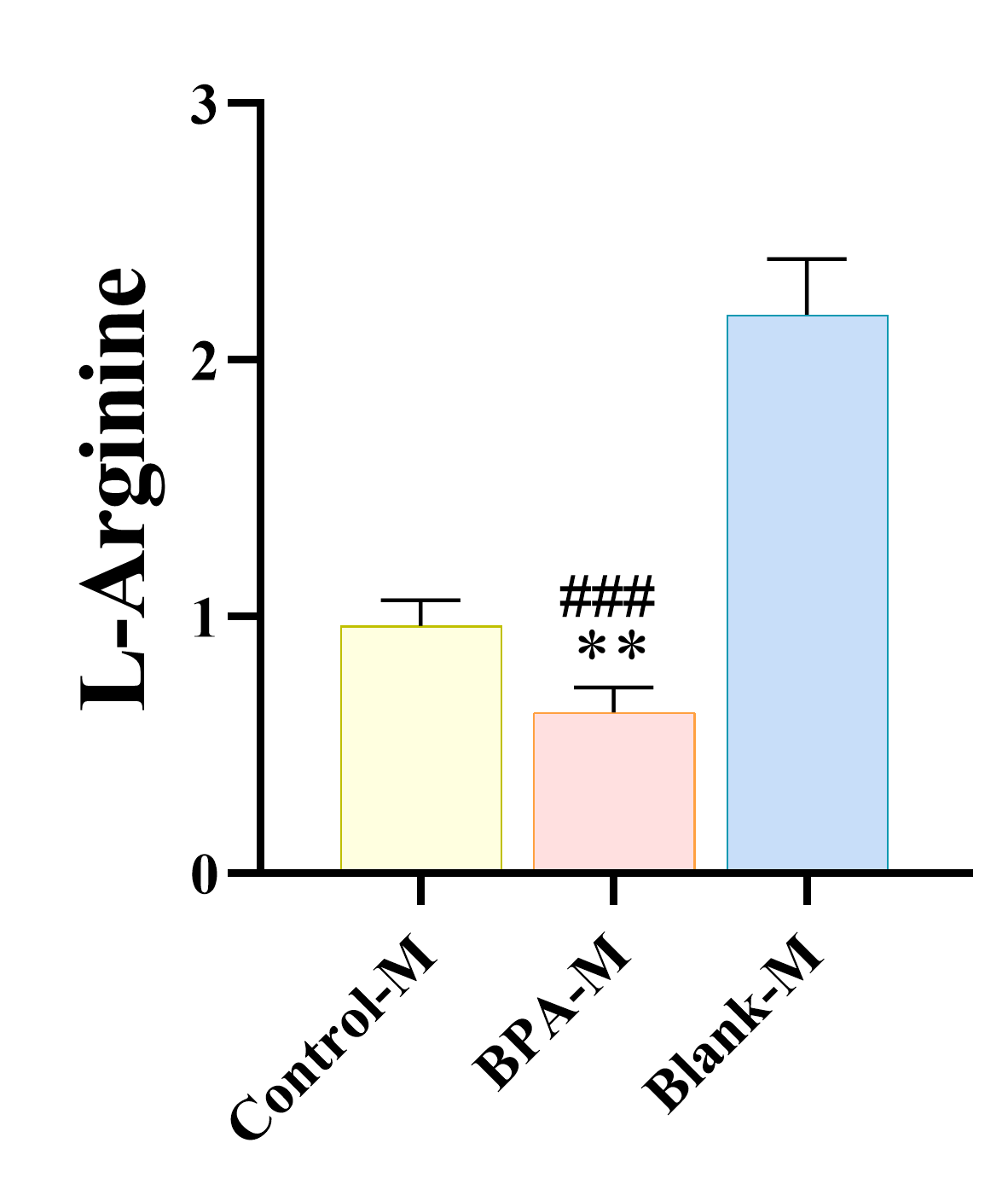

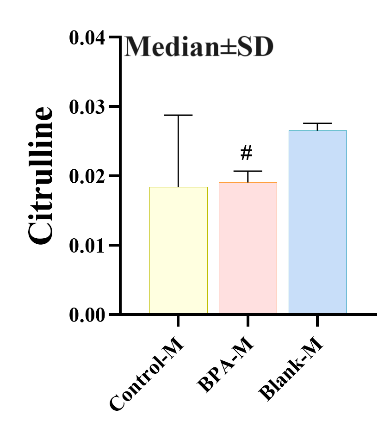

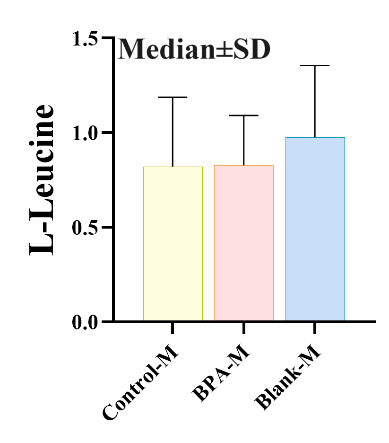

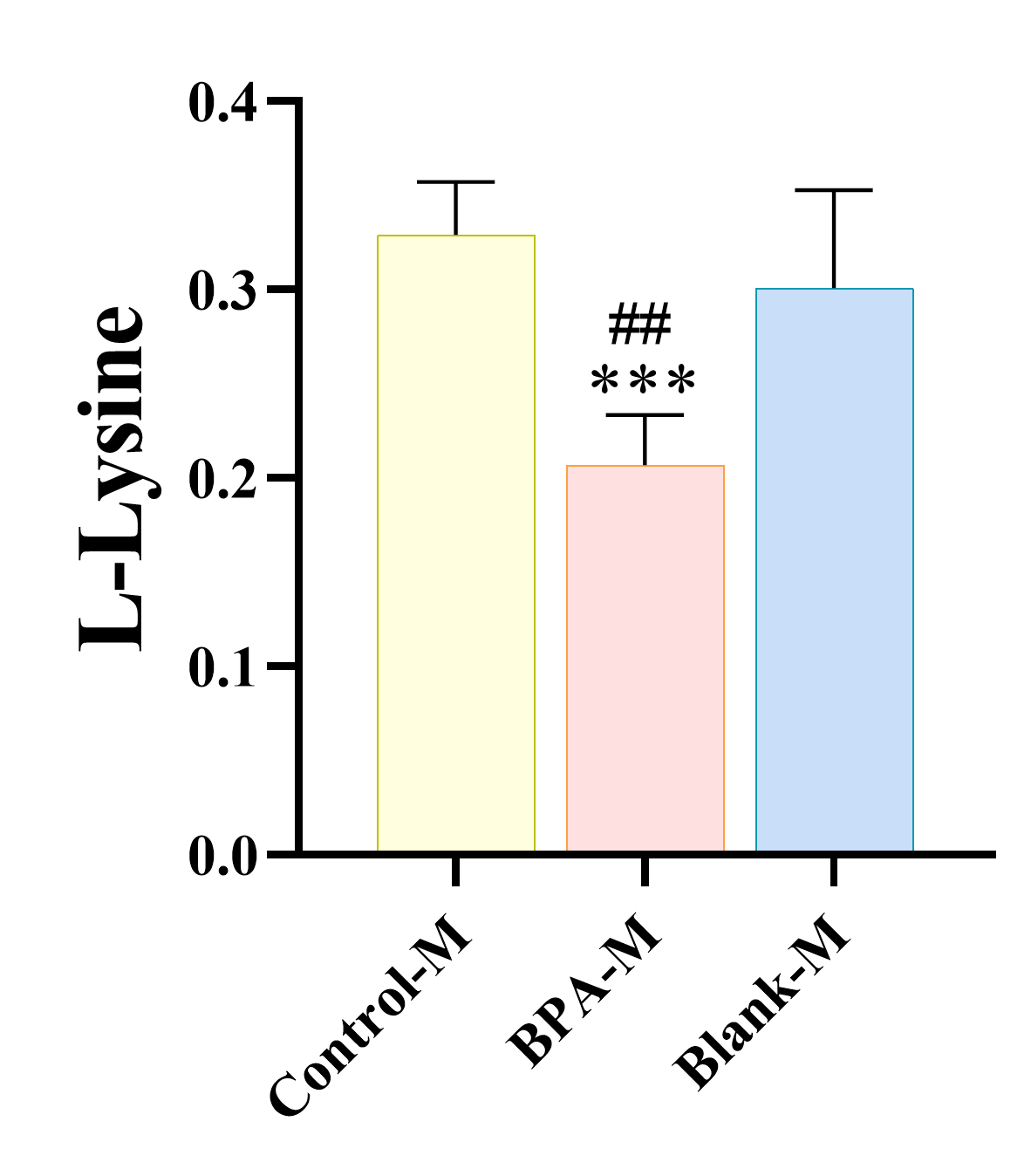

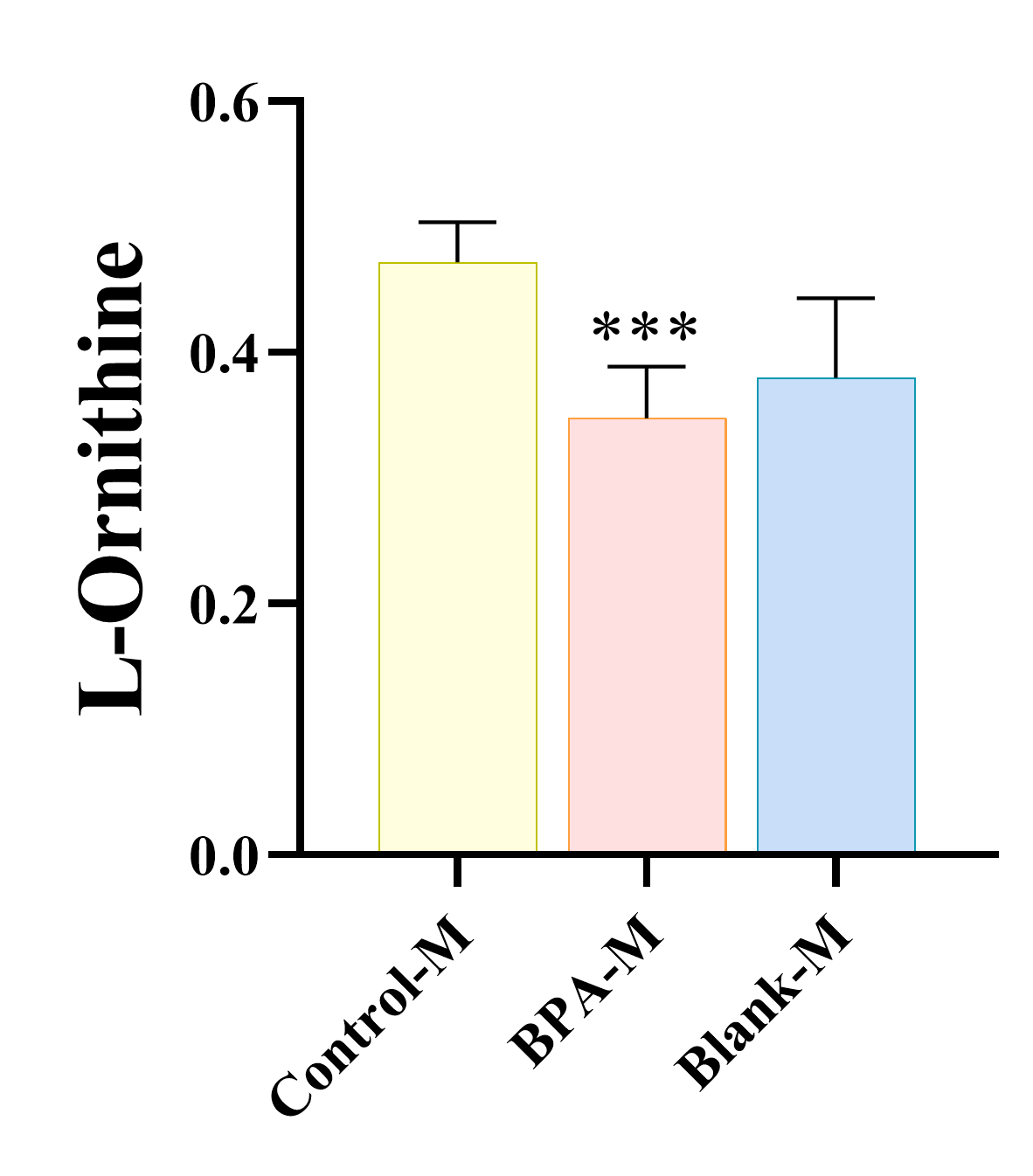

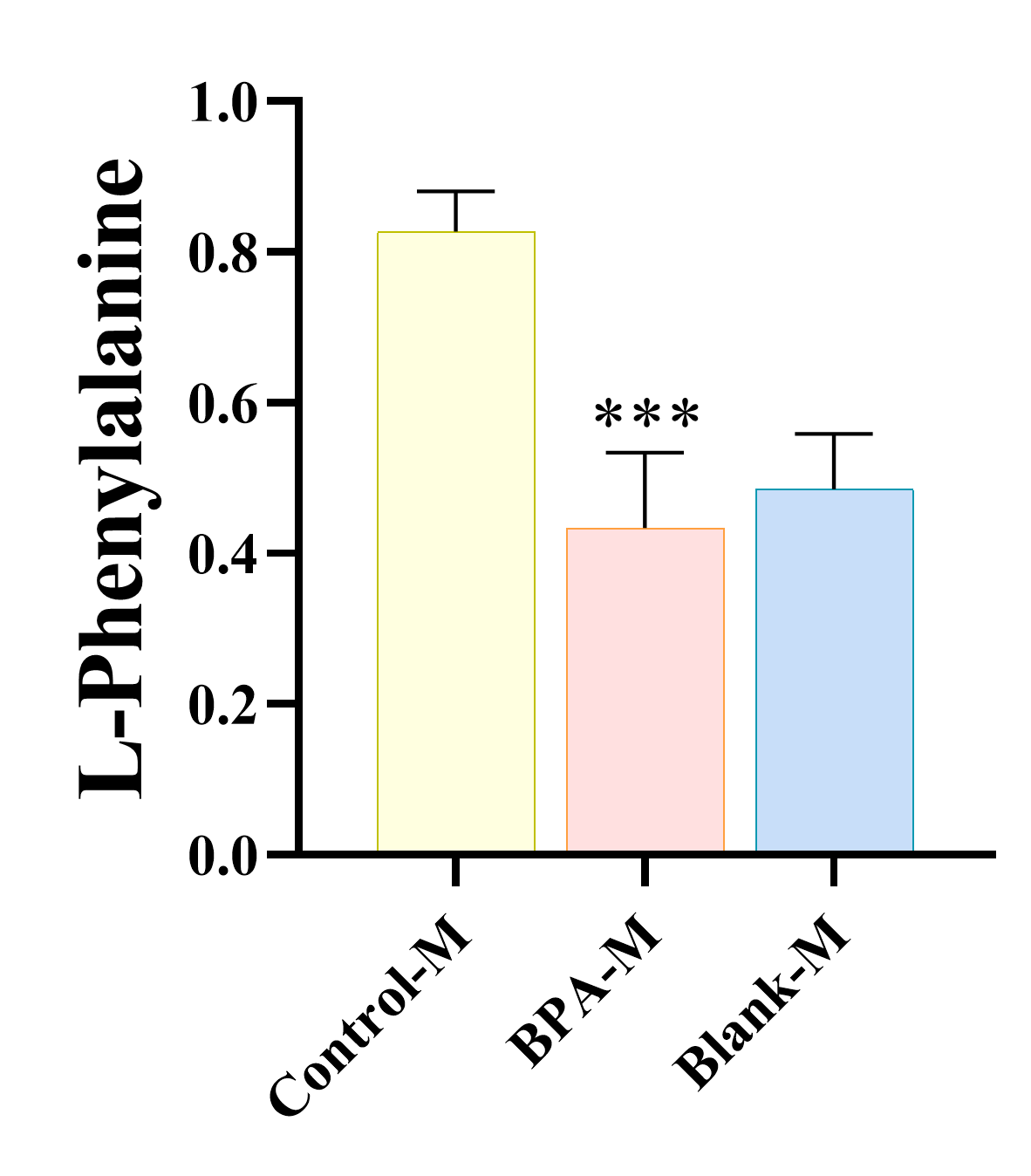

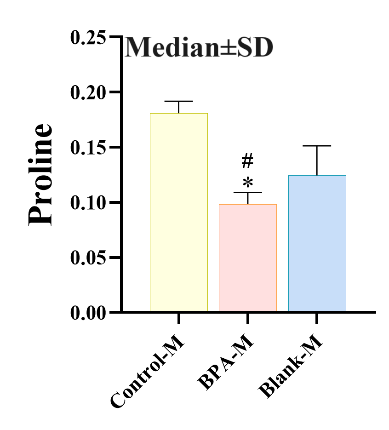

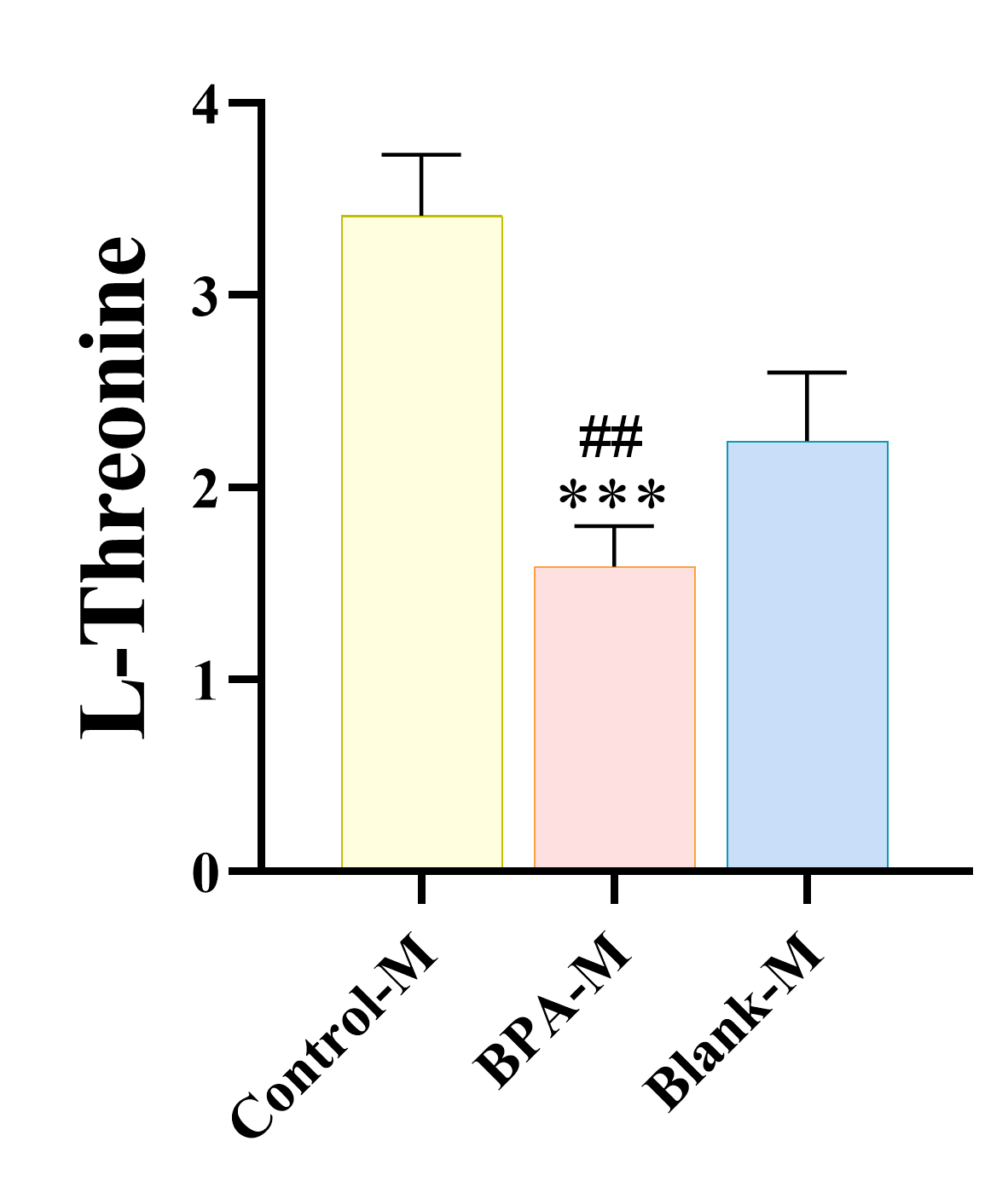

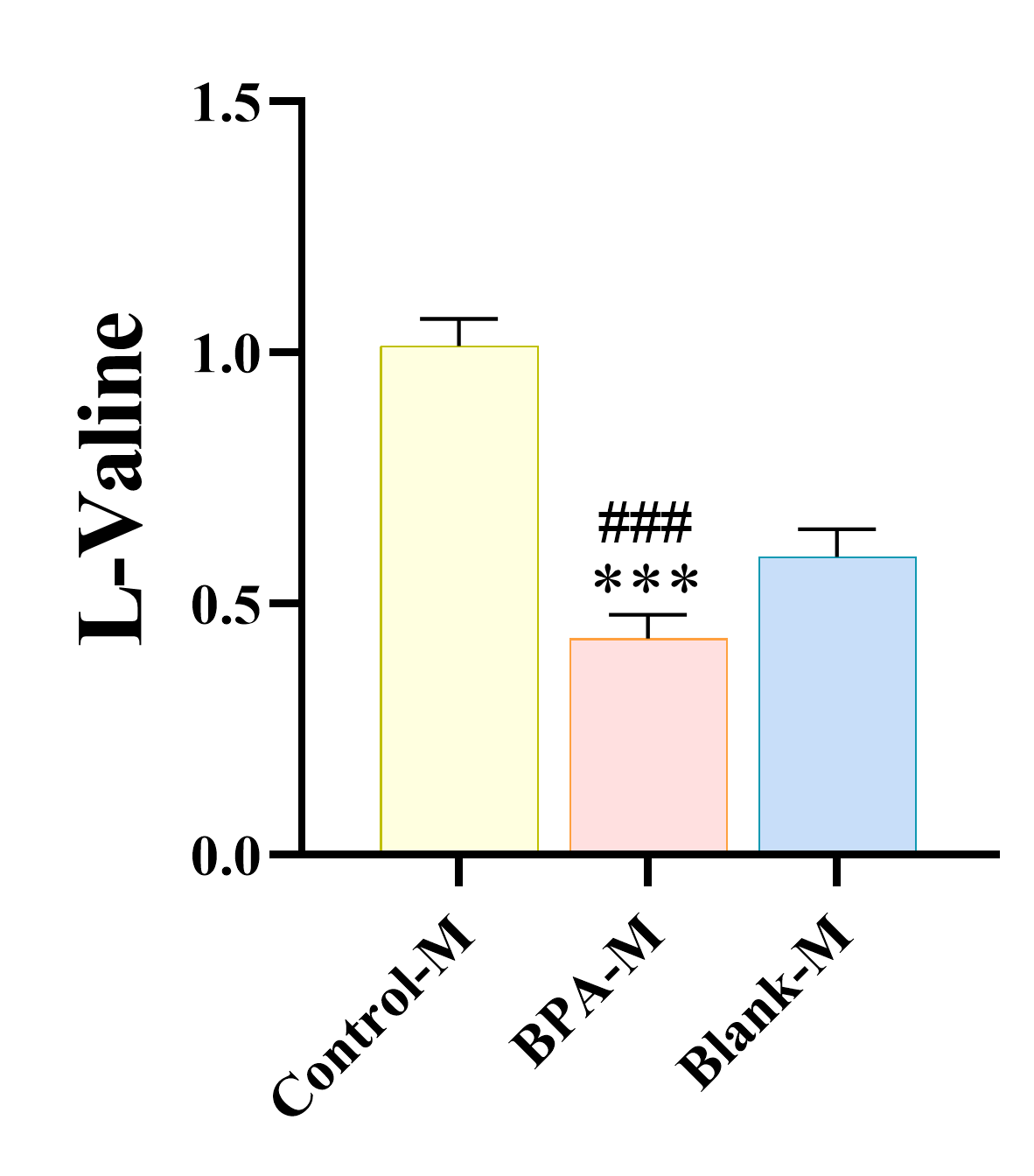


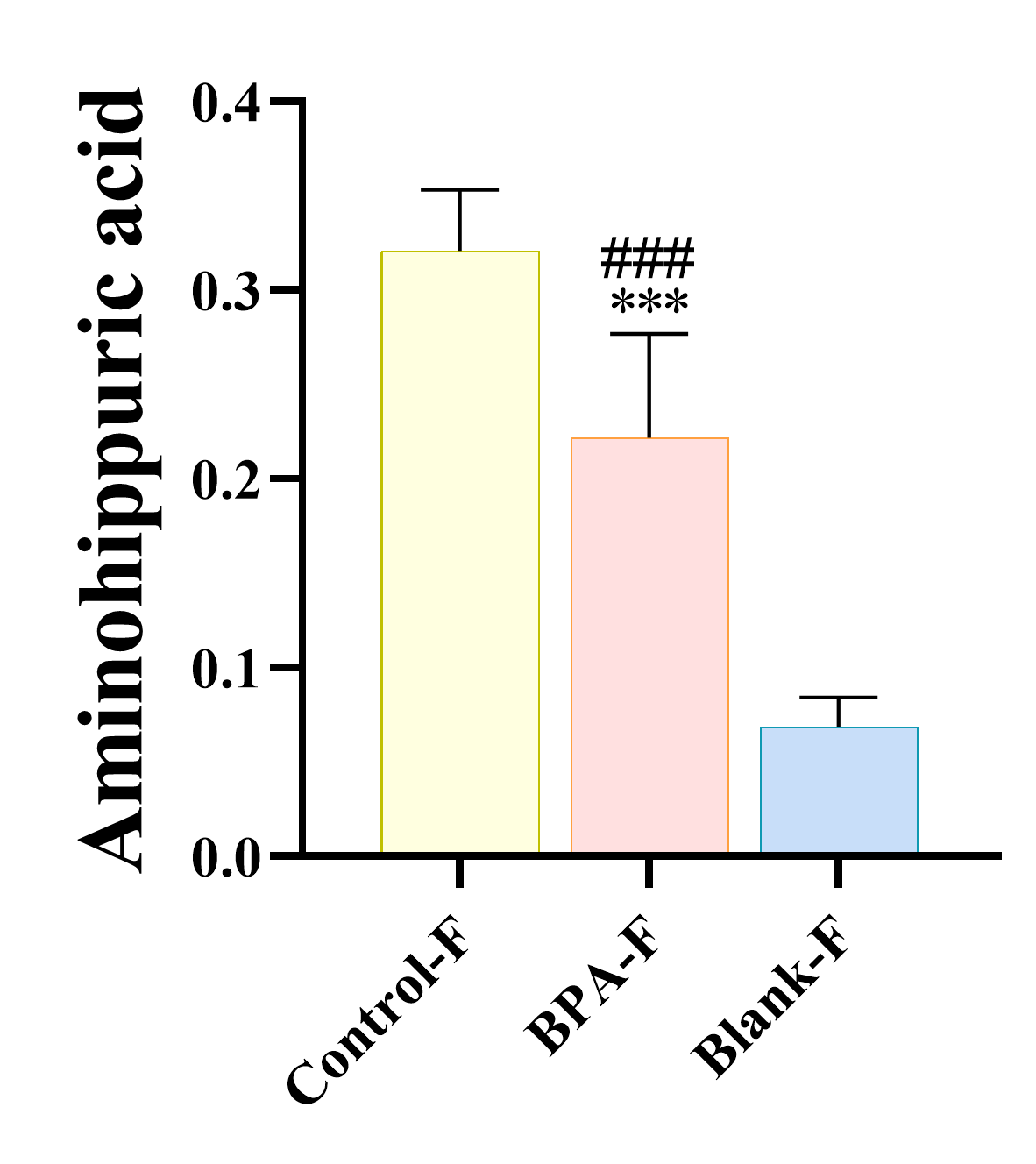

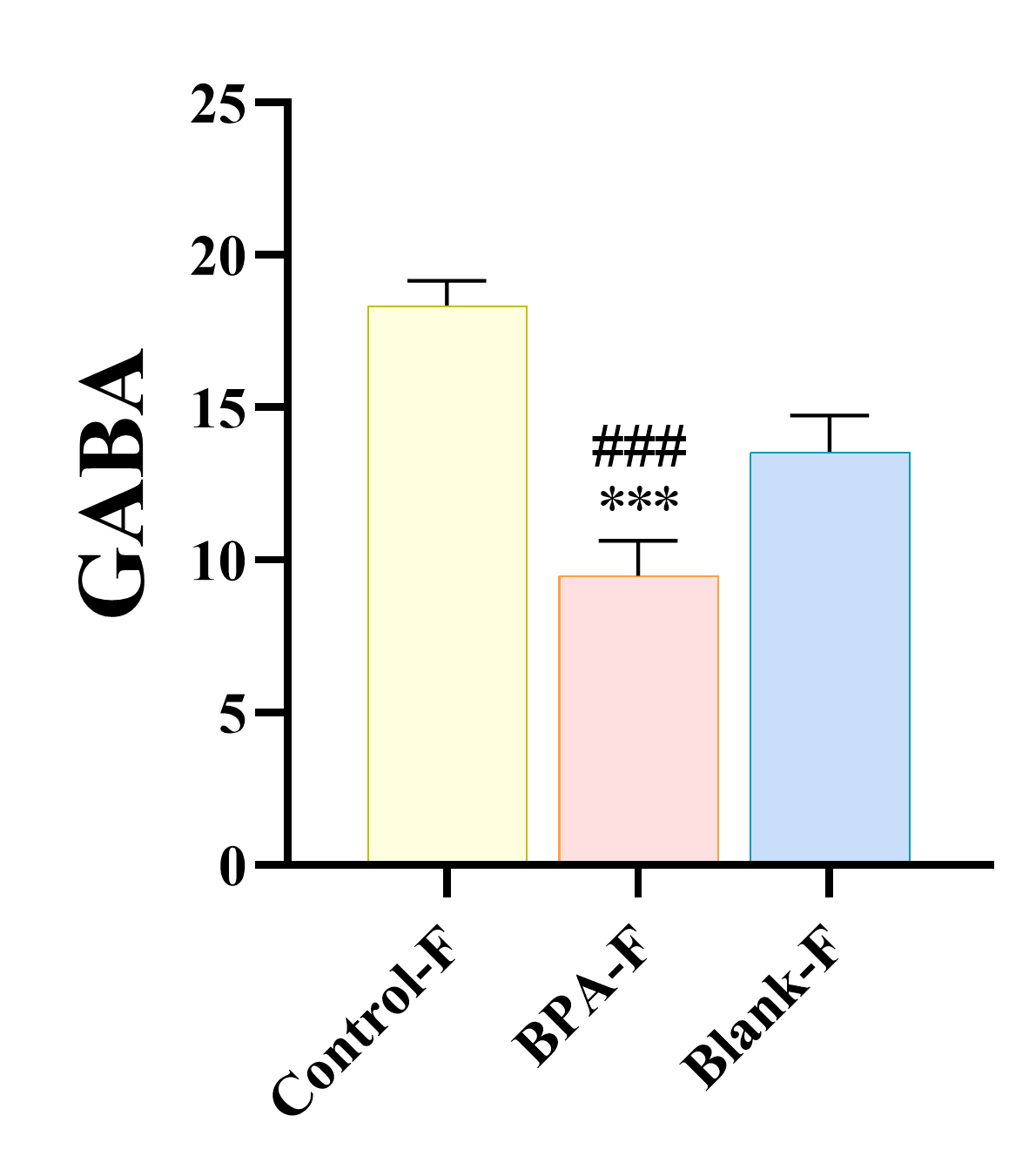

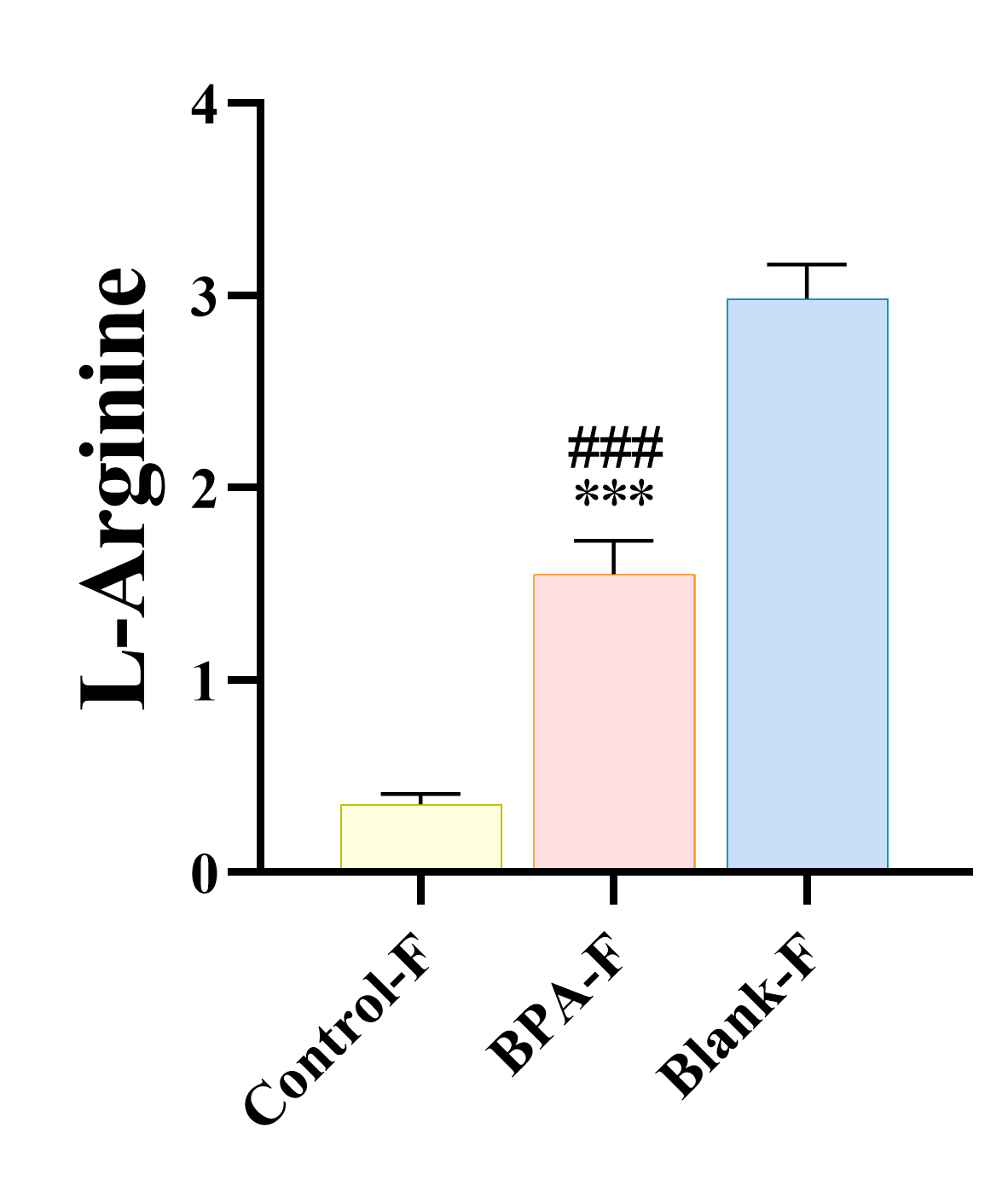

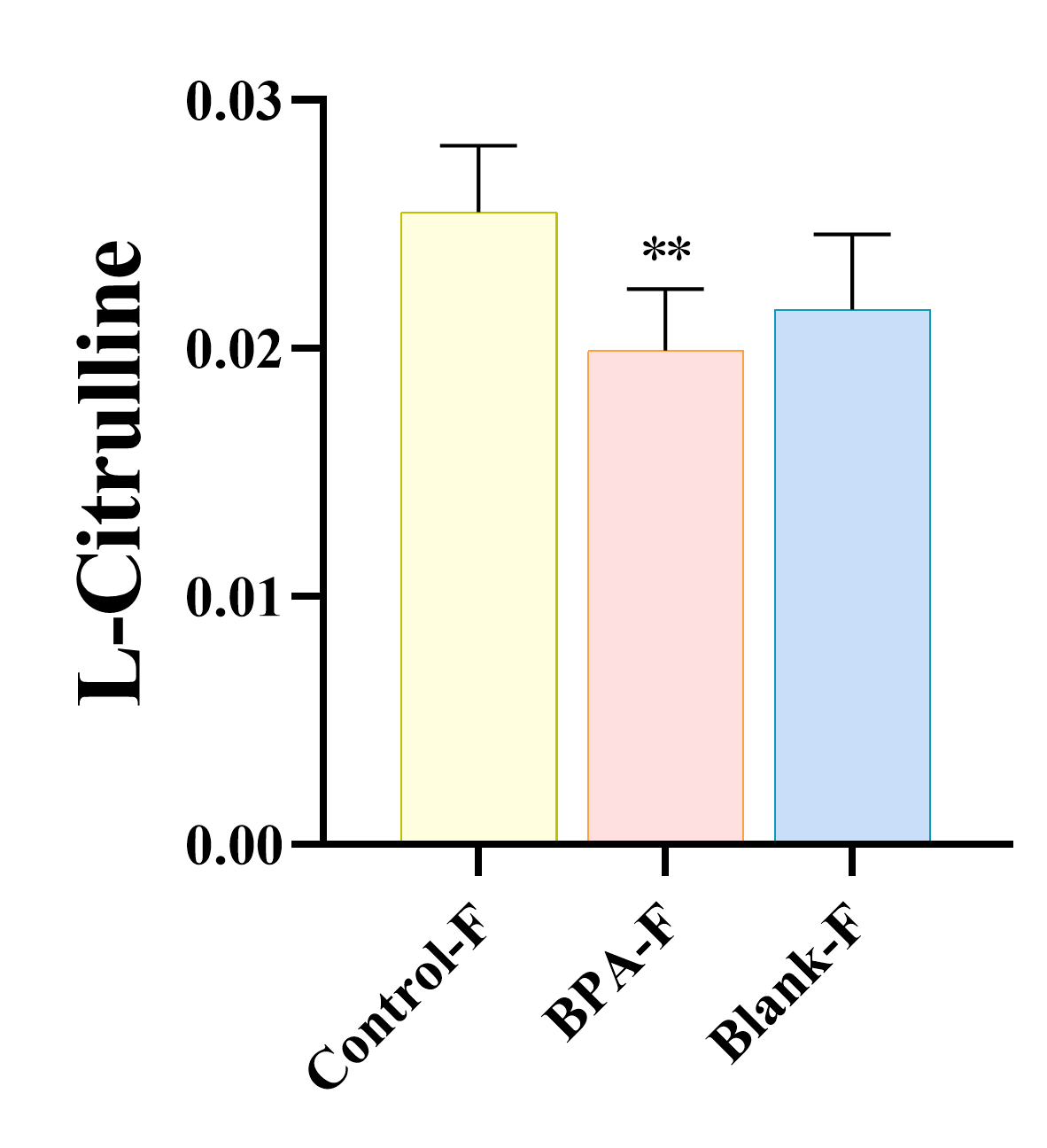

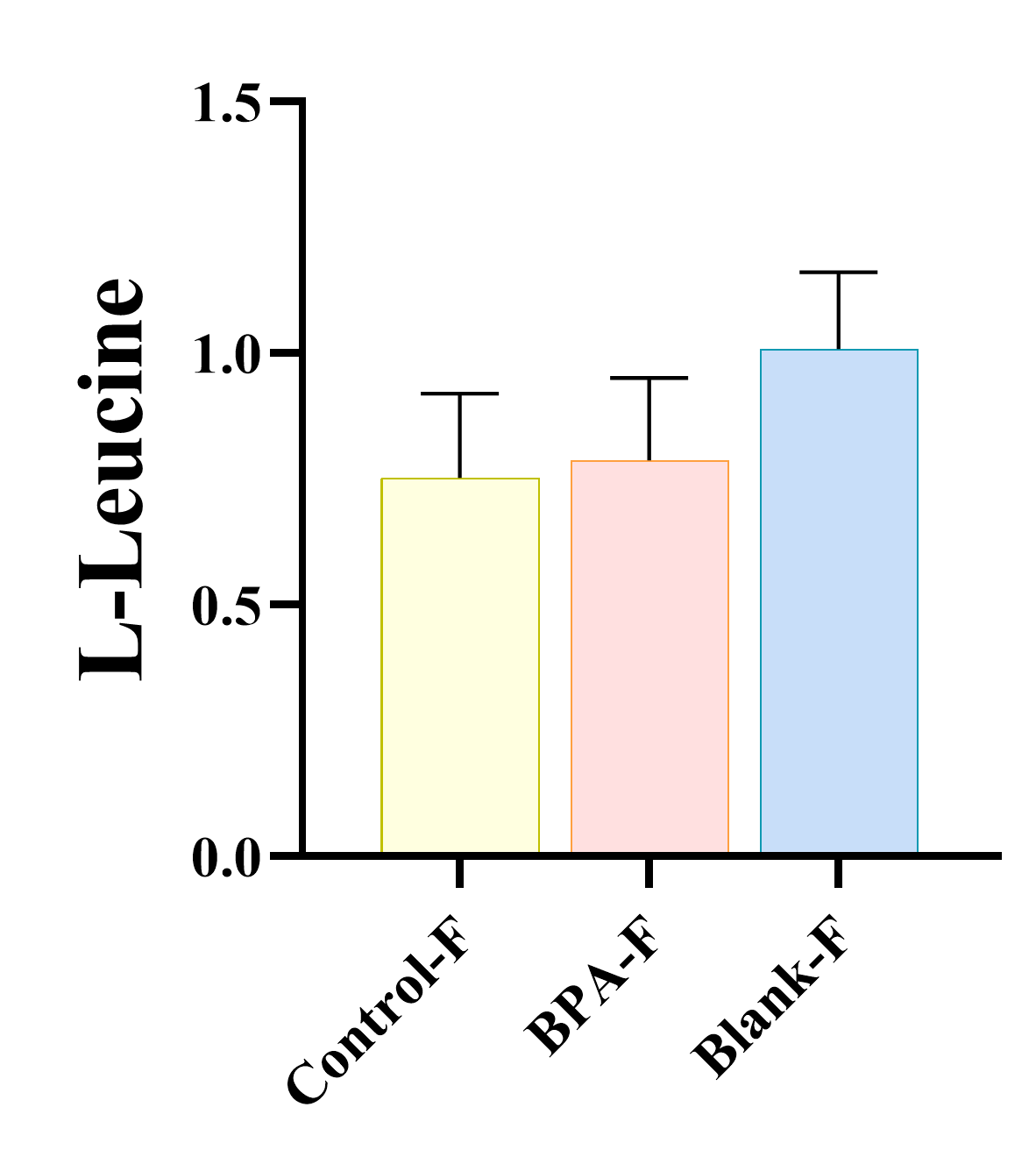

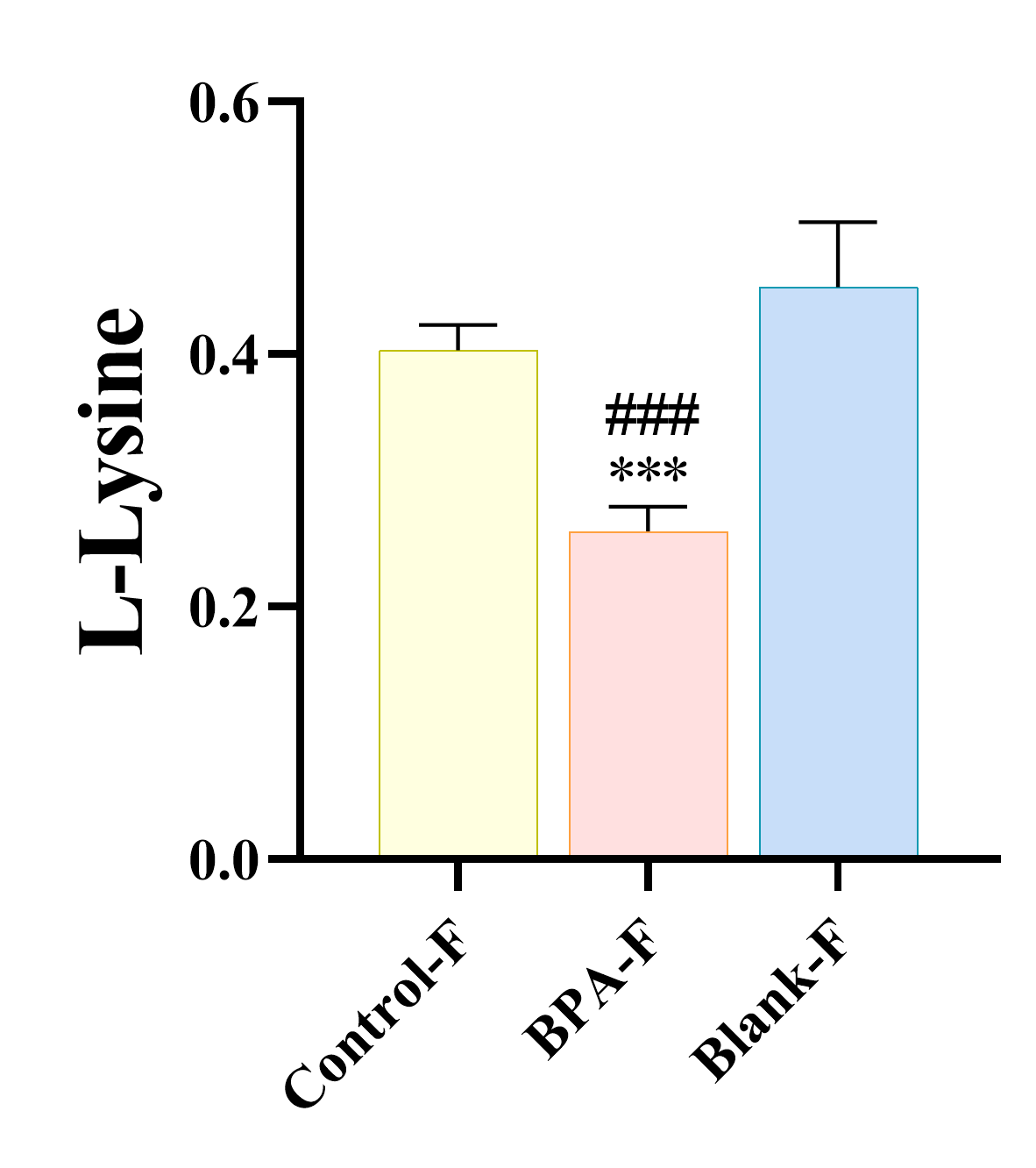

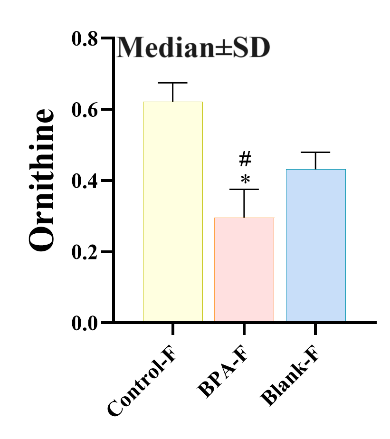

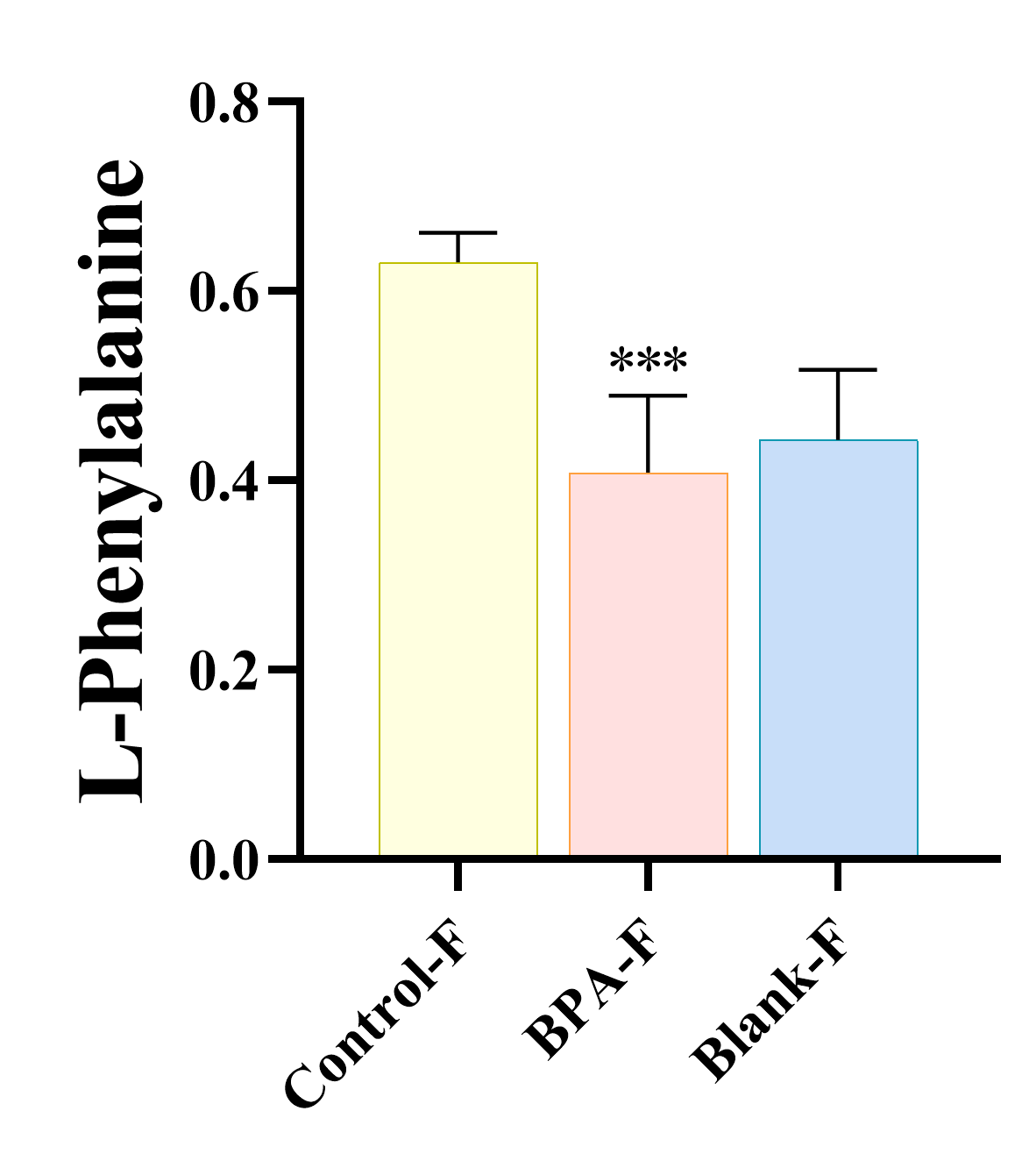

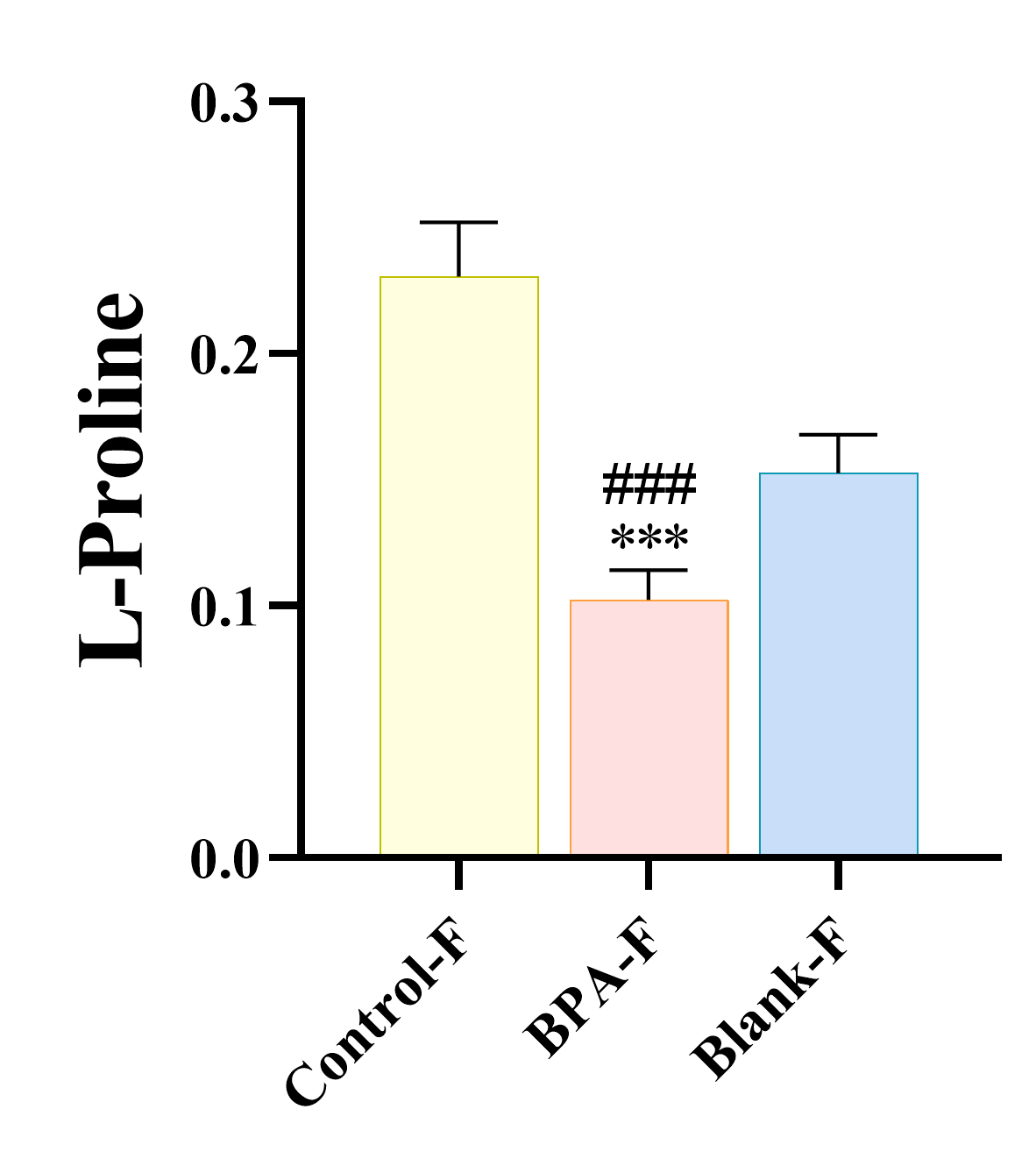

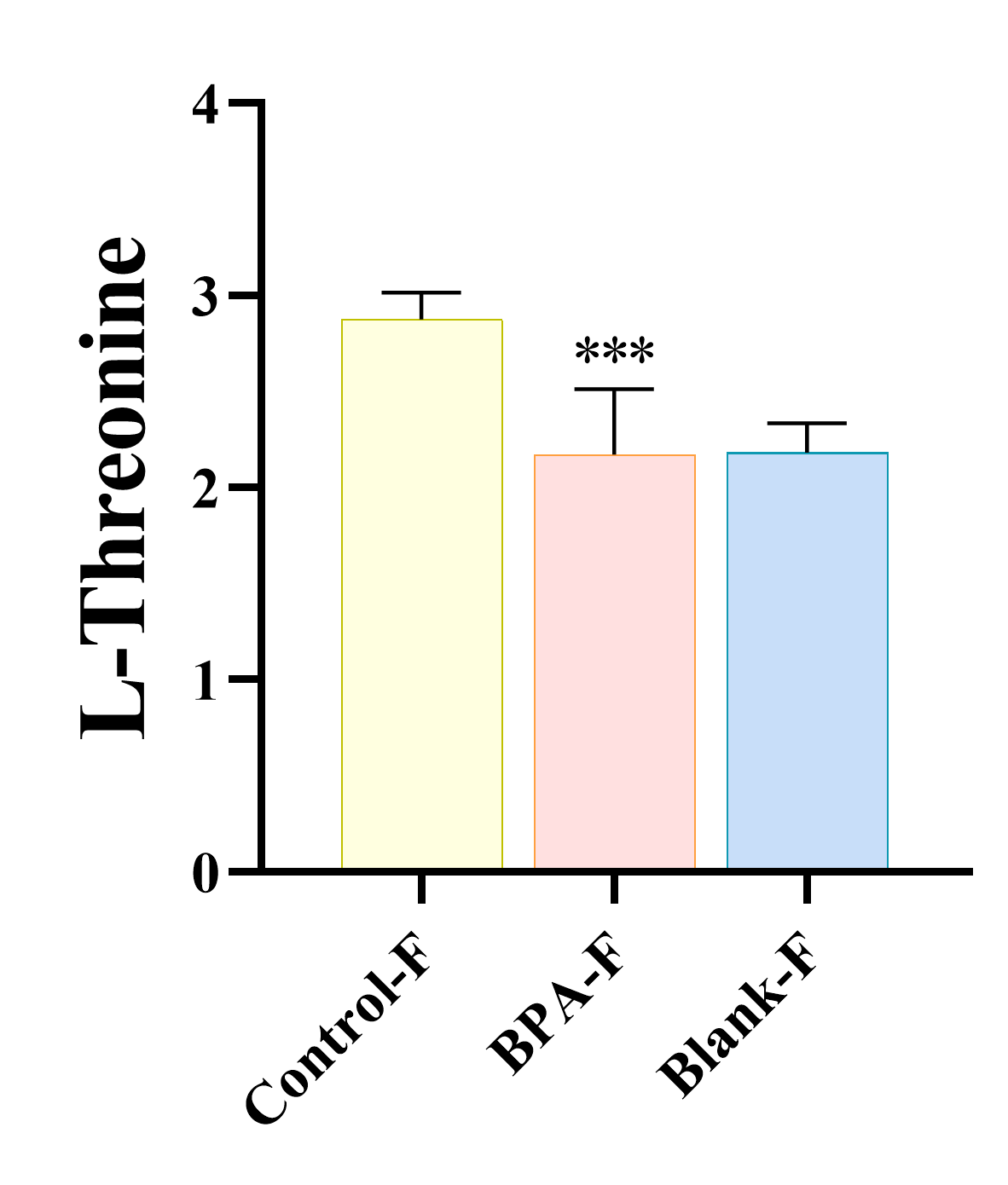

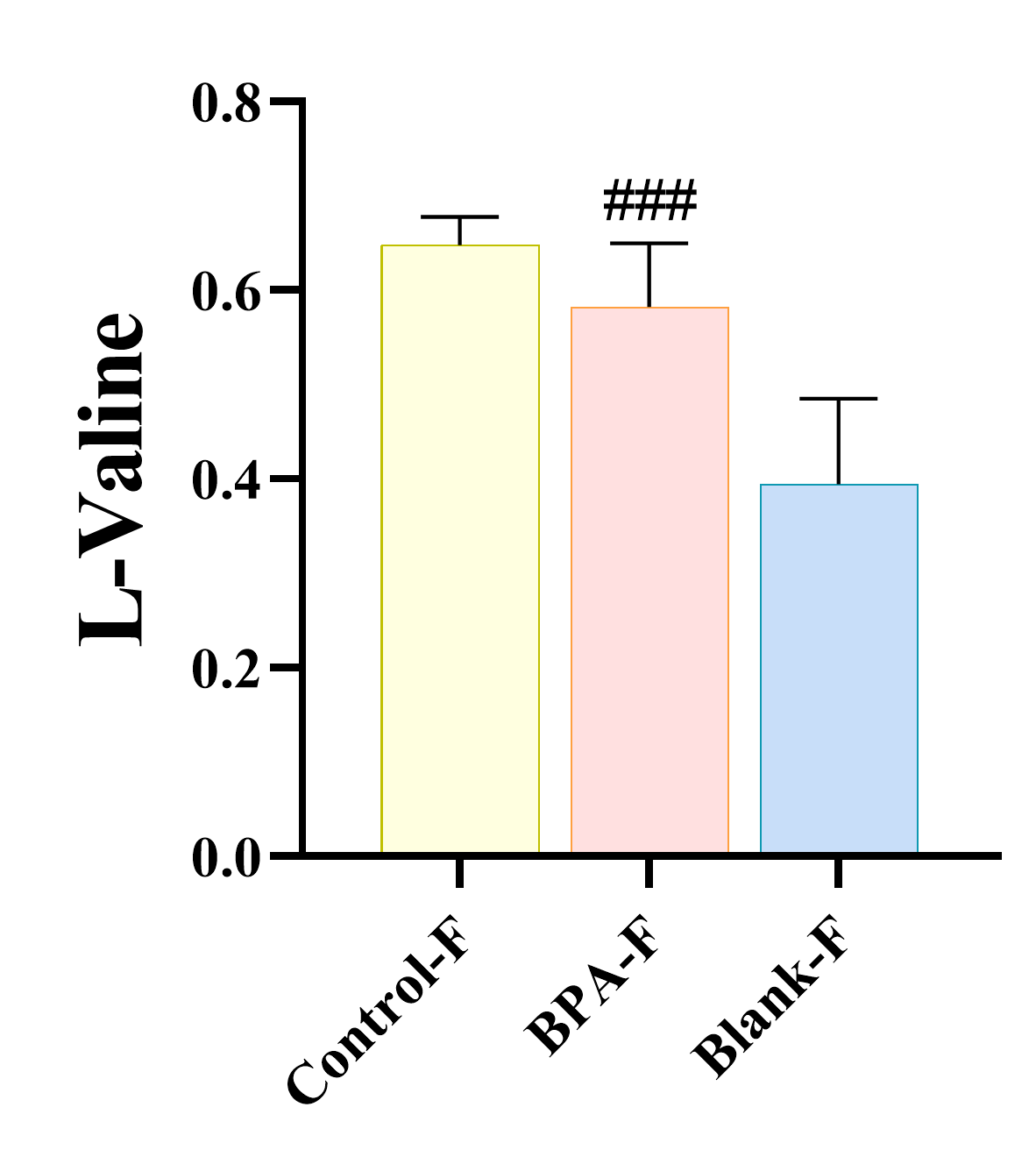


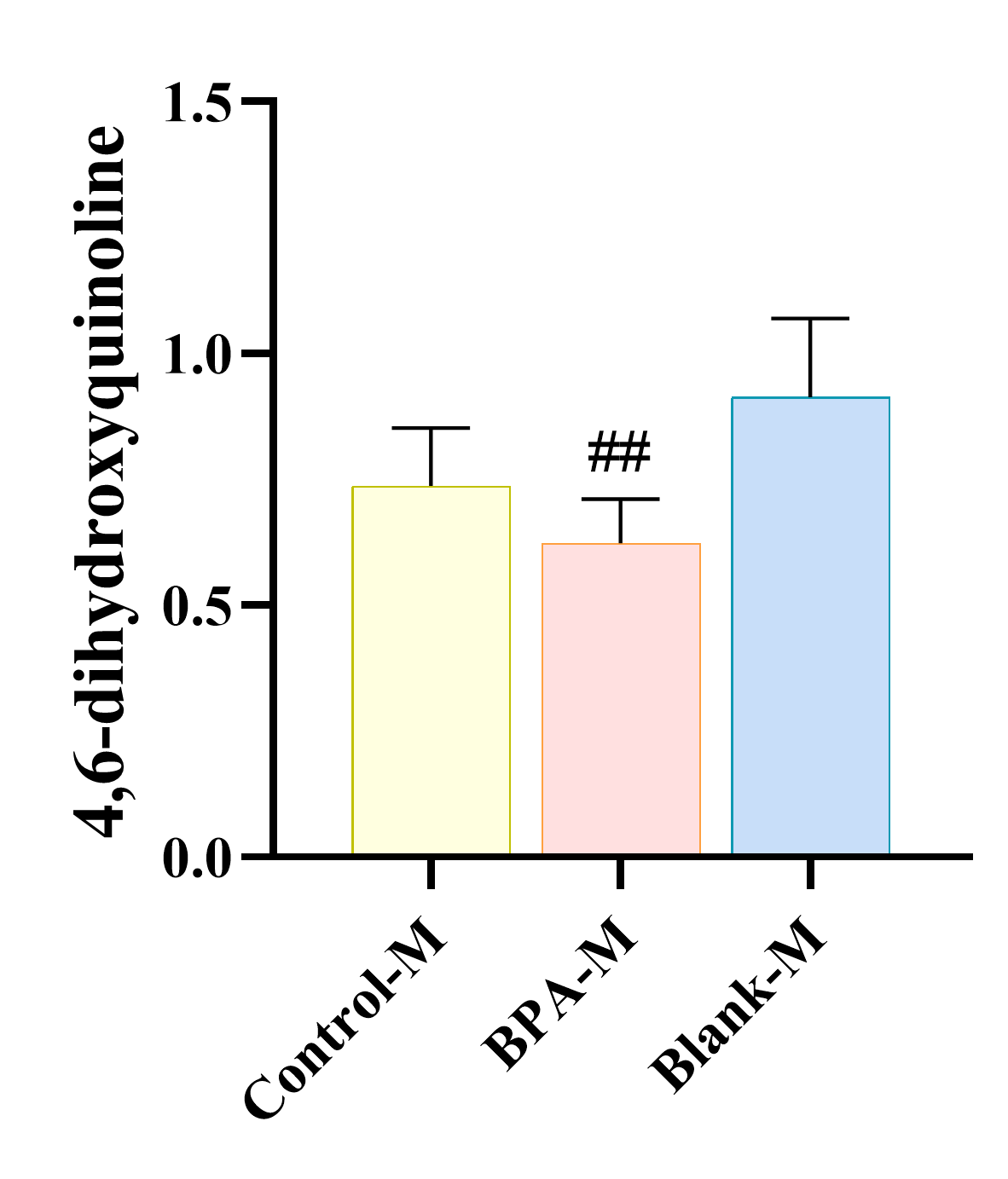

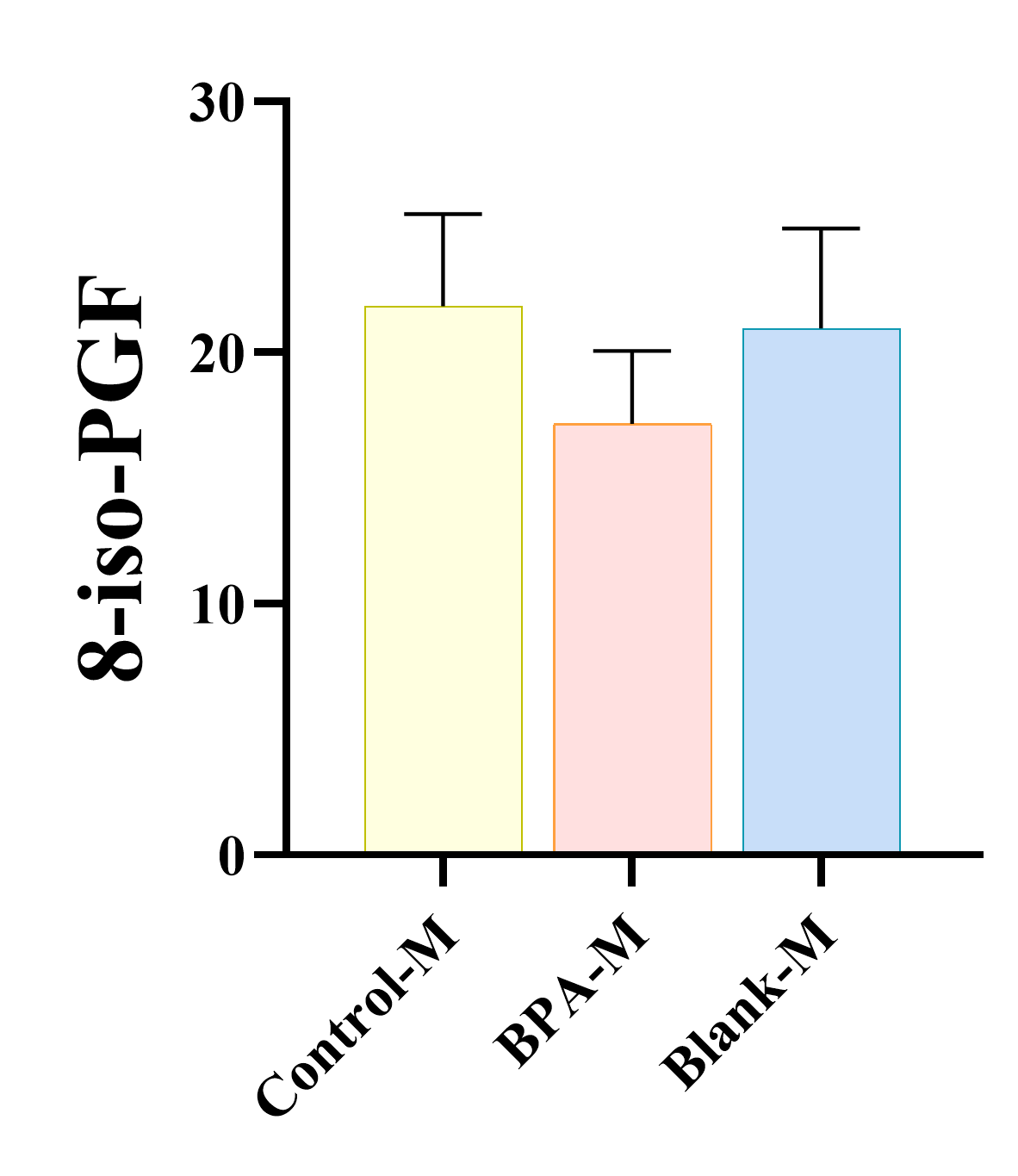

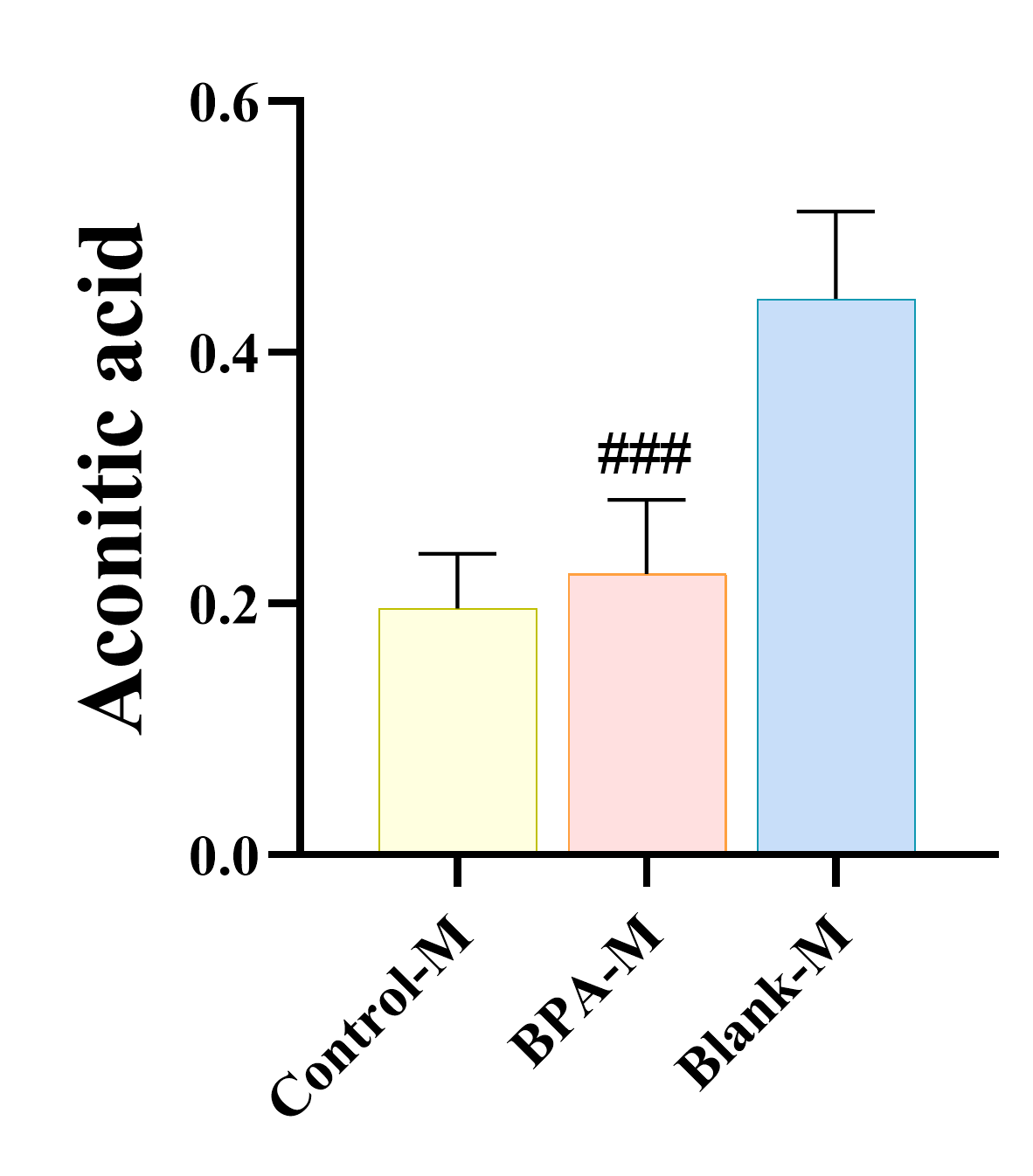

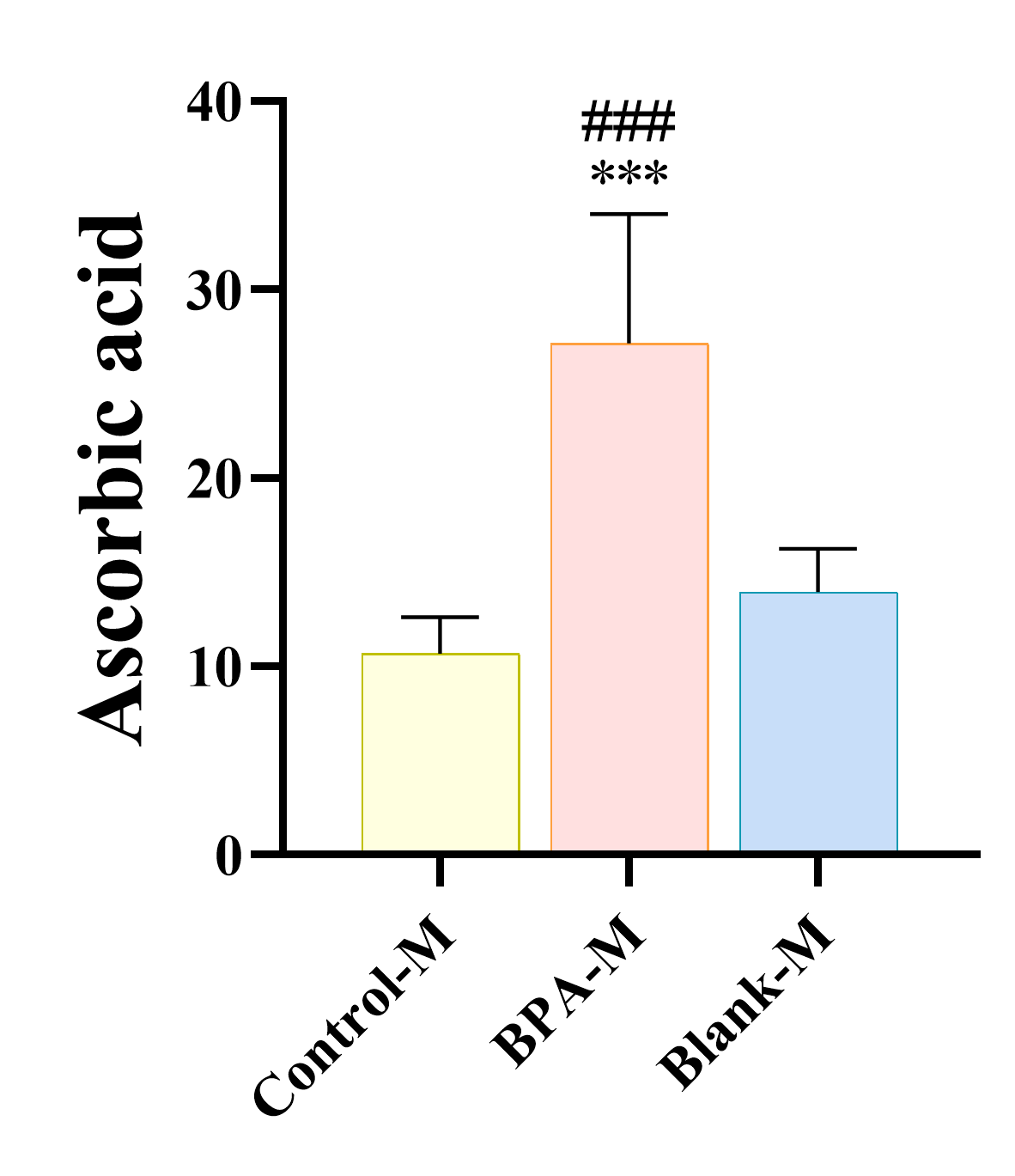

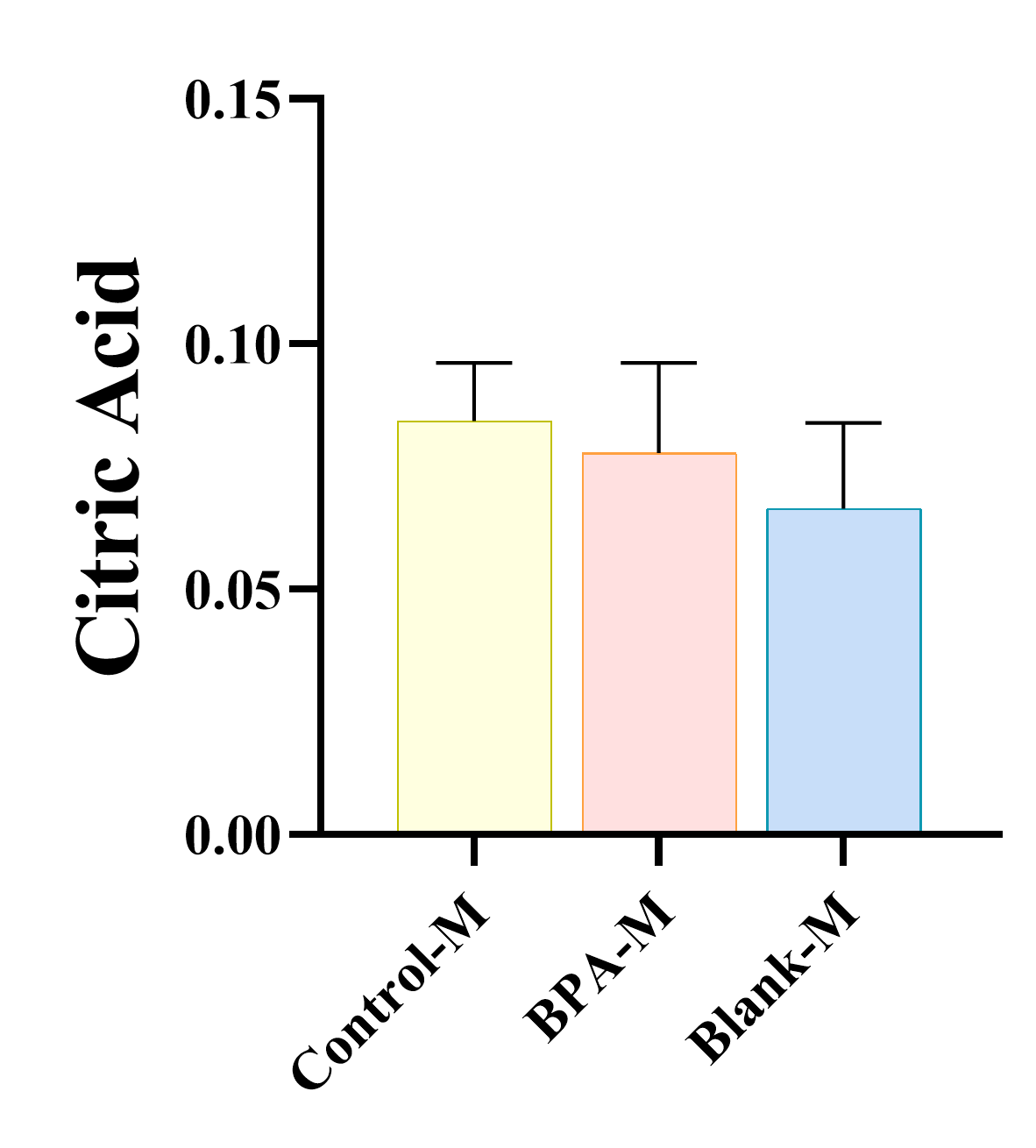

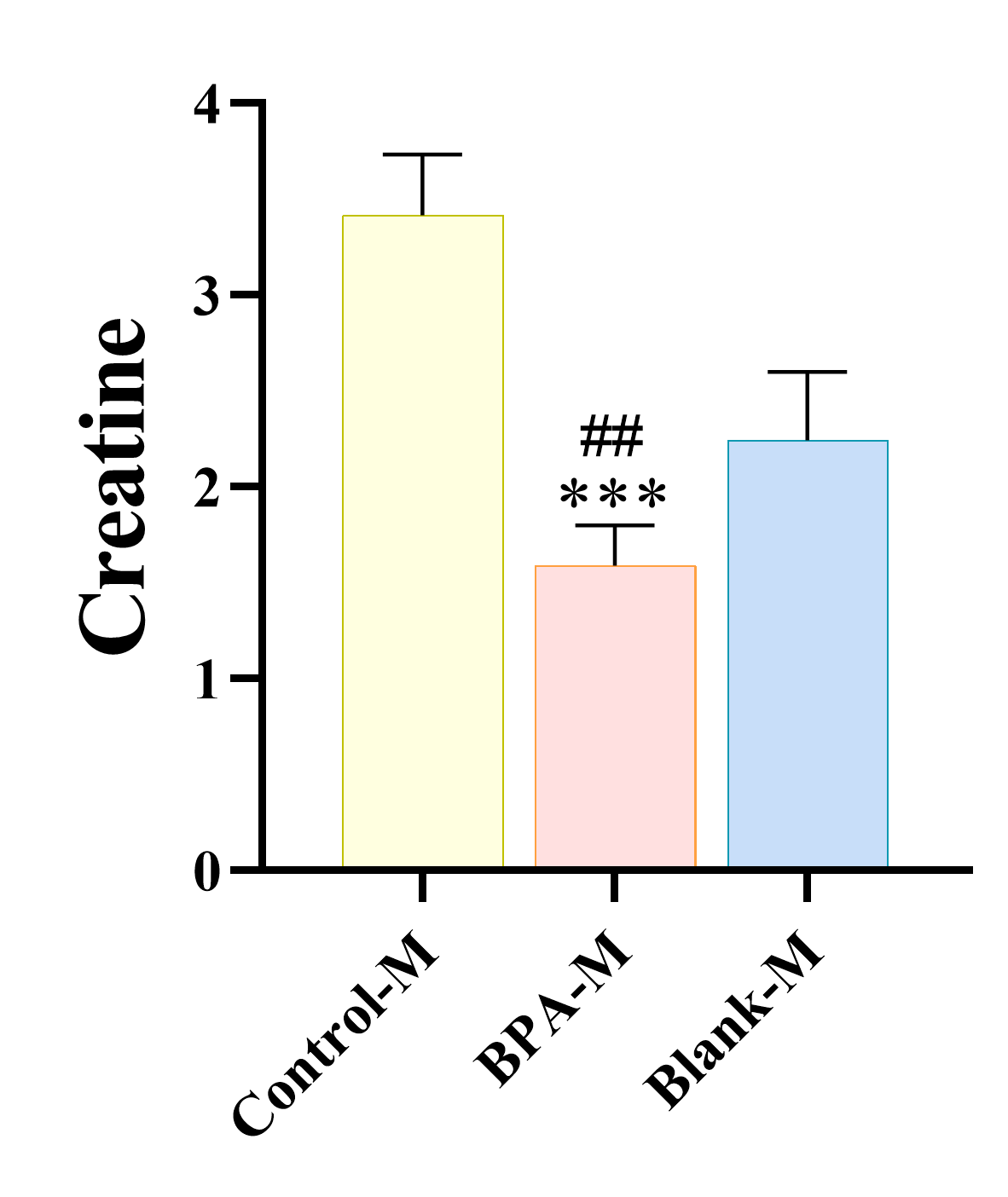

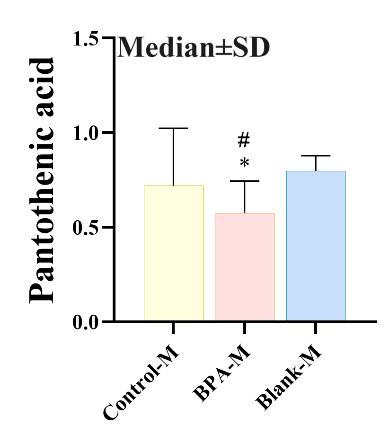

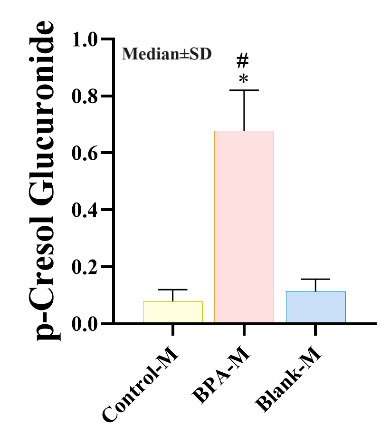

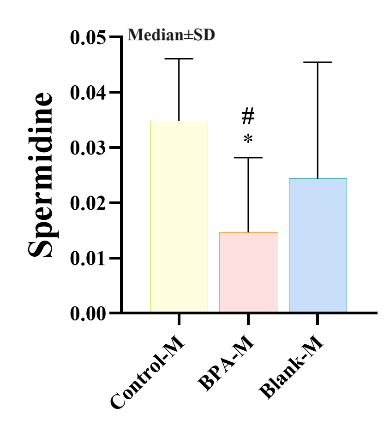

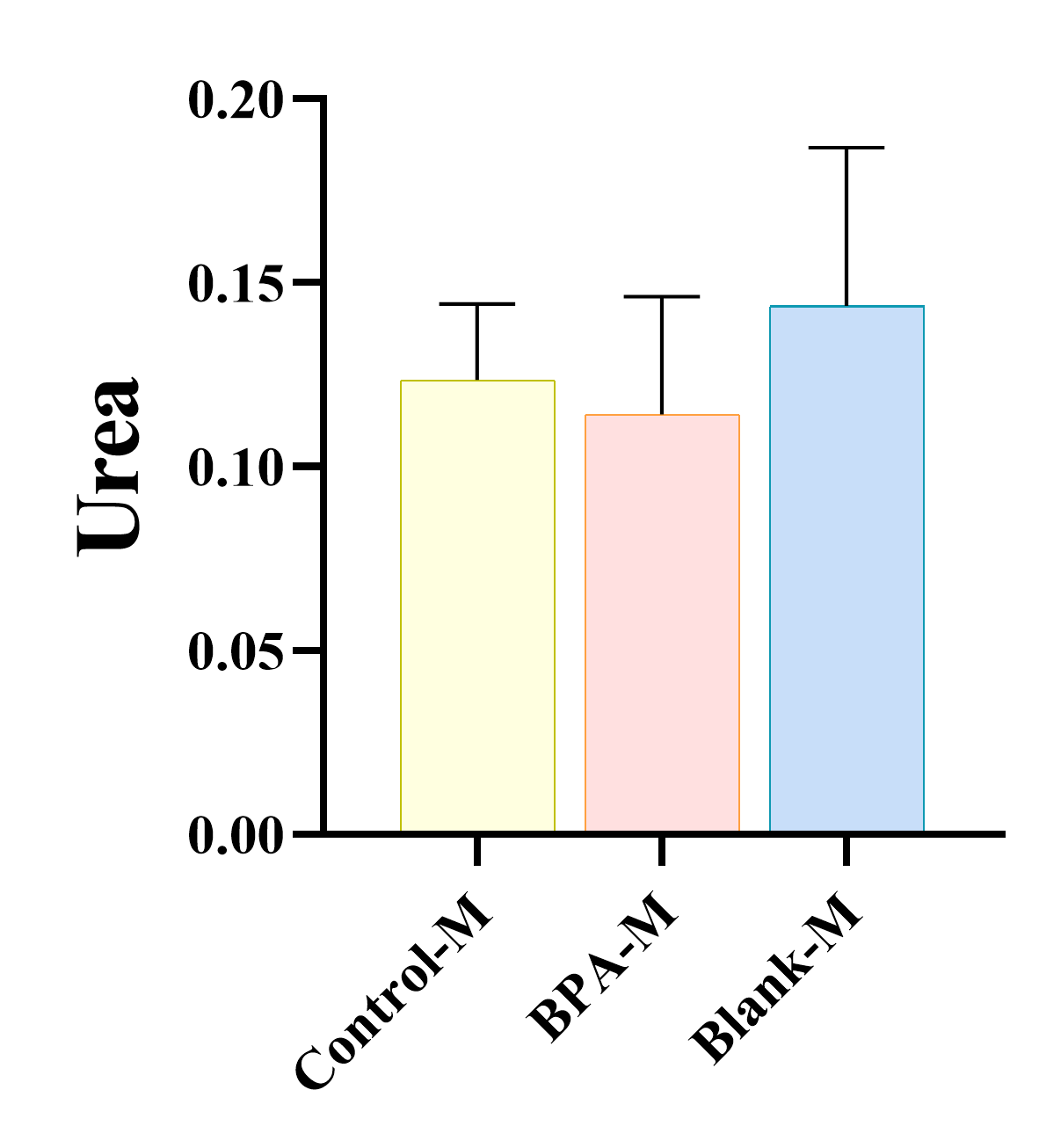

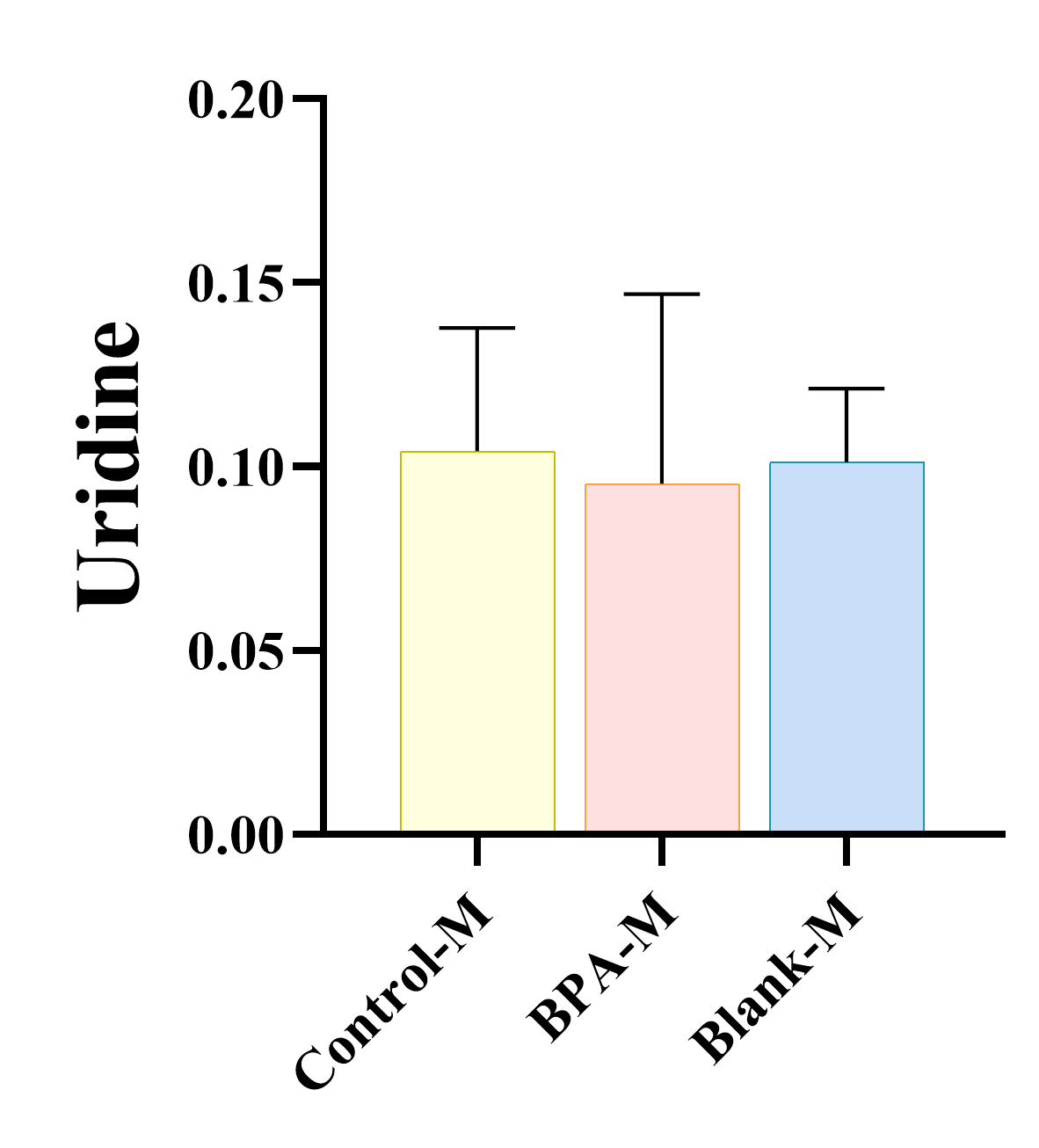

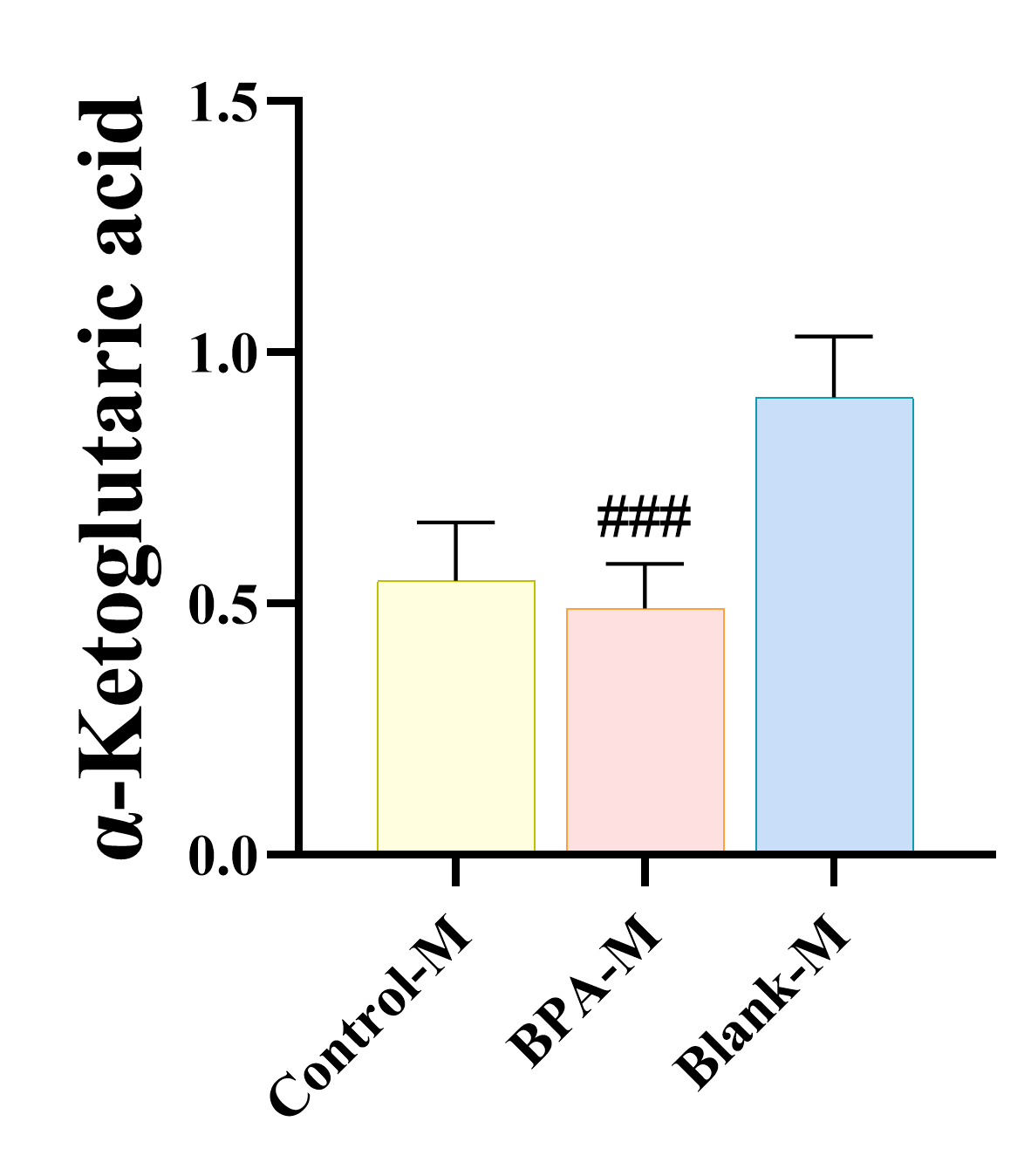


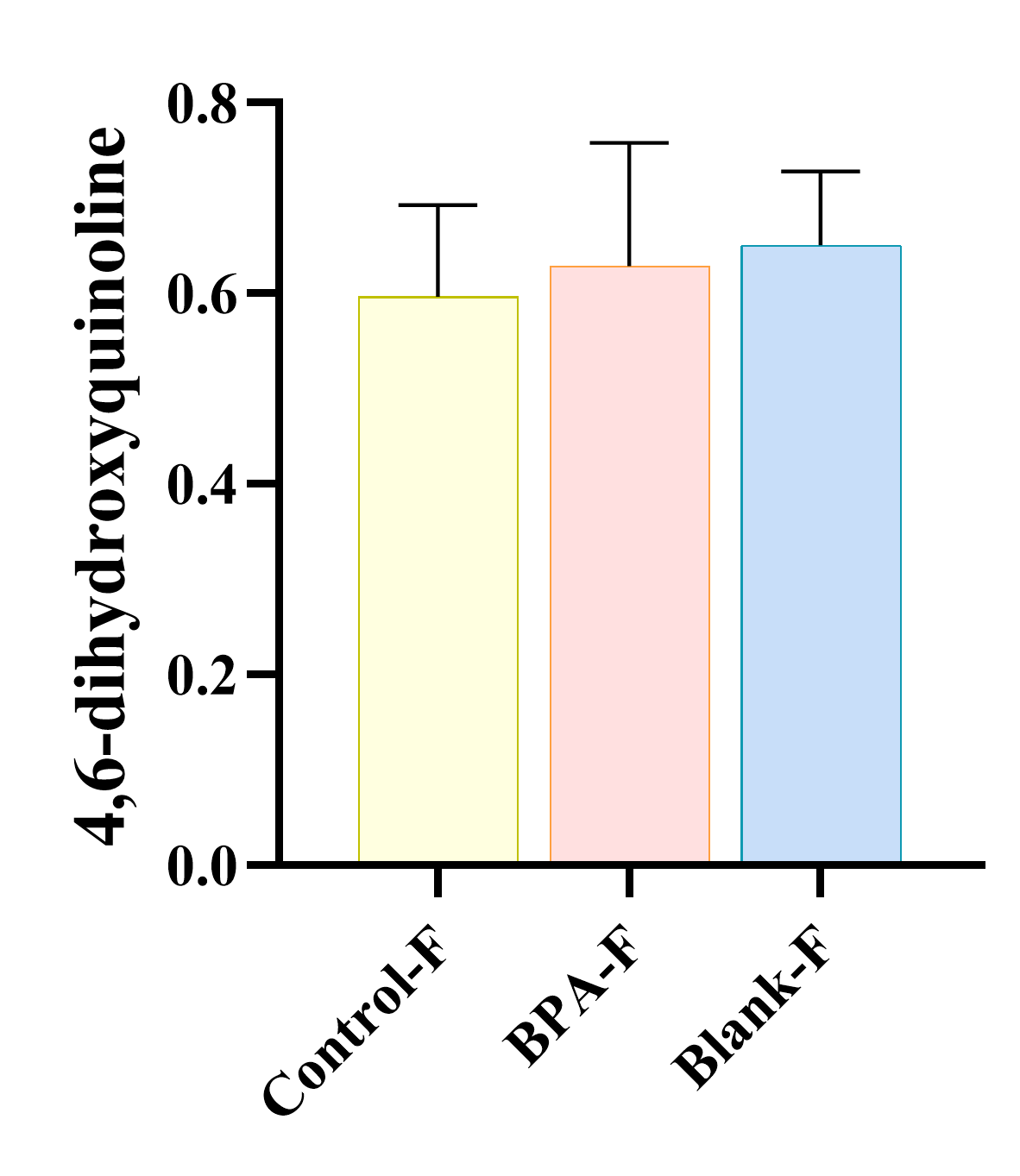

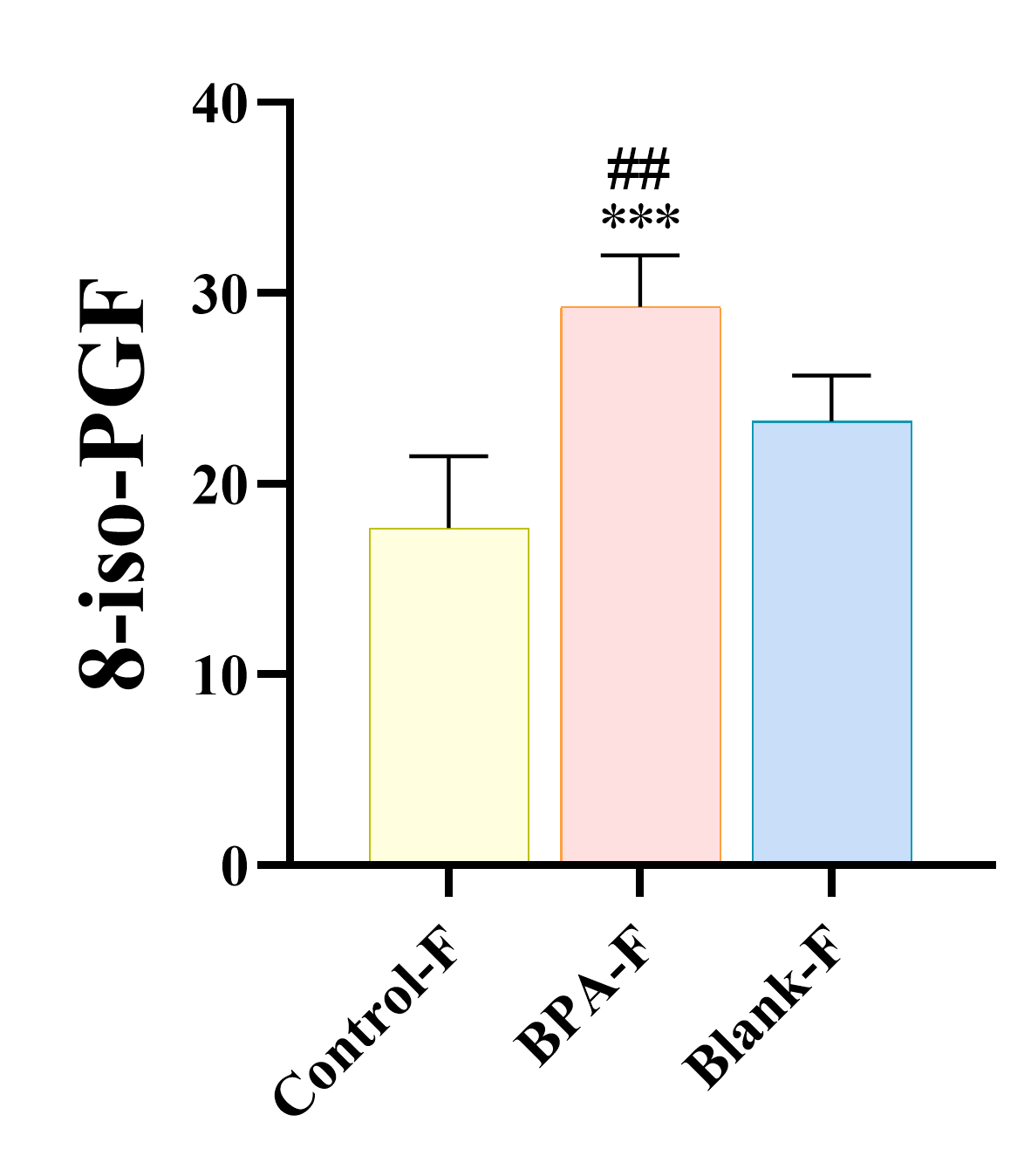

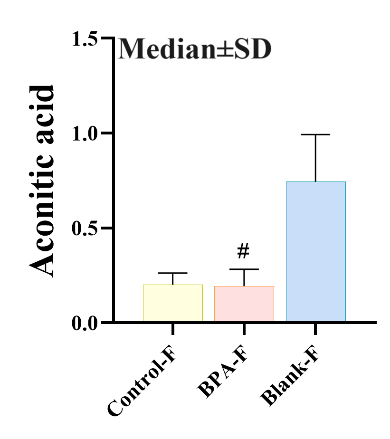

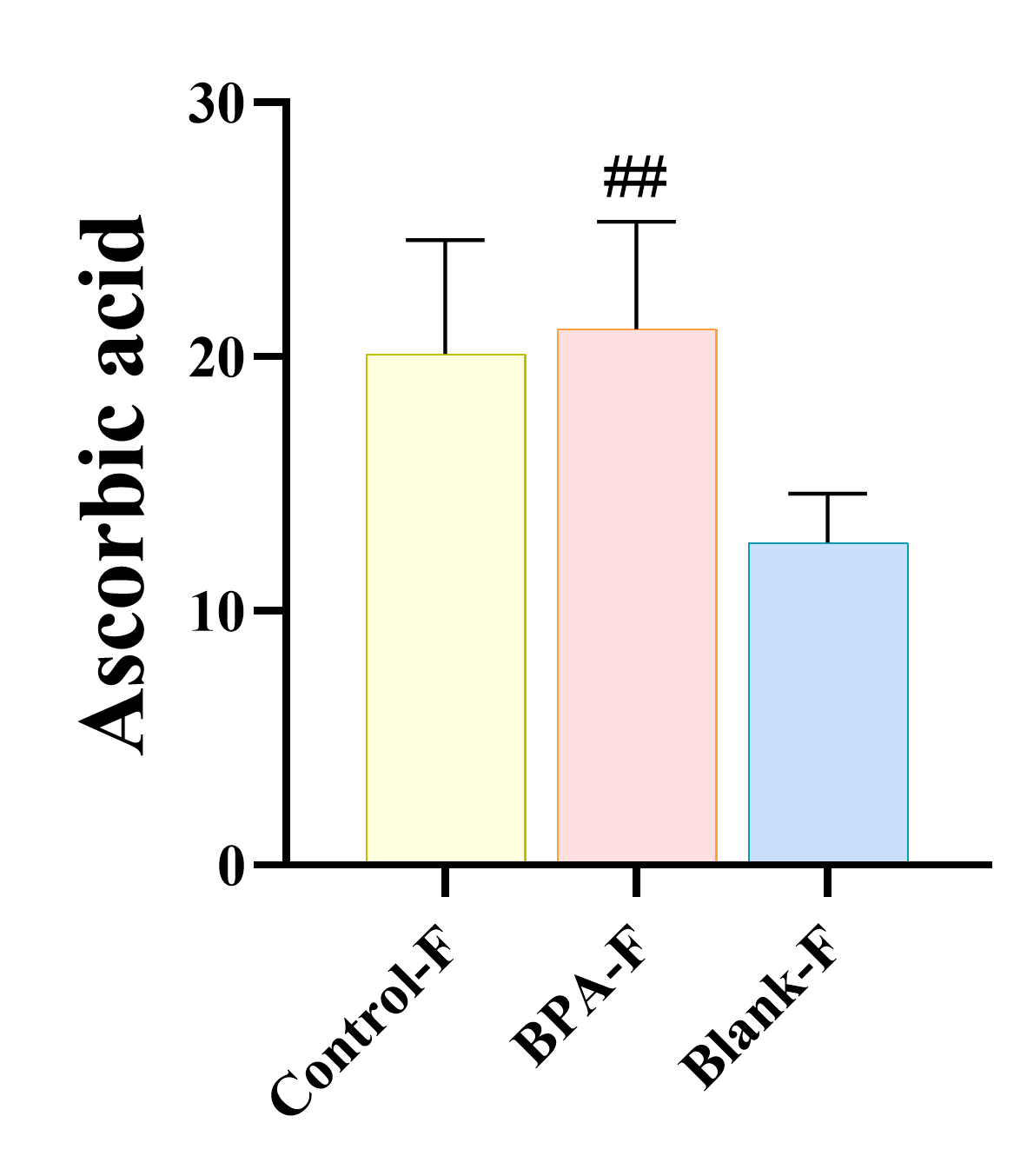

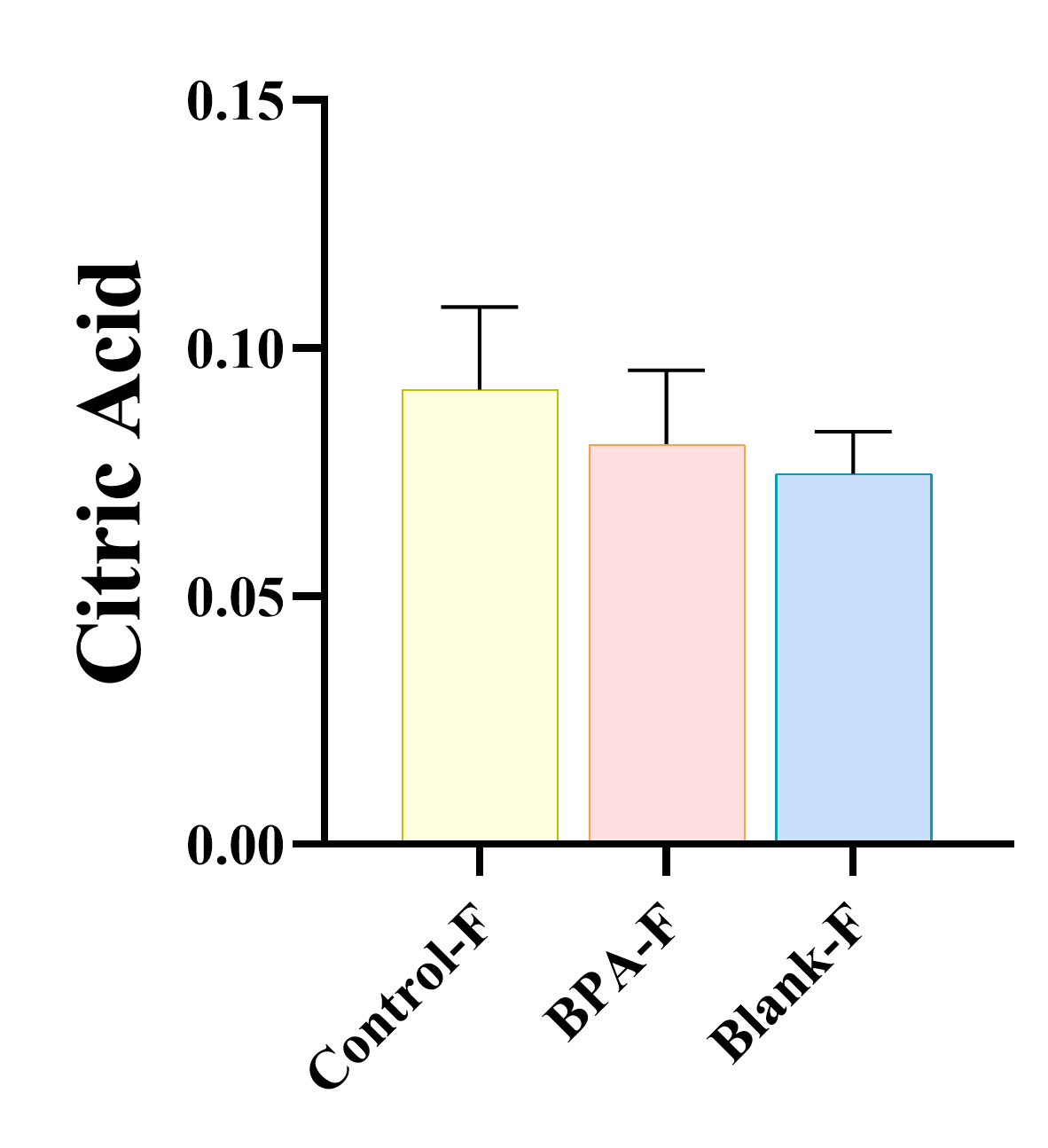


## **Fig. S9.** Relative level of Amino acids, TCA and oxidative stress metabolism in the serum. Male group: Control-M, BPA-M, Blank-M; Female group: Control-F, BPA-F, Blank-F, n=6. The data did not follow a normal distribution was presented as median and range, and analyzed through the Mann-Whitney U test. Others were presented as mean ± SD, and analyzed through ANOVA followed by Dunnett’s multiple comparison test. * p < 0.05, ** p < 0.01 and *** p < 0.001 versus the Control group; ^#^ p < 0.05, ^##^ p < 0.01 and ^###^ p < 0.001 versus the Blank group.

## **Fig. S10.** Relative level of steroid hormone, TCA and oxidative stress metabolism in the serum. Male group: Control-M, BPA-M, Blank-M; Female group: Control-F, BPA-F, Blank-F, n=6. The data did not follow a normal distribution (female group: 17-OHP, Pregnenolone, Progesterone, Estradiol) was presented as median and range, and analyzed through the Mann-Whitney U test. Others were presented as mean ± SD, and analyzed through ANOVA followed by Dunnett’s multiple comparison test. * p < 0.05, ** p < 0.01 and *** p < 0.001 versus the Control group; ^#^ p < 0.05, ^##^ p < 0.01 and ^###^ p < 0.001 versus the Blank group.
